# Computation of Antigenicity Predicts SARS-CoV-2 Vaccine Breakthrough Variants

**DOI:** 10.3389/fimmu.2022.861050

**Published:** 2022-03-24

**Authors:** Ye-Fan Hu, Jing-Chu Hu, Hua-Rui Gong, Antoine Danchin, Ren Sun, Hin Chu, Ivan Fan-Ngai Hung, Kwok Yung Yuen, Kelvin Kai-Wang To, Bao-Zhong Zhang, Thomas Yau, Jian-Dong Huang

**Affiliations:** ^1^School of Biomedical Sciences, Li Ka Shing Faculty of Medicine, University of Hong Kong, Hong Kong, Hong Kong SAR, China; ^2^Department of Medicine, Li Ka Shing Faculty of Medicine, University of Hong Kong, Queen Mary Hospital, Hong Kong, Hong Kong SAR, China; ^3^Chinese Academy of Sciences Key Laboratory of Quantitative Engineering Biology, Shenzhen Institute of Synthetic Biology, Shenzhen Institutes of Advanced Technology, Chinese Academy of Sciences, Shenzhen, China; ^4^Kodikos Labs, Paris, France; ^5^Department of Microbiology, Li Ka Shing Faculty of Medicine, University of Hong Kong, Queen Mary Hospital, Hong Kong, Hong Kong SARS, China; ^6^Guangdong-Hong Kong Joint Laboratory for RNA Medicine, Sun Yat-Sen University, Guangzhou, China

**Keywords:** SARS-CoV-2, variants of concern, antigenicity prediction, vaccine breakthrough variants, computation of antigenicity

## Abstract

It has been reported that multiple severe acute respiratory syndrome coronavirus 2 (SARS-CoV-2) variants of concern (VOCs) including Alpha, Beta, Gamma, and Delta can reduce neutralization by antibodies, resulting in vaccine breakthrough infections. Virus–antiserum neutralization assays are typically performed to monitor potential vaccine breakthrough strains. However, experiment-based methods took several weeks whether newly emerging variants can break through current vaccines or therapeutic antibodies. To address this, we sought to establish a computational model to predict the antigenicity of SARS-CoV-2 variants by sequence alone. In this study, we firstly identified the relationship between the antigenic difference transformed from the amino acid sequence and the antigenic distance from the neutralization titers. Based on this correlation, we obtained a computational model for the receptor-binding domain (RBD) of the spike protein to predict the fold decrease in virus–antiserum neutralization titers with high accuracy (~0.79). Our predicted results were comparable to experimental neutralization titers of variants, including Alpha, Beta, Delta, Gamma, Epsilon, Iota, Kappa, and Lambda, as well as SARS-CoV. Here, we predicted the fold of decrease of Omicron as 17.4-fold less susceptible to neutralization. We visualized all 1,521 SARS-CoV-2 lineages to indicate variants including Mu, B.1.630, B.1.633, B.1.649, and C.1.2, which can induce vaccine breakthrough infections in addition to reported VOCs Beta, Gamma, Delta, and Omicron. Our study offers a quick approach to predict the antigenicity of SARS-CoV-2 variants as soon as they emerge. Furthermore, this approach can facilitate future vaccine updates to cover all major variants. An online version can be accessed at http://jdlab.online.

## Introduction

Up to January 2022, there have been several severe acute respiratory syndrome coronavirus 2 (SARS-CoV-2) variants including B.1.1.7 (Alpha) (1–5), B.1.351 (Beta) (2, 3, 6, 7), P.1 (Gamma) (1, 2, 8), and B.1.617.2 (Delta) (9, 10) that are experimentally tested to lead vaccine breakthrough infections, thus they have been designated as variants of concern (VOCs) by the World Health Organization (WHO). There is a concern that other untested emerging variants may lead to vaccine breakthrough infections (11–16). The most recent case is the validation of B.1.1.529 (Omicron). The current virological and epidemiological techniques took several weeks to validate whether the variant is capable of reducing the efficacy of current vaccines (17, 18) or therapeutic antibodies (18, 19), even though their viral sequences have been shared in real time *via* the Global Initiative for Sharing All Influenza Data (GISAID) (20). The speed of validation of vaccine breakthrough variants can hardly catch up with the fast-emerging rate of new variants. Thus, it is crucial to develop new approaches for identifying the next potential vaccine breakthrough variant as soon as it is reported.

Here, we established a computational approach for predicting the antigenicity of SARS-CoV-2 variants from viral sequences alone, with the aim to accelerate the identification of potential vaccine breakthrough variants. Our approach is founded on the concept of antigenic mapping, also named antigenic cartography. This method has been used to monitor vaccine breakthrough variants of influenza virus using hemagglutination inhibition (HI) assay data (21, 22), dengue virus (23), and SARS-CoV-2 circulating strains (24) using pairwise antiserum data. In antigenic mapping, the antigenic distance is calculated from the fold change of the neutralization titer between the reference virus and its variant to measure the change of antigenicity between two variants. A computational approach for predicting antigenic distances to indicate vaccine breakthrough variants could theoretically provide much more rapid results once the variant sequence is reported. Past studies proposed a linear relationship between amino acid changes in antigenic sites and neutralization fold decrease (25–29). Computational prediction approaches based on such a relationship could also provide reliable estimates of neutralization titers for existing antiserum against the vaccine breakthrough variants with similar accuracy to experiment-based approaches used in previous studies (25–29). However, these predictions were optimized for influenza virus instead of SARS-CoV-2. For example, the neutralization titer decrease of any SARS-CoV-2 variant should be less than that of SARS-CoV compared to the ancestral strain of SARS-CoV-2 because the cross protection between the SARS-CoV-2 variant and the ancestral strain is stronger than that between SARS-CoV and SARS-CoV-2. Thus, it is difficult to use a linear relationship to predict the decrease in neutralization titer that saturates with the increase in the mutation numbers of variants. A SARS-CoV-2 optimized model for predicting antigenicity is urgently needed.

In this study, we established a computational sequence-based method to predict the antigenicity of SARS-CoV-2 variants to reveal potential vaccine breakthrough variants. This method can also predict the neutralization titer of VOCs in comparison to the ancestral strain of SARS-CoV-2. Our predicted results were comparable with experimental neutralization titers of VOCs, including B.1.1.7 (Alpha), B.1.351 (Beta), B.1.617.2 (Delta), B.1.429 (Epsilon), P.1 (Gamma), B.1.526 (Iota), B.1.617.1 (Kappa), and C.37 (Lambda), as well as SARS-CoV. Here, we predicted that B.1.1.529 (Omicron) is 17.4-fold less susceptible to neutralization, which is consistent with reported decrease folds ranging from 10 to 40 (17, 18).

### A Computational Model for Predicting Antigenicity of SARS-CoV-2 Variants

**Figure 1:**
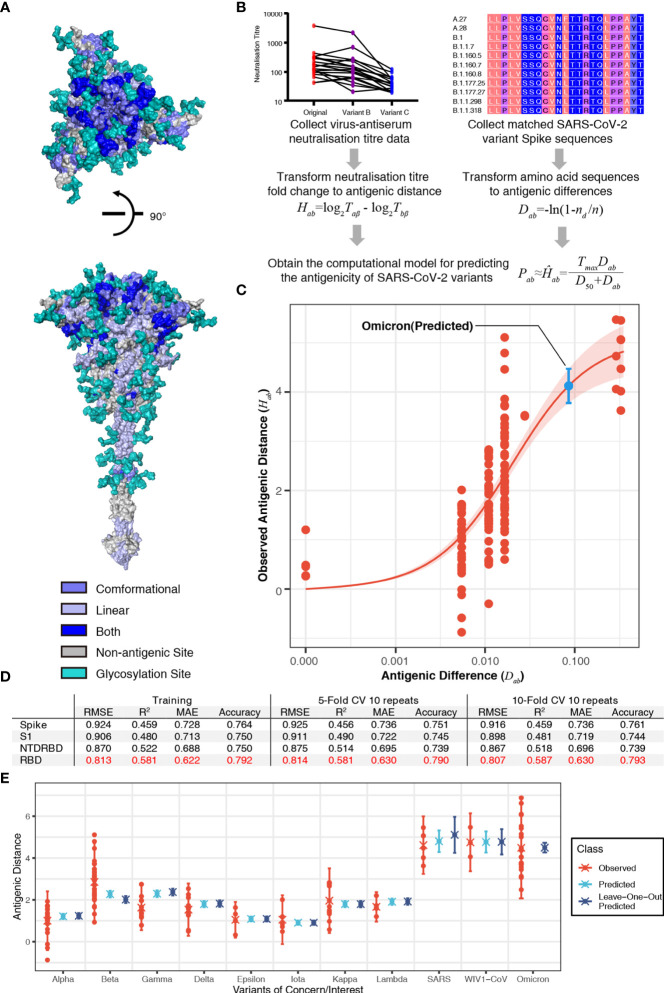
Sequence-based prediction of antigenic distance. **(A)** The top view and the side view of antigenic sites on the full-length Spike protein (30). The conformational epitopes are colored in slate and linear epitopes in light blue. Some antigenic positions in both conformational epitopes and linear epitopes are colored in blue. All glycosylation sites are in teal. **(B)** A flowchart of the process to establish the sequence-based computational model of severe acute respiratory syndrome coronavirus 2 (SARS-CoV-2) antigenicity. The antigenic distance of variant *a* to reference virus *b* from neutralization titer was defined as *H_ab_* = log_2_*T_aβ_* - log_2_*T_bβ_*, where *β*, *T_aβ_*, and *T_bβ_* denote antiserum (referencing virus *b*), the titer of antiserum *β* against virus *b*, and the titer of antiserum *β* against virus *a (* 26*)*. The antigenic distance of variant *a* to reference virus *b* from amino acid sequences was defined as *D_ab_* = *-*ln(1-*n_d_*/*n*), where *n_d_* is the number of amino acid substitutions between variant *a* and reference virus *b*, *n* is the number of antigenic sites. Then, we proposed a relationship between the observed antigenic distance and the antigenic difference: *H_ab_* = *T_max_·D_ab_*/(*D*_50_+*D_ab_*), where *T_max_* is the maximal fold of decrease and *D*_50_ is the antigenic difference that may lead to neutralization decrease at the 50% level of the maximal decrease. **(C)** The relationship between the antigenic difference and the observed antigenic distance. The predicted antigenic distance of B.1.1.529 (Omicron) is marked in cyan. **(D)** The performance of the model in different fragments of the spike protein in terms of root-mean-square error (RMSE), mean absolute error (MAE), coefficient of determination (R^2^), and accuracy. **(E)** Predicted vs. observed antigenic distances of variants of concern. Here, the observed antigenic distances as fold decreases in the neutralization titers of variants of concern vs. the original strain on a log 2 scale. Each point shows the mean of antigenic distances in each assay. Predicted antigenic distances are based on the prediction in panel **(C)** Leave-one-out predicted antigenic distances are predicted based on the datasets without the variant that we aim to compare.

To predict the antigenicity of SARS-CoV-2 variants, we firstly integrated the reported conformational or linear epitopes (**Figure S1**, **Table S1**) on the SARS-CoV-2 Spike protein (**Figure 1A**) with the reported experimental virus–antiserum neutralization titers against SARS-CoV-2 variants including B.1.1.7 (1–5), B.1.351 (2, 3, 6, 7), and P.1 (1, 2, 8) (**Table S2A**). Considering the distinct assays used in the different studies, we standardized the neutralization titers of each variant to the titer of the ancestral strain of SARS-CoV-2 (lineage A) using the same assay in each study on a log 2 scale, and thus we got observed antigenic distance (*H_ab_*) from neutralization titers (**Figure 1B**). For the antigenic difference (*D_ab_*), we used Poisson distance to represent the difference between two amino acid sequences (**Figure 1B**). By comparing the observed antigenic distance with the antigenic difference, we found a relationship between observed antigenic distance and the antigenic difference: *H_ab_* = *T_max_·D_ab_*/(*D*_50_+*D_ab_*), where *T_max_* is the maximal fold of decrease and *D*_50_ is the antigenic difference that may lead to a neutralization decrease at the 50% level of the maximal decrease (the fold change between SARS-CoV-2 and SARS-CoV). This relationship described that the decrease of neutralization titer increases with the accumulation of amino acid changes and then reaches the maximal decrease (**Figures 1C, D**). Based on this correlation, we obtained a computational model using the receptor-binding domain (RBD) of the spike protein to predict the fold decrease in virus–antiserum neutralization titers with higher accuracy (~0.79, the calculation of accuracy in the *Methods*) compared with other fragments of spike (entire spike, N terminal domain plus RBD, or S1; **Figure 1D**). With repeated 5-fold or 10-fold cross validation (**Figure 1D**), we found that prediction using RBD is relatively robust in terms of root-mean-square error (RMSE), mean absolute error (MAE), coefficient of determination (R^2^), and accuracy.

To further validate our model, we predicted the fold decreases in neutralization titers (compared to the ancestral SARS-CoV-2) of multiple variants including B.1.1.7 (Alpha), B.1.351 (Beta), B.1.617.2 (Delta), B.1.429 (Epsilon), P.1 (Gamma), B.1.526 (Iota), B.1.617.1 (Kappa), and C.37 (Lambda), as well as SARS-CoV and WIV1-CoV using datasets without the variant that we aimed to validate. Previous studies have reported that VOCs can elicit vaccine breakthrough infections, which correlated with fold decreases in the neutralization titers from experimental assays (**Table S2**). Our predicted results were highly consistent with the neutralization assay results (**Figure 1E**). We also predicted the fold of decrease in neutralization titer of the most recent VOC, B.1.1.529 (Omicron). Considering 15 mutations in the spike of B.1.1.529 (Omicron), the variant is estimated to have a 17.44-fold (95% confidence interval: 13.7, 22.2) decrease in neutralization titer (shown as a blue point in **Figure 1C**). The predicted result is consistent with reported decrease folds ranging from 10 to 40 (**Figure 1E**) (17, 18). This result shows the risk of vaccine breakthrough or reinfection of B.1.1.529 (Omicron) due to the dramatic decrease in neutralization.

### The Prediction of Potential Vaccine Breakthrough Strains

**Figure 2:**
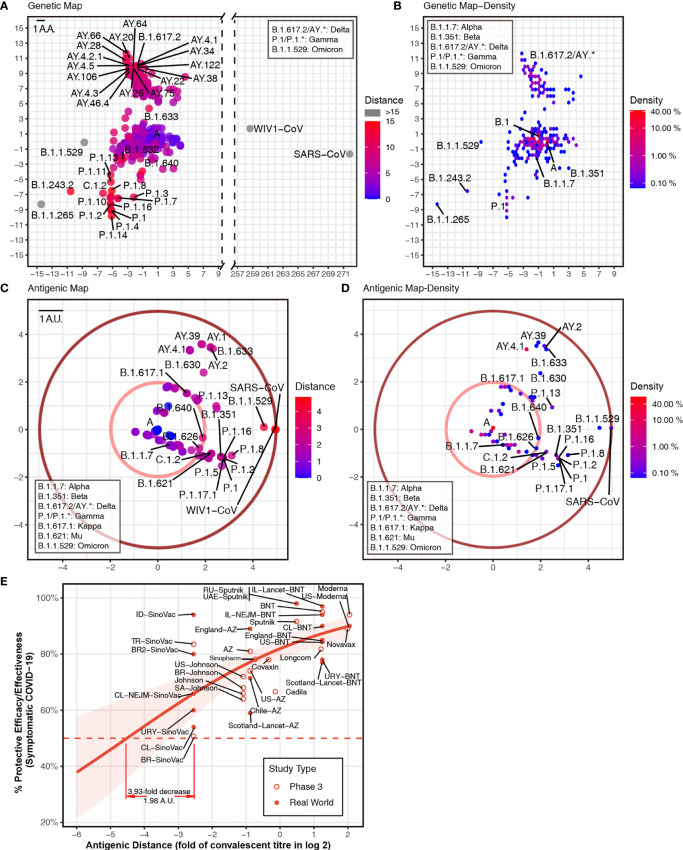
Genetic and antigenic mapping of severe acute respiratory syndrome coronavirus 2 (SARS-CoV-2) variants. **(A)** Genetic map of SARS-CoV-2 variant strains shows amino acid mutation numbers of spike proteins, and **(B)** the density of genetic map shows the distribution of the variants. The vertical and horizontal axes represent the measured relative genetic distances (1 amino acid/1 AA = 1 amino acid difference). **(C)** Antigenic map of SARS-CoV-2 variant strains shows the antigenic distance between variants, and **(D)** the density of antigenic map shows the distribution of the variants. Variants outside the pink circle are vaccine breakthrough candidates. The red circle suggested the border of antigenic map. The antigenic distance is based on receptor-binding domain (RBD) amino acid sequences. The vertical and horizontal axes represent the measured relative antigenic distances (1 arbitrary unit/1 AU = 1-fold decrease in the neutralization titer on a log 2 scale). Colors show the antigenic distance to the SARS-CoV-2 original strain (lineage A). **(E)** Relationship between antigenic distance (mean of neutralization titers in vaccinees divided by corresponding mean of titers in convalescent patients in log 2) and protection from SARS-CoV-2 infection. The reported mean neutralization level from phase 1 or 2 studies (**Table S4**) and the protective efficacy or effectiveness from phase 3 trials or real-world studies (**Table S5**) for different vaccines. The red line indicates the logistic model, and the red shading indicates the 95% confidence interval of the model. Here, we mark the basis of setting up the cutoff of 3.93-fold decrease (1.98 AU).

To predict the next potential SARS-CoV-2 vaccine breakthrough variants, we visualzsed the antigenicity of all available SARS-CoV-2 variants as an indicator of their vaccine breakthrough potential. We firstly selected all 1,521 lineage variants using PANGO (31) updated on December 6, 2021 (**Table S3**), to predict their antigenicity. Then, we calculated the pairwise distances of different variants. For visualizing these results, we captured two principal components from the high-dimensional data of antigenic distance (25). We used all spike amino acid sequences to plot the “genetic map” of SARS-CoV-2 to represent the genetic difference among different variants (**Figures 2A, B**). We then plotted the “antigenic map” using the predicted antigenic distances (**Figures 2C, D**; online versions are available at http://jdlab.online).

Based on the relationship between neutralization titer fold change and protective efficacy (32), it was convenient to set up some “cutoffs” in the current vaccine coverage. We included phase 3 and real-world results of vaccine efficacy or effectiveness, as well as neutralization titer data from phase 1 and 2 studies (**Tables S4, S5**). Thus, we got the relationship between neutralization titer and protective efficacy against a symptomatic coronavirus disease 2019 (COVID-19) (**Figure 2E**). A 3.93-fold decrease in neutralization titers that is induced by VOCs can dampen the efficacy of some vaccines to lower than 50%. In this way, one cutoff of 1.98 arbitrary units (AU) represented a 3.93-fold decrease in the neutralization titer (shown as a pink circle in **Figures 2C, D**). All variants outside this cutoff have the potential to be vaccine breakthrough variants. By comparing the “genetic map” and antigenic map, we can set up the border of antigenic map. Although there are >200 mutations in the SARS-CoV and WIV1-CoV spike (**Figure 2A**), the antigenic distance is around 4.9 AU, which means ~30-fold decrease in the neutralization titer (shown as a dark red circle in **Figures 2C, D**).

To reveal the distribution of the variant, we plotted the density of variants on the “genetic map” and antigenic map due to overlapping dots. In the genetic map, hotspots are located at lineage A (>10%) and B.1 (>40%) mainly, as well as AY.* and P.1 (**Figure 2B**). While in the antigenic map, hotspots are placed at lineage A (>40%) mainly, together with AY.* (**Figure 2D**). Although most variants were shown to be close to the ancestral strain (**Figures 2B, D**), multiple variants were found to decrease neutralization titers significantly (**Figure 2C**). In addition to reported VOCs including B.1.351 (Beta, containing sublineages like B.1.351.2 and B.1.351.5) (2, 3, 6, 7), P.1 (Gamma, containing sublineages like P.1.11 and P.1.3) (1, 2, 8), B.1.617.2 (Delta, containing sublineages AY.*) (9), and B.1.621 (Mu, containing sublineage B.1.621.1), and B.1.1.529 (Omicron) showed over 3.93-fold decrease in the neutralization titer. Other variants B.1.630, B.1.633, B.1.649, and C.1.2 also have the potential to be vaccine breakthrough variants with more than 3.93-fold decrease (**Figure 2C**). Besides the pandemic of B.1.617.2 (Delta) (9) and the outbreak of B.1.1.529 (Omicron), multiple variants should be investigated immediately, as they have the potential to become tomorrow’s VOCs.

## Discussion

In this study, we established a computational approach to predict the fold decrease of neutralization titers against multiple variants including Alpha, Beta, Gamma, Delta, and Omicron. Our computational approach could potentially provide the first hints of whether a newly identified variant can break through vaccines just by its sequence information, which would greatly shorten the time for the crucial early warning of emerging vaccine breakthrough strains. Predicting neutralization responses against all variants based on sequences alone is also vital for SARS-CoV-2, requiring multiple vaccinations for protection currently.

Our computation model set a method of measuring the immune escape or vaccine breakthrough of SARS-CoV-2 variants, considering the cross reaction between SARS-CoV and SARS-CoV-2. In our prediction of the antigenicity, we proposed that the limit of neutralization titer decrease is set by SARS-CoV (**Figure 1**). In recent studies, SARS-CoV is ~36-fold less susceptible to neutralization compared to the ancestral strain of SARS-CoV-2, which suggested no cross reaction. Based on this result, a non-linear curve was established to describe the relationship between the observed antigenic distance and the antigenic difference. We further performed calculations using different fragments of the Spike protein (**Figure 1D**). Among the Spike protein and the RBD, N-terminal domain (NTD)-RBD, and S1 fragments, we found that the prediction using amino acid sequences of RBD was able to estimate the neutralization titer more accurately than the others (**Figure 1D**). Thus, we used the RBD-based computations to determine the neutralization titers.

In this study, our methods provide a relatively stable reference of the fold change of neutralization titer to compare results of different variants as a potential control. A major concern of using neutralization titer decrease is that the data are based on diverse neutralization assays of serum samples from both patients and vaccinees against both live virus and pseudovirus (**Table S2**). Although the results were consistent qualitatively, the variation of fold change is too large to be ignored (**Figure 1E**). Considering the variation in the real world and previous studies (28), we set up values 2-fold or less than the experimental values as the criteria to calculate the accuracy of our model (see *Methods* for details). It is better to establish a convenient and standardized neutralization pipeline in the future like the hemagglutination inhibition (HI) assay for influenza virus. Since our prediction results offer the median values of fold change, it will be easier to build a standardized neutralization pipeline using our methods as a reference. Such a pipeline can allow the precise estimation of neutralization titers. Together with estimating the association of neutralization with protection, it will help to develop next-generation vaccines.

Our computation methods allow us to study all variants of SARS-CoV-2 to focus on several potential vaccine breakthrough strains. Under current COVID-19 pandemic, it is crucial to update vaccines to cover all vaccine breakthrough strains that have significant amino acid and glycosylation changes to prevent further infectious outbreaks. However, not all predicted SARS-CoV-2 vaccine breakthrough variants will have the chance to cause an outbreak due to their changed viral fitness (33) or by pure luck. Based on previous studies of influenza viruses, it is possible for variants to have alterations that change the antigenicity but fail to cause outbreaks in the wider population (34). Considering immune escape elicited by variants, updating current vaccine seeds with new variants should extend the vaccine coverage. As SARS-CoV-2 showed different variant directions in the antigenic map (**Figure 2**), the use of multiple virus seeds based on the different directions might be appropriate to cover all major variants in the long term. Our method could help in the selection of SARS-CoV-2 variants for updating vaccines.

## Methods

### Antigenic Footprint

We collected 149 confirmed conformational epitopes with protein structures released in the Protein Data Bank (PDB) (https://www.rcsb.org/) or annotated epitope footprints and 76 linear epitopes published in the literature (**Table S1**). We plotted the footprint of all Spike protein epitopes from the aforementioned 225 epitopes using R-3.6.6.

### Antigenic Distances From Neutralization Data

We calculated antigenic distances from the neutralization data based on previous publications (26). For virus variant *a*, reference virus *b*, and antiserum *β* (referencing virus *b*), we defined the antigenic distance of variant *a* to reference virus *b* in terms of the standardized log titer as *H_ab_* = log_2_*T_aβ_* - log_2_*T_bβ_*, where *T_bβ_* is the titer of antiserum *β* against virus *b*, and *T_aβ_* is the titer of antiserum *β* against virus *a* (26). Merged data with reference virus lineage A (the ancestral strain of SARS-CoV-2) were collected from several publications (**Table S2**).

### Genetic and Antigenic Difference Calculation

We selected 1,521 SARS-CoV-2 lineages using PANGO (v.3.1.15) updated on December 6, 2021 (https://cov-lineages.org/). Spike protein amino acid sequences of these lineages were obtained from GISAID using the earliest collected for each lineage (**Table S3**). All sequences with neutralization titers were also included (**Table S3**). For genetic distances, we used Molecular Evolutionary Genetics Analysis (MEGA) X to calculate the pairwise distances among Spike protein amino acid sequences in the SARS-CoV-2 variants using a Poisson model. For antigenic distance, we used an information theory-based approach *p-all-epitope* (27, 28) to measure the pairwise distances among amino acid sequences of the antigenic footprint (“antigenic positions”). The distance is based on the number of different amino acids *n_d_* between two *n*-mer viral sequences of variants *a* and *b*. Under the assumption that the number of amino acid substitutions per site follows a Poisson distribution, we can then calculate the distance between *a* and *b* as *D_ab_* = *-*ln(1-*n_d_*/*n*).

### Modeling and Performance Measurement

A model considering the maximal neutralization titer decrease was applied to examine the antigenic distance from the neutralization data *H_ab_* and our computed results *D_ab_* as *H_ab_* = *T_max_·D_ab_*/(*D*_50_+*D_ab_*), where *T_max_* is the maximal decrease and *D*_50_ is the antigenic difference that may lead to a neutralization decrease at the 50% level of the maximal decrease. The predicted neutralization titer is then given as *P_ab_* ≈*Ĥ_ab_* = *T_max_·D_ab_*/(*D*_50_+*D_ab_*). RMSE, MAE, and coefficient of determination (R^2^) were used to measure the performance of the linear correlation.

Reproducibility was determined by pairwise sequences and neutralization titers. Neutralization titer data were converted into variables by calculating the relative difference in the neutralization titers between reference virus and variant against the antiserum. Accuracy was the percentage of correctly predicted neutralization titers using amino acid sequences. Based on previous studies (28), computational values 2-fold or less than the experimental values were considered to be similar (correct) and those more than 2-fold lower were considered dissimilar (error). Here, 10-time repeated 5-fold and 10-fold cross validation was applied in terms of RMSE, MAE, coefficient of determination (R^2^), and accuracy.

### Genetic and Antigenic Maps

After calculating genetic and antigenic distances, we used classical multidimensional scaling (CMDS) to display the data as a plot using R-3.6.6. We set up SARS-CoV-2 lineage A as the origin and scaled the data in two and three dimensions. We then acquired the genetic and antigenic maps of SARS-CoV-2 lineages. An online version can be obtained at http://jdlab.online.

### Logistic Model

Following past studies (32), we used a logistic model in R-3.6.6 to describe the relationship between antigenic distance (neutralization level) and protective efficacy/effectiveness: *E* = 1/(1+ exp(*−k*(*H−H_50_*))). *E* is the protective efficacy/effectiveness at a specific neutralization level *H*. *H* is the mean of neutralization titers in vaccinees divided by corresponding mean of titers in convalescent patients, which is the antigenic distance to convalescent patients in log 2. *H_50_* is the antigenic distance at which an individual will have a 50% protective efficacy/effectiveness.

## Data Availability Statement

All sequence data listed in **Table S3** are from GISAID’s EpiCoV Database.

## Author Contributions

Y-FH, B-ZZ, TY, HC, KK-WT, and J-DH designed the study. Y-FH and J-CH analyzed the sequences from GISAID and performed the computations and built the online tool. IF-NH and KK-WT recruited the patients and volunteers and collected samples from patients and volunteers and performed the neutralization assay. Y-FH, AD, RS, KYY, KK-WT, H-RG, and J-DH analyzed the results. Y-FH, AD, TY, and J-DH wrote the initial draft, and all authors edited the final version.

## Funding

The work was supported by grants from the Health and Medical Research Fund, the Food and Health Bureau, The Government of the Hong Kong Special Administrative Region (COVID190117, COVID1903010), and Guangdong Science and Technology Department (2020B1212030004) to JH. JH thanks the L & T Charitable Foundation and the Program for Guangdong Introducing Innovative and Entrepreneurial Teams (2019BT02Y198) for their support.

## Conflict of Interest

AD is a founder of Meletios Therapeutics a company working on antiviral drug candidates.

The remaining authors declare that the research was conducted in the absence of any commercial or financial relationships that could be construed as a potential conflict of interest.

## Publisher’s Note

All claims expressed in this article are solely those of the authors and do not necessarily represent those of their affiliated organizations, or those of the publisher, the editors and the reviewers. Any product that may be evaluated in this article, or claim that may be made by its manufacturer, is not guaranteed or endorsed by the publisher.
