# Supplementary material for: Computation of Antigenicity Predicts SARS-CoV-2 Vaccine Breakthrough Variants
Source: Front Immunol. 2022 Mar 24;13:861050. doi: 10.3389/fimmu.2022.861050 (PMC8987580; doi:10.3389/fimmu.2022.861050)
Supplement: Supplementary file 1 [file DataSheet_1.docx]

**Title:** **Computation of Antigenicity Predicts SARS-CoV-2 Vaccine Breakthrough Variants**

**Authors:**

Ye-fan Hu^1,3,†^, Jing-chu Hu^2,†^, Hua-rui Gong^1,†^, Antoine Danchin^1,5^, Ren Sun^1^, Hin Chu^4^, Ivan Fan-Ngai Hung^3^, Kwok Yung Yuen^4^, Kelvin Kai-Wang To^4,^, Bao-zhong Zhang^2,*^, Thomas Yau^3,*^, Jian-Dong Huang^1,2,6,*^

**Affiliations:**

^1^ School of Biomedical Sciences, Li Ka Shing Faculty of Medicine, University of Hong Kong, 3/F, Laboratory Block, 21 Sassoon Road, Hong Kong, China

^2^ CAS Key Laboratory of Quantitative Engineering Biology, Shenzhen Institute of Synthetic Biology, Shenzhen Institutes of Advanced Technology, Chinese Academy of Sciences, Shenzhen 518055, China

^3^ Department of Medicine, Li Ka Shing Faculty of Medicine, University of Hong Kong, 4/F Professional Block, Queen Mary Hospital, 102 Pokfulam Road, Hong Kong, China

^4^ Department of Microbiology, Li Ka Shing Faculty of Medicine, University of Hong Kong, 19/F T Block, Queen Mary Hospital, 102 Pokfulam Road, Hong Kong, China

^5^ Kodikos Labs / Stellate Therapeutics, Institut Cochin, 24 rue du Faubourg Saint-Jacques, 75014 Paris, France

^6^ Guangdong-Hong Kong Joint Laboratory for RNA Medicine, Sun Yat-Sen University,

Guangzhou 510120, China

^†^ These authors contributed equally to this work.

* Corresponding authors. K.K.W.T. ([kelvinto@hku.hk](mailto:kelvinto@hku.hk)), T.Y. ([tyaucc@hku.hk](mailto:tyaucc@hku.hk)), J.D.H. ([jdhuang@hku.hk](mailto:jdhuang@hku.hk))

**Supplementary Figures & Tables**


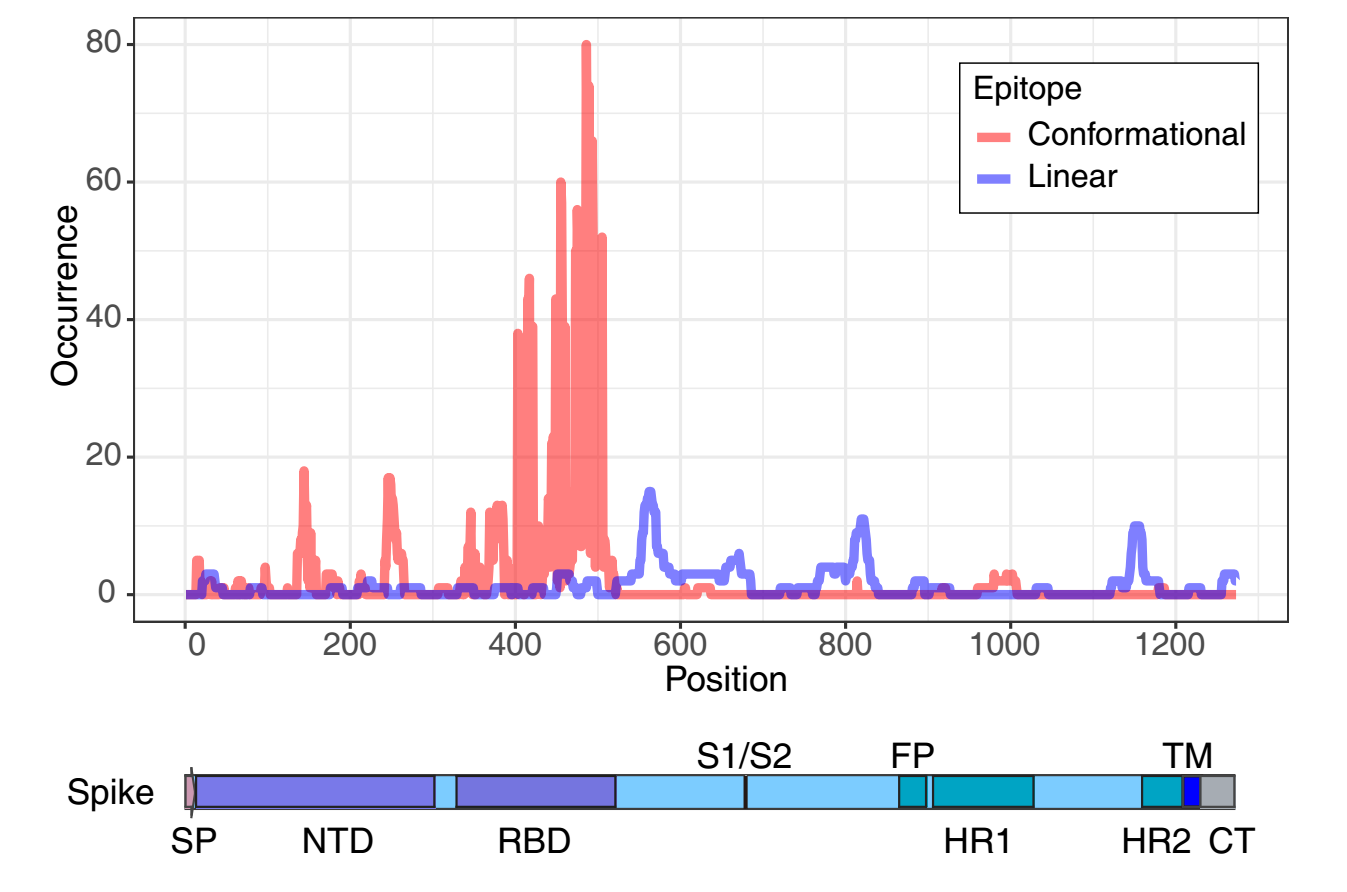


**Figure S1. The epitope mapping of SARS-CoV-2.** Per-residue antigenicity of the Spike protein was plotted by epitope frequency of conformational (N=149) and linear (N=74) epitopes, as well as schematic of the Spike protein coloured by domain. The occurrence showed the number of epitopes containing each position in Table S1. SP, signal peptide; NTD, N-terminal domain; RBD, receptor binding domain; S1/S2, S1/S2 protease cleavage sites; FP, fusion peptide; HR1, heptad repeat 1; HR2, heptad repeat 2; TM, transmembrane domain; CT, cytoplasmic tail.

**Table S1. Reported epitopes on SARS-CoV-2 spike protein.**

**Table S2. Observed neutralisation titres of SARS-CoV-2 variants.**

**Table S3. SARS-CoV-2 strains analysed in our study.**

**Table S4. Data sources for Immunogenicity Data**

| **Manufacturer** | **Vaccine** | **NAbFold** | **Reference** |
| --- | --- | --- | --- |
| AstraZeneca | ChAdOx1 nCoV-19 | 0.542 | 10.1016/S0140-6736(20)31604-4 |
| BioNTech | BNT162b2 | 2.372 | 10.1056/NEJMoa2027906 |
| Cadila | ZyCoV-D | 0.907 | 10.1016/j.eclinm.2021.101020 |
| Covaxin | BBV152 | 0.792 | 10.1016/S1473-3099(21)00070-0 |
| Johnson | Ad26.COV2.S | 0.471 | 10.1056/NEJMoa2034201 |
| Longcom | ZF2001 | 2.314 | 10.1016/S1473-3099(21)00127-4 |
| Moderna | mRNA-1273 | 4.139 | 10.1056/NEJMoa2022483 |
| Novavax | NVX-CoV2373 | 3.974 | 10.1056/NEJMoa2026920 |
| Sinopharm | BBIBP-CorV | 0.600 | 10.1016/S1473-3099(20)30831-8 |
| SinoVac | CoronaVac | 0.171 | 10.1016/S1473-3099(20)30843-4 |
| Sputnik | rAd26-S+rAd5-S | 1.400 | 10.1016/S0140-6736(20)31866-3 |

**Table S5. Data sources for Efficacy Data**

**The online tool**

We developed an interactive online tool (<http://jdlab.online>) for the dynamic prediction of the antigenicity of a newly emerging SARS-CoV-2 variants plotted as an antigenic map.

**
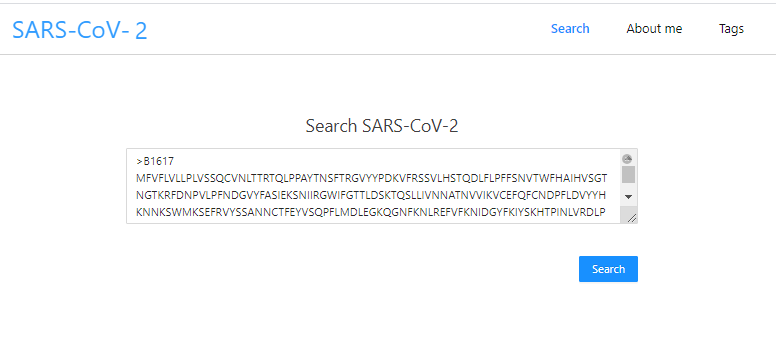
**

**Figure S3a. The initial interface of the online tool.**

In the online tool, users can search for a variant of interest by inputting one or multiple SARS-CoV-2 Spike amino acid sequences in a fasta format.

**
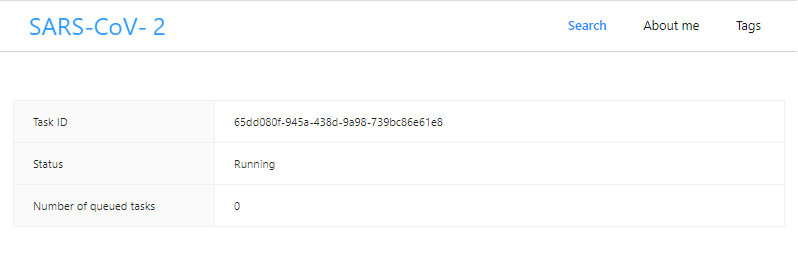
**

**Figure S3b. The processing interface of the online tool.**

After the search task has completed, the interface then presents the genetic and antigenic maps of the SARS-CoV-2 variants. The two figures above are the genetic distance maps of SARS-CoV-2 variant strains in the Spike protein amino acid sequences and the antigenic distance map of SARS-CoV-2 variant strains in the antigenic epitope amino acid sequences. The other two figures below are the genetic distance maps of SARS-CoV-2 variant strains in the N-terminal domain (NTD) and receptor binding domain (RBD) amino acid sequences and the antigenic distance map of SARS-CoV-2 variant strains in the NTD and RBD amino acid sequences.

**
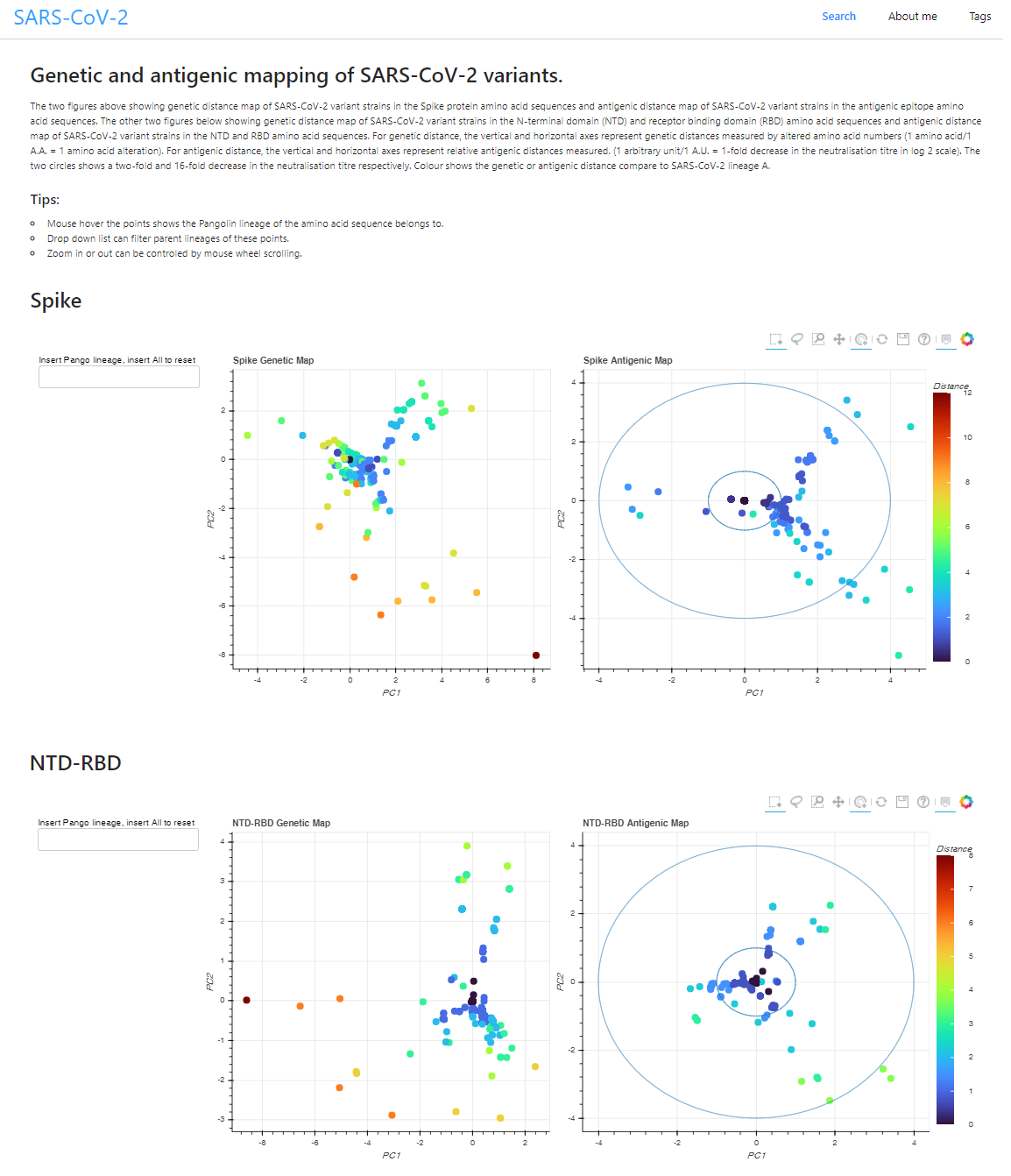
**

**Figure S3c. The result interface of the online tool.**

For genetic distance, the vertical and horizontal axes represent genetic distances measured by altered amino acid numbers (1 amino acid/1 A.A. = 1 amino acid alteration). For antigenic distance, the vertical and horizontal axes represent the measured relative antigenic distances (1 arbitrary unit/1 A.U. = 1-fold decrease in the neutralization titre in log 2 scale). The two circles show the 2-fold and 16-fold decrease in the neutralization titre, respectively. The colours represent the genetic or antigenic distance compared with SARS-CoV-2 lineage A.

**
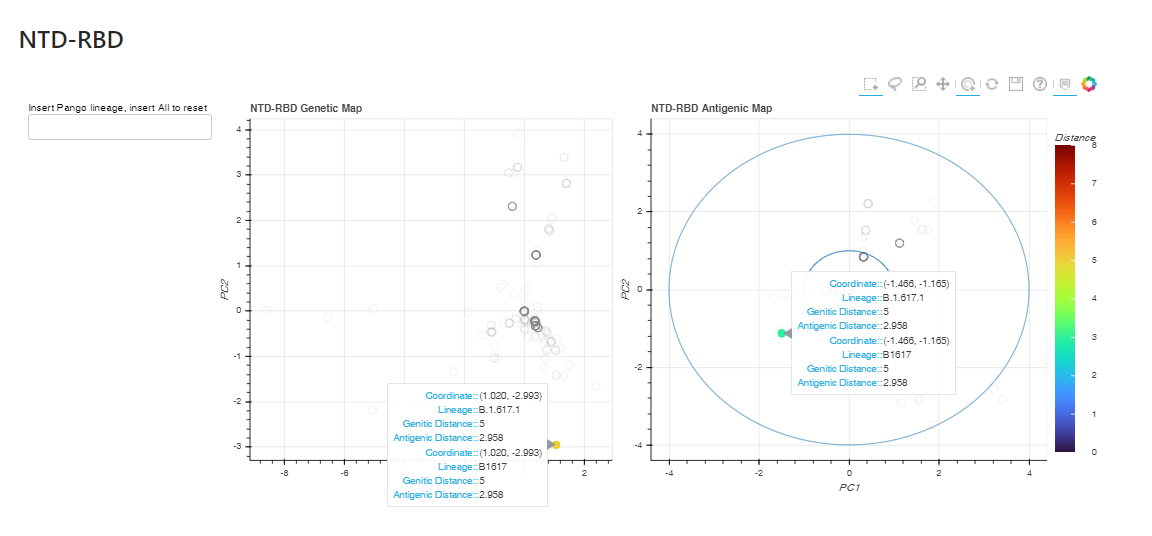
**

**Figure S3d. The interactive functions of the online tool.**

The Pangolin lineage of the amino acid sequence is shown by hovering the mouse over the points. Strains can be highlighted by searching lineage or user input sequence ID in the text search box. The mouse wheel can be used to zoom in or out.
