# Supplementary material for: Computation of Antigenicity Predicts SARS-CoV-2 Vaccine Breakthrough Variants
Source: Front Immunol. 2022 Mar 24;13:861050. doi: 10.3389/fimmu.2022.861050 (PMC8987580; doi:10.3389/fimmu.2022.861050)
Supplement: Supplementary file 3 [file Table_2.pdf]

Table S2. Observed neutralisation titres of SARS-CoV-2 variants
(a) Training Set

Serum	VirusRef	VirusVar	WHO	Type	log2FC	Mean	N	Source	DOI
SinoVac	A	B.1.1.7	Alpha	Pseudovirus ID50	1.02	2.03	75	Acevedo ML, et al	10.1101/2021.06.28.21259673
SinoVac	A	P.1	Gamma	Pseudovirus ID50	1.22	2.33	75	Acevedo ML, et al	10.1101/2021.06.28.21259673
SinoVac	A	C.37	Lambda	Pseudovirus ID50	1.61	3.05	75	Acevedo ML, et al	10.1101/2021.06.28.21259673
SinoVac	A	B.1(D614G)	Non-VOC	Pseudovirus ID50	0.45	1.37	75	Acevedo ML, et al	10.1101/2021.06.28.21259673
BioNTech/ Mo	A	B.1.1.7(E484K)	Alpha	Live Virus FRNT50	1.93	3.80	30	Carreno, et al	10.1101/2021.07.21.21260961
BioNTech/ Mo	A	B.1.351	Beta	Live Virus FRNT50	2.07	4.20	30	Carreno, et al	10.1101/2021.07.21.21260961
BioNTech/Moc	A	B.1.617.2	Delta	Live Virus FRNT50	1.58	3.00	30	Carreno, et al	10.1101/2021.07.21.21260961
BioNTech/Moc	A	B.1.526	Iota	Live Virus FRNT50	0.49	1.40	30	Carreno, et al	10.1101/2021.07.21.21260961
BioNTech/Moc	A	B.1.526.2	Iota	Live Virus FRNT50	1.20	2.30	30	Carreno, et al	10.1101/2021.07.21.21260961
BioNTech/Moc	A	B.1.526(E484K)	Iota	Live Virus FRNT50	0.85	1.80	30	Carreno, et al	10.1101/2021.07.21.21260961
BioNTech/Moc	A	C.37	Lambda	Live Virus FRNT50	2.20	4.60	30	Carreno, et al	10.1101/2021.07.21.21260961
Patient	B.1.1.117	B.1.351	Beta	Live Virus FRNT50	3.07	8.37	14	Cele, et al	10.1038/s41586-021-03471-w
Patient	B.1.351	B.1.1.117	Beta	Live Virus FRNT50	2.05	4.14	6	Cele, et al	10.1038/s41586-021-03471-w
SinoVac	A	B.1.1.7	Alpha	Pseudovirus NT50	0.59	1.51	76	Chen Y, et al	10.1016/S1473-3099(21)00287-5
SinoVac	A	B.1.351	Beta	Pseudovirus NT50	2.40	5.27	76	Chen Y, et al	10.1016/S1473-3099(21)00287-5
SinoVac	A	B.1.429	Epsilon	Pseudovirus NT50	0.32	1.25	76	Chen Y, et al	10.1016/S1473-3099(21)00287-5
SinoVac	A	P.1	Gamma	Pseudovirus NT50	1.97	3.92	76	Chen Y, et al	10.1016/S1473-3099(21)00287-5
SinoVac	A	B.1.526	Iota	Pseudovirus NT50	2.01	4.03	76	Chen Y, et al	10.1016/S1473-3099(21)00287-5
SinoVac	A	B.1(D614G)	Non-VOC	Pseudovirus NT50	0.28	1.21	76	Chen Y, et al	10.1016/S1473-3099(21)00287-5
Patient	B.1(D614G)	B.1.1.7	Alpha	Pseudovirus EC50	0.43	1.35	19	Chen, et al	10.1038/s41591-021-01294-w
BioNTech	B.1(D614G)	B.1.1.7	Alpha	Pseudovirus EC50	1.02	2.03	24	Chen, et al	10.1038/s41591-021-01294-w
Patient	B.1(D614G)	B.1.351	Beta	Pseudovirus EC50	2.20	4.58	19	Chen, et al	10.1038/s41591-021-01294-w
BioNTech	B.1(D614G)	B.1.351	Beta	Pseudovirus EC50	3.35	10.20	24	Chen, et al	10.1038/s41591-021-01294-w
Patient	B.1(D614G)	P.1	Gamma	Pseudovirus EC50	1.33	2.52	10	Chen, et al	10.1038/s41591-021-01294-w
BioNTech	B.1(D614G)	P.1	Gamma	Pseudovirus EC50	1.16	2.23	15	Chen, et al	10.1038/s41591-021-01294-w
Patient	B.1(D614G)	K417N/D614G	Non-VOC	Pseudovirus EC50	-0.58	0.67	19	Chen, et al	10.1038/s41591-021-01294-w
BioNTech	B.1(D614G)	K417N/D614G	Non-VOC	Pseudovirus EC50	-0.01	0.99	24	Chen, et al	10.1038/s41591-021-01294-w
Patient	B.1(D614G)	E484K/N501Y/D6	Non-VOC	Pseudovirus EC50	2.36	5.12	19	Chen, et al	10.1038/s41591-021-01294-w
BioNTech	B.1(D614G)	E484K/N501Y/D6	Non-VOC	Pseudovirus EC50	2.06	4.17	24	Chen, et al	10.1038/s41591-021-01294-w
Patient	B.1(D614G)	K417N/E484K/N5	Non-VOC	Pseudovirus EC50	1.83	3.55	19	Chen, et al	10.1038/s41591-021-01294-w

Patient	A	B.1.617.1	Kappa	Live Virus FRNT50	2.66	6.32	24	Edara, et al	10.1056/NEJMc2107799
Moderna	A	B.1.617.1	Kappa	Live Virus FRNT50	2.77	6.82	15	Edara, et al	10.1056/NEJMc2107799
BioNTech	A	B.1.617.1	Kappa	Live Virus FRNT50	2.83	7.12	10	Edara, et al	10.1056/NEJMc2107799
BioNTech	A	B.1.1.7	Alpha	Pseudovirus NT50	1.07	2.10	30	Garcia-Beltran, et al	10.1016/j.cell.2021.03.013
Moderna	A	B.1.1.7	Alpha	Pseudovirus NT50	1.20	2.30	35	Garcia-Beltran, et al	10.1016/j.cell.2021.03.013
BioNTech	A	B.1.351	Beta	Pseudovirus NT50	5.11	34.50	30	Garcia-Beltran, et al	10.1016/j.cell.2021.03.013
Moderna	A	B.1.351	Beta	Pseudovirus NT50	4.79	27.70	35	Garcia-Beltran, et al	10.1016/j.cell.2021.03.013
BioNTech	A	B.1.429	Epsilon	Pseudovirus NT50	1.00	2.00	30	Garcia-Beltran, et al	10.1016/j.cell.2021.03.013
Moderna	A	B.1.429	Epsilon	Pseudovirus NT50	1.00	2.00	35	Garcia-Beltran, et al	10.1016/j.cell.2021.03.013
BioNTech	A	P.1	Gamma	Pseudovirus NT50	2.74	6.70	30	Garcia-Beltran, et al	10.1016/j.cell.2021.03.013
Moderna	A	P.1	Gamma	Pseudovirus NT50	2.17	4.50	35	Garcia-Beltran, et al	10.1016/j.cell.2021.03.013
BioNTech	A	B.1(D614G)	Non-VOC	Pseudovirus NT50	0.26	1.20	30	Garcia-Beltran, et al	10.1016/j.cell.2021.03.013
Moderna	A	B.1(D614G)	Non-VOC	Pseudovirus NT50	0.26	1.20	35	Garcia-Beltran, et al	10.1016/j.cell.2021.03.013
BioNTech	A	B.1.1.298	Non-VOC	Pseudovirus NT50	0.49	1.40	30	Garcia-Beltran, et al	10.1016/j.cell.2021.03.013
Moderna	A	B.1.1.298	Non-VOC	Pseudovirus NT50	0.38	1.30	35	Garcia-Beltran, et al	10.1016/j.cell.2021.03.013
BioNTech	A	WIV1-CoV	WIV1-CoV	Pseudovirus EC50	5.47	44.30	30	Garcia-Beltran, et al	10.1016/j.cell.2021.03.013
Moderna	A	WIV1-CoV	WIV1-CoV	Pseudovirus EC50	4.73	26.50	35	Garcia-Beltran, et al	10.1016/j.cell.2021.03.013
BioNTech	A	SARS-CoV	SARS	Pseudovirus EC50	5.45	43.80	30	Garcia-Beltran, et al	10.1016/j.cell.2021.03.013
Moderna	A	SARS-CoV	SARS	Pseudovirus EC50	5.07	33.50	35	Garcia-Beltran, et al	10.1016/j.cell.2021.03.013
Patient	A	B.1.351	Beta	Live Virus NT50	2.52	5.75	15	Hoffman, et al	10.1016/j.celrep.2021.109415
BioNTech	A	B.1.351	Beta	Live Virus NT50	3.48	11.13	15	Hoffman, et al	10.1016/j.celrep.2021.109415
Patient	A	B.1.617	Kappa	Live Virus NT50	0.97	1.96	15	Hoffman, et al	10.1016/j.celrep.2021.109415
BioNTech	A	B.1.617	Kappa	Live Virus NT50	1.50	2.83	15	Hoffman, et al	10.1016/j.celrep.2021.109415
BioNTech	A	B.1.617.2	Delta	Live Virus FRNT50	0.50	1.41	20	Liu J-y, et al	10.1038/s41586-021-03693-y
BioNTech	A	B.1.617.2	Delta	Live Virus FRNT50	0.55	1.46	20	Liu J-y, et al	10.1038/s41586-021-03693-y
BioNTech	A	B.1.617.1	Kappa	Live Virus FRNT50	1.68	3.20	20	Liu J-y, et al	10.1038/s41586-021-03693-y
BioNTech	A	B.1.525	Non-VOC	Live Virus FRNT50	0.65	1.57	20	Liu J-y, et al	10.1038/s41586-021-03693-y
BioNTech	A	B.1.618	Non-VOC	Live Virus FRNT50	0.60	1.52	20	Liu J-y, et al	10.1038/s41586-021-03693-y
Patient	A	B.1.1.7	Alpha	Live Virus FRNT50	1.56	2.94	34	Liu, et al	10.1016/j.cell.2021.06.020
Patient	B.1.1.7	A	Alpha	Live Virus FRNT50	-0.88	0.54	18	Liu, et al	10.1016/j.cell.2021.06.020
BioNTech	A	B.1.1.7	Alpha	Live Virus FRNT50	1.71	3.28	25	Liu, et al	10.1016/j.cell.2021.06.020
AstraZeneca	A	B.1.1.7	Alpha	Live Virus FRNT50	1.22	2.34	25	Liu, et al	10.1016/j.cell.2021.06.020
Patient	A	B.1.351	Beta	Live Virus FRNT50	3.74	13.32	34	Liu, et al	10.1016/j.cell.2021.06.020

Patient	B.1.351	A	Beta	Live Virus FRNT50	0.93	1.91	14	Liu, et al	10.1016/j.cell.2021.06.020
BioNTech	A	B.1.351	Beta	Live Virus FRNT50	2.92	7.57	25	Liu, et al	10.1016/j.cell.2021.06.020
AstraZeneca	A	B.1.351	Beta	Live Virus FRNT50	3.17	9.00	25	Liu, et al	10.1016/j.cell.2021.06.020
Patient	A	B.1.617.2	Delta	Live Virus FRNT50	1.42	2.67	34	Liu, et al	10.1016/j.cell.2021.06.020
BioNTech	A	B.1.617.2	Delta	Live Virus FRNT50	1.32	2.50	25	Liu, et al	10.1016/j.cell.2021.06.020
AstraZeneca	A	B.1.617.2	Delta	Live Virus FRNT50	2.11	4.31	25	Liu, et al	10.1016/j.cell.2021.06.020
Patient	A	P.1	Gamma	Live Virus FRNT50	1.65	3.13	34	Liu, et al	10.1016/j.cell.2021.06.020
Patient	P.1	A	Gamma	Live Virus FRNT50	1.96	3.89	17	Liu, et al	10.1016/j.cell.2021.06.020
BioNTech	A	P.1	Gamma	Live Virus FRNT50	1.39	2.62	25	Liu, et al	10.1016/j.cell.2021.06.020
AstraZeneca	A	P.1	Gamma	Live Virus FRNT50	1.52	2.86	25	Liu, et al	10.1016/j.cell.2021.06.020
Patient	A	B.1.617.1-C	Kappa	Pseudovirus FRNT50	0.58	1.49	34	Liu, et al	10.1016/j.cell.2021.06.020
Patient	A	B.1.617.1-A	Kappa	Pseudovirus FRNT50	1.33	2.52	34	Liu, et al	10.1016/j.cell.2021.06.020
Patient	A	B.1.617.1-B	Kappa	Pseudovirus FRNT50	1.97	3.91	34	Liu, et al	10.1016/j.cell.2021.06.020
BioNTech	A	B.1.617.1-B	Kappa	Pseudovirus NT50	1.45	2.72	25	Liu, et al	10.1016/j.cell.2021.06.020
AstraZeneca	A	B.1.617.1-B	Kappa	Pseudovirus NT50	1.39	2.63	25	Liu, et al	10.1016/j.cell.2021.06.020
Patient	B.1.351	P.1	Non-VOC	Live Virus FRNT50	1.00	2.00	14	Liu, et al	10.1016/j.cell.2021.06.020
Patient	P.1	B.1.351	Non-VOC	Live Virus FRNT50	1.16	2.24	17	Liu, et al	10.1016/j.cell.2021.06.020
Patient	B.1.1.7	B.1.617.1-B	Non-VOC	Pseudovirus NT50	2.09	4.27	34	Liu, et al	10.1016/j.cell.2021.06.020
Patient	B.1.1.7	B.1.351	Non-VOC	Live Virus FRNT50	1.38	2.60	18	Liu, et al	10.1016/j.cell.2021.06.020
Patient	B.1.351	B.1.1.7	Non-VOC	Live Virus FRNT50	1.72	3.30	14	Liu, et al	10.1016/j.cell.2021.06.020
Patient	B.1.1.7	P.1	Non-VOC	Live Virus FRNT50	0.76	1.69	18	Liu, et al	10.1016/j.cell.2021.06.020
Patient	P.1	B.1.1.7	Non-VOC	Live Virus FRNT50	2.03	4.09	17	Liu, et al	10.1016/j.cell.2021.06.020
Patient	B.1.1.7	B.1.617.2	Non-VOC	Live Virus FRNT50	0.60	1.51	18	Liu, et al	10.1016/j.cell.2021.06.020
Patient	B.1.351	B.1.617.2	Non-VOC	Live Virus FRNT50	3.53	11.54	14	Liu, et al	10.1016/j.cell.2021.06.020
Patient	P.1	B.1.617.2	Non-VOC	Live Virus FRNT50	3.51	11.37	17	Liu, et al	10.1016/j.cell.2021.06.020
AstraZeneca	B.1(D614G)	B.1.351	Beta	Pseudovirus ID50	2.00	4.01	13	Madhi SA, et al	10.1056/NEJMoa2102214
Patient	B.1.1.117	B.1.351	Beta	Pseudovirus ID50	2.19	4.58	6	Madhi SA, et al	10.1056/NEJMoa2102214
AstraZeneca	B.1.1	B.1.351	Beta	Live Virus FRNT50	3.46	11.00	13	Madhi SA, et al	10.1056/NEJMoa2102214
Patient	B.1.1.117	B.1.351	Beta	Live Virus FRNT50	2.95	7.75	6	Madhi SA, et al	10.1056/NEJMoa2102214
AstraZeneca	B.1(D614G)	K417N/E484K/N5	Non-VOC	Pseudovirus ID50	1.80	3.49	13	Madhi SA, et al	10.1056/NEJMoa2102214
Patient	B.1.1.117	K417N/E484K/N5	Non-VOC	Pseudovirus ID50	1.72	3.30	6	Madhi SA, et al	10.1056/NEJMoa2102214
Patient	B.1.351 v2	B.1(D614G)	Non-VOC	Pseudovirus ID50	1.61	3.04	57	Moyo-Gwete, et al	10.1056/NEJMc2104192
BioNTech	A	B.1.1.7	Alpha	Pseudovirus NT50	-0.30	0.81	15	Schmidt F,et al	10.1038/s41586-021-04005-0

BioNTech	A	B.1.351.3	Beta	Pseudovirus NT50	1.69	3.23	15	Schmidt F,et al	10.1038/s41586-021-04005-0
BioNTech	A	B.1.617.2	Delta	Pseudovirus NT50	2.25	4.76	15	Schmidt F,et al	10.1038/s41586-021-04005-0
BioNTech	A	P.1	Gamma	Pseudovirus NT50	0.79	1.72	15	Schmidt F,et al	10.1038/s41586-021-04005-0
BioNTech	A	B.1.526	Iota	Pseudovirus NT50	0.71	1.64	15	Schmidt F,et al	10.1038/s41586-021-04005-0
BioNTech	A	WIV1-CoV	WIV1-CoV	Pseudovirus NT50	4.06	16.67	15	Schmidt F,et al	10.1038/s41586-021-04005-0
BioNTech	A	SARS-CoV	SARS	Pseudovirus NT50	5.06	33.33	15	Schmidt F,et al	10.1038/s41586-021-04005-0
Patient	B.1(D614G)	B.1.351	Beta	Pseudovirus ID50	3.71	13.10	14	Shen X-Y, et al	10.1056/NEJMc2103740
Moderna	B.1(D614G)	B.1.351	Beta	Pseudovirus ID50	3.28	9.70	26	Shen X-Y, et al	10.1056/NEJMc2103740
Novavax	B.1(D614G)	B.1.351	Beta	Pseudovirus ID50	3.86	14.50	23	Shen X-Y, et al	10.1056/NEJMc2103740
Patient	B.1(D614G)	B.1.429	Epsilon	Pseudovirus ID50	1.63	3.10	14	Shen X-Y, et al	10.1056/NEJMc2103740
Moderna	B.1(D614G)	B.1.429	Epsilon	Pseudovirus ID50	1.00	2.00	26	Shen X-Y, et al	10.1056/NEJMc2103740
Novavax	B.1(D614G)	B.1.429	Epsilon	Pseudovirus ID50	1.32	2.50	23	Shen X-Y, et al	10.1056/NEJMc2103740
Patient	A	B.1.1.7	Alpha	Live Virus FRNT50	1.54	2.90	34	Supasa, et al	10.1016/j.cell.2021.02.033
AstraZeneca	A	B.1.1.7	Alpha	Live Virus FRNT50	1.32	2.50	15	Supasa, et al	10.1016/j.cell.2021.02.033
AstraZeneca	A	B.1.1.7	Alpha	Live Virus FRNT50	1.07	2.10	10	Supasa, et al	10.1016/j.cell.2021.02.033
BioNTech	A	B.1.1.7	Alpha	Live Virus FRNT50	1.72	3.30	25	Supasa, et al	10.1016/j.cell.2021.02.033
Patient	B.1.1.7	A	Alpha	Live Virus FRNT50	-0.12	0.92	13	Supasa, et al	10.1016/j.cell.2021.02.033
Patient	A	B.1.617.2	Delta	Live Virus FRNT50	1.26	2.40	24	Suthar MS, et al	10.1056/NEJMc2107799
Moderna	A	B.1.617.2	Delta	Live Virus FRNT50	1.58	3.00	15	Suthar MS, et al	10.1056/NEJMc2107799
BioNTech	A	B.1.617.2	Delta	Live Virus FRNT50	1.72	3.30	10	Suthar MS, et al	10.1056/NEJMc2107799
Patient	A	B.1.617.1	Kappa	Live Virus FRNT50	2.70	6.50	24	Suthar MS, et al	10.1056/NEJMc2107799
Moderna	A	B.1.617.1	Kappa	Live Virus FRNT50	2.81	7.00	15	Suthar MS, et al	10.1056/NEJMc2107799
BioNTech	A	B.1.617.1	Kappa	Live Virus FRNT50	2.81	7.00	10	Suthar MS, et al	10.1056/NEJMc2107799
Patient	B.1(D614G)	B.1.351	Beta	Pseudovirus IC50	2.29	4.90	8	Tada, et al	10.1101/2021.07.02.450959
BioNTech	B.1(D614G)	B.1.351	Beta	Pseudovirus IC50	1.32	2.50	15	Tada, et al	10.1101/2021.07.02.450959
Moderna	B.1(D614G)	B.1.351	Beta	Pseudovirus IC50	2.00	4.00	6	Tada, et al	10.1101/2021.07.02.450959
Patient	B.1(D614G)	C.37	Lambda	Pseudovirus IC50	1.72	3.30	8	Tada, et al	10.1101/2021.07.02.450959
BioNTech	B.1(D614G)	C.37	Lambda	Pseudovirus IC50	1.58	3.00	15	Tada, et al	10.1101/2021.07.02.450959
Moderna	B.1(D614G)	C.37	Lambda	Pseudovirus IC50	1.20	2.30	6	Tada, et al	10.1101/2021.07.02.450959
SARS Patient	SARS-CoV	B.1.1.7	SARS	Pseudovirus NT50	3.62	12.33	10	Tan C-W, et al	10.1056/NEJMoa2108453
Patient	B.1.1.7	SARS-CoV	SARS	Pseudovirus NT50	4.47	22.16	10	Tan C-W, et al	10.1056/NEJMoa2108453
BioNTech	B.1.1.7	SARS-CoV	SARS	Pseudovirus NT50	4.02	16.18	10	Tan C-W, et al	10.1056/NEJMoa2108453
BioNTech	A	B.1.1.7	Alpha	Live Virus IC50	1.38	2.60	159	Wall EC, et al	10.1016/S0140-6736(21)01290-3

BioNTech	A	B.1.351	Beta	Live Virus IC50	2.29	4.90	159	Wall EC, et al	10.1016/S0140-6736(21)01290-3
BioNTech	A	B.1.617.2	Delta	Live Virus IC50	2.54	5.80	159	Wall EC, et al	10.1016/S0140-6736(21)01290-3
BioNTech	A	B.1(D614G)	Non-VOC	Live Virus IC50	1.20	2.30	159	Wall EC, et al	10.1016/S0140-6736(21)01290-3
Patient	A	B.1.1.7	Alpha	Pseudovirus ID50	1.68	3.20	20	Wang, et al	10.1038/s41586-021-03398-2
Moderna	A	B.1.1.7	Alpha	Pseudovirus ID50	0.85	1.80	12	Wang, et al	10.1038/s41586-021-03398-2
BioNTech	A	B.1.1.7	Alpha	Pseudovirus ID50	1.00	2.00	10	Wang, et al	10.1038/s41586-021-03398-2
Patient	A	B.1.351	Beta	Pseudovirus ID50	4.46	22.00	20	Wang, et al	10.1038/s41586-021-03398-2
Moderna	A	B.1.351	Beta	Pseudovirus ID50	3.10	8.60	12	Wang, et al	10.1038/s41586-021-03398-2
BioNTech	A	B.1.351	Beta	Pseudovirus ID50	2.70	6.50	10	Wang, et al	10.1038/s41586-021-03398-2
Patient	B.1(D614G)	B.1.351	Beta	Pseudovirus ID50	2.51	5.69	44	Wibmer, et al	10.1038/s41591-021-01285-x
Patient	B.1(D614G)	K417N/E484K/N5	Non-VOC	Pseudovirus ID50	2.14	4.42	44	Wibmer, et al	10.1038/s41591-021-01285-x
BioNTech	A	B.1.351	Beta	Live Virus FRNT50	2.92	7.57	25	Zhou, et al	10.1016/j.cell.2021.02.037
AstraZeneca	A	B.1.351	Beta	Live Virus FRNT50	3.17	9.00	25	Zhou, et al	10.1016/j.cell.2021.02.037
Patient	A	B.1.351	Beta	Live Virus FRNT50	3.72	13.19	34	Zhou, et al	10.1016/j.cell.2021.02.037
Patient	B.1.1.7	B.1.351	Beta	Live Virus FRNT50	1.65	3.13	14	Zhou, et al	10.1016/j.cell.2021.02.037

(b) Omicron Testing Set

Serum	VirusRef	VirusVar	WHO	Type	log2FC	Mean	N	Source	DOI
Patient	B.1(D614G)	B.1.1.529	Omicron	Pseudovirus NT50	9.26	614.00	17	Hoffmann M, et al	10.1016/j.cell.2021.12.032
BioNTech	B.1(D614G)	B.1.1.529	Omicron	Pseudovirus NT50	6.07	67.11	11	Hoffmann M, et al	10.1016/j.cell.2021.12.032
AstraZeneca/ BioNTech	B.1(D614G)	B.1.1.529	Omicron	Pseudovirus NT50	3.69	12.89	10	Hoffmann M, et al	10.1016/j.cell.2021.12.032
Moderna	A	B.1.1.529	Omicron	Pseudovirus GMNT	5.42	42.80	33	Garcia-Beltran2, et al	10.1016/j.cell.2021.12.033
BioNTech	A	B.1.1.529	Omicron	Pseudovirus GMNT	6.87	117.20	30	Garcia-Beltran2, et al	10.1016/j.cell.2021.12.033
AstraZeneca	A	B.1.1.529	Omicron	Pseudovirus GMNT	3.70	13.00	8	Garcia-Beltran2, et al	10.1016/j.cell.2021.12.033
Patient	A	B.1.1.529	Omicron	Live Virus FRNT50	3.45	10.90	32	Dejnirattisai, et al	10.1016/j.cell.2021.12.046
BioNTech	A	B.1.1.529	Omicron	Live Virus FRNT50	6.62	98.10	41	Dejnirattisai, et al	10.1016/j.cell.2021.12.046
AstraZeneca	A	B.1.1.529	Omicron	Live Virus FRNT50	4.36	20.60	20	Dejnirattisai, et al	10.1016/j.cell.2021.12.046
Moderna	A	B.1.1.530	Omicron	Pseudovirus NT50	4.05	16.60	34	Cameroni, et al	10.1038/s41586-021-04386-2
BioNTech	A	B.1.1.531	Omicron	Pseudovirus NT50	4.04	16.40	41	Cameroni, et al	10.1038/s41586-021-04386-2
AstraZeneca	A	B.1.1.532	Omicron	Pseudovirus NT50	5.14	35.30	11	Cameroni, et al	10.1038/s41586-021-04386-2
Sputnik V	A	B.1.1.533	Omicron	Pseudovirus NT50	3.77	13.60	10	Cameroni, et al	10.1038/s41586-021-04386-2
Sinopharm	A	B.1.1.534	Omicron	Pseudovirus NT50	3.75	13.50	13	Cameroni, et al	10.1038/s41586-021-04386-2

Patient	A	B.1.1.535	Omicron	Pseudovirus NT50	5.52	46.00	24	Cameroni, et al	10.1038/s41586-021-04386-2
AstraZeneca	A	B.1.1.536	Omicron	Pseudovirus NT50	3.56	11.80	12	Liu2, et al	10.1038/s41586-021-04388-0
BioNTech	A	B.1.1.537	Omicron	Pseudovirus NT50	4.47	22.20	12	Liu2, et al	10.1038/s41586-021-04388-0
Moderna	A	B.1.1.538	Omicron	Pseudovirus NT50	3.07	8.40	12	Liu2, et al	10.1038/s41586-021-04388-0
BioNTech	A	B.1.1.539	Omicron	Live Virus FRNT50	2.49	5.60	10	Liu2, et al	10.1038/s41586-021-04388-0
Moderna	A	B.1.1.540	Omicron	Live Virus FRNT50	3.12	8.70	7	Liu2, et al	10.1038/s41586-021-04388-0
Patient	A	B.1.1.541	Omicron	Pseudovirus NT50	5.01	32.20	10	Liu2, et al	10.1038/s41586-021-04388-0
BioNTech	A	B.1.1.542	Omicron	Pseudovirus NT50	6.09	68.30	30	Gruell, et al	10.1038/s41591-021-01676-0
