# Supplementary material for: Computation of Antigenicity Predicts SARS-CoV-2 Vaccine Breakthrough Variants
Source: Front Immunol. 2022 Mar 24;13:861050. doi: 10.3389/fimmu.2022.861050 (PMC8987580; doi:10.3389/fimmu.2022.861050)
Supplement: Supplementary file 4 [file Table_3.pdf]

Table S3. SARS-CoV-2 variants analysed in our study. We gratefully acknowledge the following Authors from the Originating laboratories responsible for obtaining the specimens and the Submitting laboratories where genetic sequence data were generated and shared via the GISAID Initiative, on which this research is based.

Lineage	Accession ID	Virus Name	Collection Date	Submitting Lab	Authors
A	EPI_ISL_529213	Wuhan/IME-WH01/2019	2019/12/30	Beijing Institute of Microbiology and Epidemiology	Fan et al
A.1	EPI_ISL_2836011	USA/un-Yale-5643/2020	2020/1/19	Epidemiology of Microbial Diseases, Yale School of Public Health	Vogels et al
A.11	EPI_ISL_420078	Senegal/620/2020	2020/3/20	Institut Pasteur de Dakar	Ndongo Dia et al
A.12	EPI_ISL_512816	SierraLeone/KGH-G-8625/2020	2020/4/10	Kenema Government Hospital, Ministry of Health and Sanitation	Goba et al
A.15	EPI_ISL_622356	Denmark/DCGC-6742/2020	2020/4/20	Albertsen lab, Department of Chemistry and Bioscience, Aalborg University, Denmark	Danish Covid-19 Genome Consortia et al
A.16	EPI_ISL_480042	Japan/PG-0372/2020	2020/2/15	Pathogen Genomics Center, National Institute of Infectious Diseases	Tsuyoshi Sekizuka et al
A.17	EPI_ISL_660334	France/ARA-SC338/2020	2020/3/2	CNR Virus des Infections Respiratoires - France SUD	Antonin Bal et al
A.18	EPI_ISL_1662585	Coted-Ivoire/BKE0714-bc14/2020	2020/7/24	Project group Epidemology of Highly Pathogenic Microorganisms, Robert Koch-Institute	Chantal Akoua-Koffi et al
A.19	EPI_ISL_614351	Coted-Ivoire/BKE0041/2020	2020/6/4	Project group Epidemology of Highly Pathogenic Microorganisms, Robert Koch-Institute	Chantal Akoua-Koffi et al
A.2	EPI_ISL_496607	Panama/328719/2020	2020/2/22	Gorgas Memorial Laboratory of Health Studies	Danilo Franco et al
A.2.2	EPI_ISL_483708	Israel/n14268/2020	2020/3/17	Israel Central Virology laboratory	Neta Zuckerman et al
A.2.3	EPI_ISL_425752	Scotland/CVR24/2020	2020/3/12	COVID-19 Genomics UK (COG-UK) Consortium	Ana da Silva Filipe et al
A.2.4	EPI_ISL_1225382	Panama/GMI-PA352445/2020	2020/2/4	Gorgas Memorial Laboratory of Health Studies	Yamilka Diaz et al
A.2.5	EPI_ISL_1502840	Panama/GMI-PA341357/2020	2020/4/15	Gorgas Memorial Laboratory of Health Studies	Gonzalez Claudia et al
A.2.5	EPI_ISL_2000585	USA/RI_RKL_001_31745_AM614/2020	2020/4/25	Rami Kantor lab	Josephine Darpolor et al

A.2.5.1	EPI_ISL_1502839	Panama/GMI-PA340863/2020	2020/4/14	Gorgas Memorial Laboratory of Health Studies	Gonzalez Claudia et al
A.2.5.2	EPI_ISL_1502836	Panama/GMI-PA339770/2020	2020/4/12	Gorgas Memorial Laboratory of Health Studies	Gonzalez Claudia et al
A.2.5.3	EPI_ISL_1681051	USA/FL-CDC-LC0040585/2021	2021/4/7	Centers for Disease Control and Prevention Division of Viral Diseases, Pathogen Discovery	Dakota Howard et al
A.21	EPI_ISL_487456	Mali/M002675/2020	2020/4/10	Bundeswehr Institut of Microbiology	Kouriba et al
A.22	EPI_ISL_457938	Oman/RESP-20-1976/2020	2020/3/15	Oman-NIC	Samira Al-Maruqi et al
A.23	EPI_ISL_738016	Uganda/UG109/2020	2020/8/14	MRC/UVRI & LSHTM Uganda Research Unit	Matthew Cotten et al
A.23.1	EPI_ISL_5143058	UnitedArabEmirates/BTC-4974/2020	2020/9/21	BTC, Khalifa University	Habiba Al Safar et al
A.24	EPI_ISL_475586	USA/CA-CSMC110/2020	2020/3/22	Cedars-Sinai Medical Center, Molecular Pathology Laboratory of Department of Pathology & Laboratory Medicine and Genomic Core	Wenjuan Zhang et al
A.25	EPI_ISL_2602919	Kenya/K10131/2020	2020/6/8	KEMRI-Wellcome Trust Research Programme,Kilifi	Githinji G. et al
A.26	EPI_ISL_539009	England/LEED-2A89EF/2020	2020/3/31	Wellcome Sanger Institute for the COVID-19 Genomics UK (COG-UK) consortium	Louissa Macfarlane-Smith et al
A.27	EPI_ISL_757470	Denmark/DCGC-22492/2020	2020/12/14	Albertsen Lab, Department of Chemistry and Bioscience, Aalborg University, Denmark	Danish Covid-19 Genome Consortium et al
A.28	EPI_ISL_812821	Egypt/CCHE57357-A-48/2020	2020/7/28	Genomics Program, Children Cancer Hospital	Hatem et al
A.29	EPI_ISL_1731548	Gambia/22178/2020	2020/8/29	MRCG at LSHTM Genomics lab	Abdul Karim sesay et al
A.3	EPI_ISL_438234	USA/MD-HP00076/2020	2020/3/11	Johns Hopkins Hospital Department of Pathology	Peter M. Thielen et al
A.4	EPI_ISL_417514	USA/WI-UW-22/2020	2020/3/13	University of Wisconsin-Madison AIDS Vaccine Research Laboratories	Gage Moreno et al
A.5	EPI_ISL_529992	Spain/MD-H12-61/2020	2020/3/1	Hospital Universitario 12 de Octubre	Sara González et al
A.6	EPI_ISL_437612	Thailand/SI203285-NT/2020	2020/1/23	Faculty of Medicine	Rodpan et al
A.7	EPI_ISL_455749	India/OR-RMRC103/2020	2020/5/6	Immunogenomics lab, Institute of Life Sciences, Bhubaneswar	Sunil Raghav et al

A.9	EPI_ISL_775626	Germany/HH-hpi-p2134/2020	2020/3/21	Heinrich Pette Institute, Leibniz Institute for Experimental Virology	Alexis Robitaille et al
AA.1	EPI_ISL_540903	Wales/PHWC-16A540/2020	2020/9/6	COVID-19 Genomics UK (COG-UK) Consortium	Catherine Moore et al
AA.2	EPI_ISL_680608	Wales/PHWC-48D32C/2020	2020/11/14	COVID-19 Genomics UK (COG-UK) Consortium	Catherine Moore et al
AA.3	EPI_ISL_628267	Wales/PHWC-485C38/2020	2020/10/21	COVID-19 Genomics UK (COG-UK) Consortium	Catherine Moore et al
AA.4	EPI_ISL_634761	Wales/ALDP-AA308D/2020	2020/10/22	Wellcome Sanger Institute for the COVID-19 Genomics UK (COG-UK) consortium	Jacquelyn Wynn et al
AA.5	EPI_ISL_742926	Wales/PHWC-49DECE/2020	2020/12/5	COVID-19 Genomics UK (COG-UK) Consortium	Catherine Moore et al
AA.6	EPI_ISL_637606	Wales/PHWC-48738C/2020	2020/10/13	COVID-19 Genomics UK (COG-UK) Consortium	Catherine Moore et al
AA.7	EPI_ISL_666232	Wales/PHWC-48B3D3/2020	2020/11/4	COVID-19 Genomics UK (COG-UK) Consortium	Catherine Moore et al
AA.8	EPI_ISL_814416	Wales/PHWC-4A6F92/2020	2020/11/25	COVID-19 Genomics UK (COG-UK) Consortium	Catherine Moore et al
AB.1	EPI_ISL_748906	Denmark/DCGC-19364/2020	2020/12/7	Albertsen Lab, Department of Chemistry and Bioscience, Aalborg University, Denmark	Danish Covid-19 Genome Consortium et al
AC.1	EPI_ISL_629268	England/MILK-ACC112/2020	2020/10/21	Wellcome Sanger Institute for the COVID-19 Genomics UK (COG-UK) consortium	The Lighthouse Lab in Milton Keynes et al
AD.1	EPI_ISL_669913	Denmark/DCGC-10343/2020	2020/11/2	Albertsen Lab, Department of Chemistry and Bioscience, Aalborg University, Denmark	Danish Covid-19 Genome Consortium et al
AD.2	EPI_ISL_573313	England/NORT-286167/2020	2020/7/21	COVID-19 Genomics UK (COG-UK) Consortium	Darren L Smith et al
AD.2.1	EPI_ISL_933914	Lithuania/ID2614/2020	2020/12/4	Vilnius university hospital Santaros Klinikos, Center of Laboratory Medicine	Ingrida Olendraite et al
AE.1	EPI_ISL_2982533	DRC/7325/2020	2020/5/2	Pathogen Sequencing Lab, National Institute for Biomedical Research (INRB)	Placide Mbala-Kingebeni et al
AE.2	EPI_ISL_486887	Bahrain/02/2020	2020/6/23	National Influenza Center, Bahrain	Zaed et al
AE.3	EPI_ISL_977373	Zambia/ZMB-24445/2020	2020/6/4	UNZAVET and PATH	Mulenga Mwenda-Chimfwembe et al
AE.4	EPI_ISL_486889	Bahrain/04/2020	2020/6/23	National Influenza Center, Bahrain	Altaif et al

AE.5	EPI_ISL_977392	Zambia/ZMB-31145/2020	2020/6/28	UNZAVET and PATH	Mulenga Mwenda-Chimfwembe et al
AE.6	EPI_ISL_682307	Bahrain/920453170/2020	2020/10/11	Communicable Disease Laboratory, Public Health Directorate	Alwasti et al
AE.7	EPI_ISL_681301	Bahrain/920396988_S5_L001/2020	2020/9/11	Communicable Disease Laboratory, Public Health Directorate	Alwasti et al
AE.8	EPI_ISL_2163060	Canada/ABPHL-02509/2020	2020/8/5	Public Health Agency of Canada (PHAC) National Microbiology Laboratory	Buss et al
AF.1	EPI_ISL_1496474	Switzerland/BL-ETHZ-330007/2020	2020/10/16	Department of Biosystems Science and Engineering, ETH Zürich	Christian Beisel et al
AG.1	EPI_ISL_2886646	Slovenia/91111/2020	2020/9/11	Institute of Microbiology and Immunology, Faculty of Medicine, University of Ljubljana	Alen Suljič et al
AH.1	EPI_ISL_1059585	Switzerland/BE-ETHZ-481450/2021	2021/1/5	Department of Biosystems Science and Engineering, ETH Zürich	Chaoran Chen et al
AH.2	EPI_ISL_569103	France/PAC-IHU-1270/2020	2020/8/18	MEPHI, Aix Marseille University	Anthony LEVASSEUR et al
AH.3	EPI_ISL_759771	Finland/23LS1E6/2020	2020/10/23	Department of Virology, Faculty of Medicine, University of Helsinki, Helsinki, Finland	Teemu Smura et al
AJ.1	EPI_ISL_732022	Portugal/PT1795/2020	2020/11/8	Instituto Nacional de Saude (INSA)	Borges et al
AK.1	EPI_ISL_590451	England/QEUH-9B5511/2020	2020/9/3	Wellcome Sanger Institute for the COVID-19 Genomics UK (COG-UK) consortium	Harper VanSteenhouse et al
AK.2	EPI_ISL_631381	Germany/NW-HHU-248/2020	2020/9/19	Center of Medical Microbiology, Virology, and Hospital Hygiene, University of Duesseldorf	Maximilian Damagnez et al
AL.1	EPI_ISL_1202078	USA/TX-HMH-MCoV-12043/2020	2020/9/18	Houston Methodist Hospital	S. Wesley Long et al
AM.1	EPI_ISL_731937	Portugal/PT1799/2020	2020/11/9	Instituto Nacional de Saude (INSA)	Borges et al
AM.3	EPI_ISL_665814	England/LOND-1250DAB/2020	2020/11/10	COVID-19 Genomics UK (COG-UK) Consortium	Judith Heaney et al
AM.4	EPI_ISL_1739607	Canada/ON-SC2233/2020	2020/11/5	McMaster University	Allison McGeer et al
AN.1	EPI_ISL_548256	Sweden/20-52355/2020	2020/7/7	The Public Health Agency of Sweden	Anna-Malin Linde et al
AQ.1	EPI_ISL_757317	Finland/17MR7cE12/2020	2020/11/17	Department of Virology, Faculty of Medicine, University of Helsinki, Helsinki, Finland	Teemu Smura et al

AQ.2	EPI_ISL_616717	Denmark/DCGC-2956/2020	2020/8/24	Albertsen lab, Department of Chemistry and Bioscience, Aalborg University, Denmark	Danish Covid-19 Genome Consortia et al
AS.1	EPI_ISL_1403144	Canada/Qc-HSE-5524134965/2020	2020/10/6	Laboratoire de santé publique du Québec	Sandrine Moreira et al
AS.2	EPI_ISL_2900306	England/ALDP-9E809C/2020	2020/9/24	COVID-19 Genomics UK (COG-UK) Consortium	PHE Covid Sequencing Team et al
AU.1	EPI_ISL_1424680	PapuaNewGuinea/PNG293/2021	2021/1/1	Melbourne Diagnostic Unit Public Health Laboratory (MDU-PHL)	Palou et al
AU.2	EPI_ISL_2379985	Malaysia/UNIMAS-B187/2021	2021/1/3	Institute of Health and Community Medicine	Chan Chia Jui et al
AU.3	EPI_ISL_1118214	Indonesia/JK-NIHRD-MI-21-01773/2021	2021/2/9	National Institute of Health Research and Development	Hana Apsari Pawestri et al
AV.1	EPI_ISL_1386007	Turkey/HSGM-2150/2021	2021/1/27	Ministry of Health Turkey	Fatma Bayrakdar et al
AW.1	EPI_ISL_974269	Canada/BC-BCCDC-5890/2020	2020/10/12	BCCDC Public Health Laboratory	Prystajecky Natalie et al
AY.1	EPI_ISL_1838677	India/AP-CCMB-BN992/2021	2021/4/7	CSIR-Centre for Cellular and Molecular Biology-INSACOG	Payel Mukherjee et al
AY.100	EPI_ISL_2937904	Mexico/AGU-InDRE_FB18599_S4467/2020	2020/9/22	Instituto de Diagnostico y Referencia Epidemiologicos (INDRE)	Claudia Wong-Arambula et al
AY.102	EPI_ISL_4077684	Switzerland/BE-IFIK-210906_os-89/2021	2021/1/8	Institute for Infectious Diseases	Stefan Neuenschwander et al
AY.102	EPI_ISL_2716761	Italy/LAZ-IFO-05103208/2021	2021/5/7	IRCCS Regina Elena National Cancer Institute	Ludovica Ciuffreda et al
AY.102	EPI_ISL_4415500	India/MH-IISER-Pune-IP01472/2021	2021/1/9	IISER Pune-INSACOG	Krishanpal Karmodiya et al
AY.102	EPI_ISL_4415528	India/MH-IISER-Pune-IP-01014/2021	2021/2/9	IISER Pune-INSACOG	Krishanpal Karmodiya et al
AY.102	EPI_ISL_4101601	Turkey/HSGM-E2913/2021	2021/3/9	Ministry of Health Turkey	Fatma Bayrakdar et al
AY.102	EPI_ISL_4415524	India/MH-IISER-Pune-IP01463/2021	2021/1/9	IISER Pune-INSACOG	Krishanpal Karmodiya et al
AY.103	EPI_ISL_5886224	India/PY-nCOVb30339_S752_R1_001/2021	2021/1/1	inStem NCBS – INSACOG	Uma Ramakrishnan et al
AY.103	EPI_ISL_4884869	Italy/SIC_CQRC_21066374/2021	2021/3/16	CQRC_QUALITY CONTROL CHEMICAL BIOLOGICAL RISK_AOOR Villa Sofia Cervello Palermo	Di Gaudio F. et al
AY.106	EPI_ISL_4101782	Turkey/HSGM-E3132/2021	2021/3/9	Ministry of Health Turkey	Fatma Bayrakdar et al

AY.107	EPI_ISL_2379462	India/TN-C_100_S15_R1_001/2021	2021/2/5	inStem NCBS - INSACOG	Uma Ramakrishnan Dasaradhi Palakodeti Aswin SaiNarain et al
AY.11	EPI_ISL_1790701	England/MILK-1523694/2021	2021/4/20	Wellcome Sanger Institute for the COVID-19 Genomics UK (COG-UK) Consortium	The Lighthouse Lab in Milton Keynes et al
AY.110	EPI_ISL_1907995	USA/MI-CDC-STM-000056311/2021	2021/4/14	Centers for Disease Control and Prevention Division of Viral Diseases, Pathogen Discovery	Dakota Howard et al
AY.111	EPI_ISL_1652897	England/RAND-14FD37F/2021	2021/4/12	Wellcome Sanger Institute for the COVID-19 Genomics UK (COG-UK) Consortium	Randox Laboratories et al
AY.114	EPI_ISL_2774897	India/TG-CCMB-CIA4758/2021	2021/4/10	CSIR-Centre for Cellular and Molecular Biology-INSACOG	Lamuk Zaveri et al
AY.115	EPI_ISL_5502108	Botswana/R36B65_BHP_000640473/2021	2021/5/31	Botswana Harvard HIV Reference Laboratory	Sikhulile Moyo et al
AY.116	EPI_ISL_6347359	Norway/24584/2021	2021/1/21	Norwegian Institute of Public Health, Department of Virology	Kathrine Stene-Johansen et al
AY.116.1	EPI_ISL_2545604	Belgium/UZA-UA-CV2184230236/2021	2021/6/7	Labo Klinische Biologie, UZA	Jasmine Coppens et al
AY.118	EPI_ISL_4484419	Slovakia/UVZ_PL36_B6_17813/2021	2021/2/9	Laboratory of Genomics and Bioinformatics, Comenius University Science Park	Tatiana Sedláčková et al
AY.120.1	EPI_ISL_5348704	USA/ID-IBL-760105/2021	2021/2/23	Idaho Bureau of Laboratories	R. Beukelman et al
AY.120.2	EPI_ISL_2227435	USA/MD-MDH-2355/2021	2021/5/6	MD PHL	Maryland Department of Health Laboratories Administration et al
AY.120.2.1	EPI_ISL_2762159	Germany/BW-RKI-I-183727/2021	2021/6/6	Robert Koch Institute	?
AY.122	EPI_ISL_1663563	India/ILSGS00993/2021	2021/3/25	Institute of Life Sciences - INSACOG	Sunil K. Raghav et al
AY.123	EPI_ISL_5414903	Belgium/rega-12446/2021	2021/4/25	KU Leuven, Rega Institute, Clinical and Epidemiological Virology	Tony Wawina-Bokalanga et al
AY.125	EPI_ISL_5508422	Israel/CVL-22316/2021	2021/1/8	Israel Central Virology laboratory	Neta Zuckerman et al
AY.125	EPI_ISL_4271105	Spain/CT-LabRefCat-751420/2021	2021/1/29	Laboratori de Referencia de Catalunya	Ramirez A. et al
AY.13	EPI_ISL_3215995	Mexico/BCN-SEARCH-104131/2021	2021/2/4	Andersen lab at Scripps Research	SEARCH Alliance San Diego with Jonathan Gonzalez Garcia et al

AY.14	EPI_ISL_5778142	USA/KY-LMDC-20180452/2021	2021/3/30	University of Louisville Sequencing Technology Center	Elizabeth Hudson et al
AY.16	EPI_ISL_4415438	India/MH-IISER-Pune-IP-01040/2021	2021/2/9	IISER Pune-INSACOG	Krishanpal Karmodiya et al
AY.18	EPI_ISL_3484616	Canada/BC-BCCDC-93175/2021	2021/5/11	BCCDC Public Health Laboratory	Prystajecky Natalie et al
AY.19	EPI_ISL_2897754	India/MH-NS2079/2021	2021/4/19	CDFD	Murali Bashyam et al
AY.2	EPI_ISL_3275127	USA/ID-UNM-IBL720784/2021	2021/3/31	Center for Global Health, University of New Mexico Health Sciences Center	Daryl Domman et al
AY.20	EPI_ISL_5703214	USA/TN-SPHL-0539/2021	2021/1/14	TN DOH Lab Services	Karen Beasley-Maynard et al
AY.20	EPI_ISL_5639223	USA/CA-CDPH-500008629/2021	2021/8/2	California Department of Public Health	Emily Smith on behalf of CDPH-COVIDNet et al
AY.22	EPI_ISL_2536066	Portugal/PT8419/2021	2021/5/28	Instituto Nacional de Saude (INSA)	Borges et al
AY.23	EPI_ISL_5395771	Indonesia/JA-GS-EIJK-RSRM-0172/2020	2020/7/29	Eijkman Research Center for Molecular Biology, National Research and Innovation Agency; Raden Mattaher Regional General Hospital (RSUD)	Jessica R Saragih et al
AY.23.1	EPI_ISL_3427022	Singapore/3563/2021	2021/8/7	National Public Health Laboratory, National Centre for Infectious Diseases	Katherine Ching et al
AY.24	EPI_ISL_5395769	Indonesia/JA-GS-EIJK-RSRM-0170/2020	2020/7/29	Eijkman Research Center for Molecular Biology, National Research and Innovation Agency; Raden Mattaher Regional General Hospital (RSUD)	Willy Agustine et al
AY.25	EPI_ISL_4689452	USA/WV-WVU-WV121217/2020	2020/7/21	WVU and Marshall University Combined Genomics Core Facilities	James Denvir et al
AY.26	EPI_ISL_4484170	Slovakia/UVZ_PL36_C5_17806/2021	2021/1/9	Laboratory of Genomics and Bioinformatics, Comenius University Science Park	Tatiana Sedláčková et al
AY.28	EPI_ISL_4469231	SriLanka/idea_uoc_00065/2021	2021/2/16	IDEA Laboratory	Inoka C. Perera et al
AY.29.1	EPI_ISL_4723864	Japan/PG-119190/2021	2021/7/2	Pathogen Genomics Center, National Institute of Infectious Diseases	Tsuyoshi Sekizuka et al
AY.3	EPI_ISL_4558284	USA/WY-WYPHL-21070660/2020	2020/9/2	Wyoming Public Health Laboratory	Jim Mildenberger et al
AY.3.1	EPI_ISL_3355089	USA/MS-CDC-2-4524366/2021	2021/6/1	Centers for Disease Control and Prevention Division of Viral Diseases, Pathogen Discovery	Mili Sheth et al

AY.30	EPI_ISL_5030213	Thailand/DMSc-02114/2021	2021/4/6	Division of Genomic Medicine and Innovation support,Department of Medical Sciences, Ministry of Public Health, Thailand	Surakameth Mahasirimongkol et al
AY.31	EPI_ISL_3026386	SouthKorea/KDCA6298/2021	2021/6/30	Division of Emerging Infectious Diseases, Bureau of Infectious Diseases Diagnosis Control, Korea Disease Control and Prevention Agency	Jeong-Ah Kim et al
AY.33	EPI_ISL_4101637	Turkey/HSGM-E2953/2021	2021/3/9	Ministry of Health Turkey	Fatma Bayrakdar et al
AY.34	EPI_ISL_3154890	Senegal/SN-IR2-1019/2021	2021/6/1	IRESSEF	Souleymane MBOUP et al
AY.34	EPI_ISL_4460844	India/DL-NCDC-4509323/2021	2021/7/13	NCDC Delhi, Biotechnology Division - INSACOG	Kalaiarasan Ponnusamy et al
AY.37	EPI_ISL_4232132	Liberia/LIB-0233/2021	2021/3/9	Center for Infection and Immunity, Columbia University	Bode Shobayo et al
AY.38	EPI_ISL_3275133	USA/ID-UNM-IBL720559/2021	2021/3/27	Center for Global Health, University of New Mexico Health Sciences Center	Daryl Domman et al
AY.39	EPI_ISL_5703453	USA/TN-SPHL-0979/2021	2021/1/14	TN DOH Lab Services	Karen Beasley-Maynard et al
AY.39	EPI_ISL_4474077	India/JK-NCDC-3909159/2021	2021/6/1	NCDC Delhi, Biotechnology Division - INSACOG	Mahesh S Dhar et al
AY.39.1	EPI_ISL_2921622	Australia/NSW-R0509/2021	2021/7/2	Microbiology RPAH	Foster et al
AY.39.1.1	EPI_ISL_3417445	Australia/NSW-RPAH-0968/2021	2021/8/4	Microbiology RPAH	Foster et al
AY.39.2	EPI_ISL_3112511	USA/NY-CDC-LC0095673/2021	2021/7/6	Centers for Disease Control and Prevention Division of Viral Diseases, Pathogen Discovery	Dakota Howard et al
AY.4	EPI_ISL_2723338	England/NORT-1BD830A/2021	2021/6/21	COVID-19 Genomics UK (COG-UK) Consortium	Darren L Smith et al
AY.4	EPI_ISL_2925424	England/ALDP-188D2DD/2021	2021/7/1	Wellcome Sanger Institute for the COVID-19 Genomics UK (COG-UK) Consortium	Jacquelyn Wynn et al
AY.4.2	EPI_ISL_1030354	USA/CA-CZB-23359/2021	2021/1/14	Chan-Zuckerberg Biohub	CZB Cliahub Consortium et al
AY.4.2.1	EPI_ISL_2867411	England/BRBR-1827F0B/2021	2021/6/29	Wellcome Sanger Institute for the COVID-19 Genomics UK (COG-UK) Consortium	Berkshire et al
AY.4.5	EPI_ISL_2703020	England/MILK-174BF20/2021	2021/6/18	Wellcome Sanger Institute for the COVID-19 Genomics UK (COG-UK) Consortium	The Lighthouse Lab in Milton Keynes et al

AY.41	EPI_ISL_2897698	India/MH-NS2016/2021	2021/4/9	CDFD	Murali Bashyam et al
AY.43	EPI_ISL_4533969	India/GJ-INSACOG-GBRC2227/2021	2021/1/9	Gujarat Biotechnology Research Centre	Bhadreshsinh Gohil et al
AY.43	EPI_ISL_2361845	India/TN-ICMR-NIRT-INSAGOG-5593/2021	2021/5/3	NIV Influenza	Potdar et al
AY.43.1	EPI_ISL_3246106	Brazil/SP-IB_121137/2021	2021/7/19	Instituto Butantan	Dimas Tadeu Covas et al
AY.43.2	EPI_ISL_3032334	Brazil/SP-IB_119544/2021	2021/7/5	Instituto Butantan	Dimas Tadeu Covas et al
AY.45	EPI_ISL_5416409	SouthAfrica/NICD-N15251/2021	2021/1/19	National Institute for Communicable Diseases of the National Health Laboratory Service	Amoako DG et al
AY.46	EPI_ISL_4483949	Slovakia/UVZ_PL36_F7_17921/2021	2021/1/9	Laboratory of Genomics and Bioinformatics, Comenius University Science Park	Tatiana Sedláčková et al
AY.46.1	EPI_ISL_4451151	India/PB-NCDC-3509346/2021	2021/5/18	NCDC Delhi, Biotechnology Division - INSACOG	Robin Marwal et al
AY.46.2	EPI_ISL_4101849	Turkey/HSGM-E3209/2021	2021/3/9	Ministry of Health Turkey	Fatma Bayrakdar et al
AY.46.3	EPI_ISL_2880332	India/HR-IGIB-607500312049/2021	2021/4/30	INSACOG at CSIR Institute of Genomics and Integrative Biology	INSACOG et al
AY.46.4	EPI_ISL_6332970	Uganda/UG974/2021	2021/5/27	MRC/UVRI & LSHTM Uganda Research Unit , Rakai Health Sciences Program	Matthew Cotten Dan Lule Bugembe et al
AY.46.5	EPI_ISL_4307012	England/NORW-302AE27/2021	2021/5/31	COVID-19 Genomics UK (COG-UK) Consortium	Dave J. Baker et al
AY.47	EPI_ISL_1969244	Indonesia/SS-NIHRD-WGS00441/2021	2021/1/8	National Institute of Health Research and Development	Vivi Setiawaty et al
AY.49	EPI_ISL_4435563	India/DL-NCDC-300911/2021	2021/4/21	NCDC Delhi, Biotechnology Division - INSACOG	Radhakrishnan V. S et al
AY.5	EPI_ISL_1697977	England/MILK-150A709/2021	2021/4/14	Wellcome Sanger Institute for the COVID-19 Genomics UK (COG-UK) Consortium	The Lighthouse Lab in Milton Keynes et al
AY.5.1	EPI_ISL_2695446	Portugal/PT9522/2021	2021/6/4	Instituto Nacional de Saude (INSA)	Borges et al
AY.5.2	EPI_ISL_2810480	Portugal/PT10387/2021	2021/5/31	Instituto Nacional de Saude (INSA)	Borges et al
AY.5.4	EPI_ISL_3730676	Switzerland/BE-UHB-43000628/2021	2021/2/13	Clinical Bacteriology	Tim Roloff et al
AY.50	EPI_ISL_6151639	India/MH-RFIP01634/2021	2021/3/29	RF-IISER Pune	Krishanpal Karmodiya et al
AY.50	EPI_ISL_2546359	India/MH-IGIB-NIV-INSACOG-GSEQ-1977/2021	2021/3/29	NIV Influenza	Potdar et al

AY.51	EPI_ISL_2231615	India/AP-CCMB-CIA1177/2021	2021/4/20	CSIR-Centre for Cellular and Molecular Biology-INSACOG	Lamuk Zaveri et al
AY.54	EPI_ISL_1663304	India/ILSGS00608/2021	2021/3/15	Institute of Life Sciences - INSACOG	Sunil K. Raghav et al
AY.55	EPI_ISL_4768098	Rwanda/CV2200/2021	2021/1/29	Africa Centre for Excellence for Genomics of Infectious Diseases (ACEGID), Redeemer's University	Enatha Mukantwari et al
AY.56	EPI_ISL_6023787	India/DL-ILBS-104480/2021	2021/4/6	ILBS	Ekta Gupta et al
AY.57	EPI_ISL_4415497	India/MH-IISER-Pune-IP01466/2021	2021/1/9	IISER Pune-INSACOG	Krishanpal Karmodiya et al
AY.59	EPI_ISL_4101580	Malaysia/C19UMB104/2021	2021/3/17	UKM Medical Molecular Biology Institute (UMBI)	Nur Alyaa Afifah Md Shahri et al
AY.6	EPI_ISL_4884592	Italy/SIC_CQRC_2202858/2021	2021/3/16	CQRC_QUALITY CONTROL CHEMICAL BIOLOGICAL RISK_AOOR Villa Sofia Cervello Palermo	Di Gaudio F. et al
AY.61	EPI_ISL_4415492	India/MH-IISER-Pune-IP01458/2021	2021/1/9	IISER Pune-INSACOG	Krishanpal Karmodiya et al
AY.61	EPI_ISL_2180642	USA/FL-CDC-ASC210061106/2021	2021/5/8	Centers for Disease Control and Prevention Division of Viral Diseases, Pathogen Discovery	Dakota Howard et al
AY.61	EPI_ISL_3844203	India/GJ-INSACOG-GBRC1747/2021	2021/4/16	Gujarat Biotechnology Research Centre	Arpit Shukla et al
AY.62	EPI_ISL_2180457	Bangladesh/IEDCR-OIS-0016/2021	2021/5/12	IEDCR-ideSHi-icddr,b	Hassan Afrad et al
AY.62	EPI_ISL_2042958	USA/TX-CDC-ASC210034732/2021	2021/4/27	Centers for Disease Control and Prevention Division of Viral Diseases, Pathogen Discovery	Dakota Howard et al
AY.63	EPI_ISL_2673741	Norway/14196/2021	2021/6/10	Norwegian Institute of Public Health, Department of Virology	Kathrine Stene-Johansen et al
AY.65	EPI_ISL_1660427	Bahrain/840056464/2021	2021/4/10	Communicable Disease Laboratory, Public Health Directorate	Alwasti et al
AY.66	EPI_ISL_4884877	Italy/SIC_CQRC_21066357/2021	2021/3/16	CQRC_QUALITY CONTROL CHEMICAL BIOLOGICAL RISK_AOOR Villa Sofia Cervello Palermo	Di Gaudio F. et al
AY.66	EPI_ISL_4884879	Italy/SIC_CQRC_2202359/2021	2021/3/16	CQRC_QUALITY CONTROL CHEMICAL BIOLOGICAL RISK_AOOR Villa Sofia Cervello Palermo	Di Gaudio F. et al

AY.7	EPI_ISL_2006067	England/MILK-15551C5/2021	2021/5/1	Wellcome Sanger Institute for the COVID-19 Genomics UK (COG-UK) Consortium	The Lighthouse Lab in Milton Keynes et al
AY.7.1	EPI_ISL_4483966	Slovakia/UVZ_PL36_G11_17989/2021	2021/2/9	Laboratory of Genomics and Bioinformatics, Comenius University Science Park	Tatiana Sedláčková et al
AY.7.2	EPI_ISL_3039518	France/BRE_8600637102/2021	2021/6/27	CHU Pontchaillou	GROLHIER Claire et al
AY.71	EPI_ISL_2881064	India/UP-IGIB-ES_186/2021	2021/4/1	INSACOG at CSIR Institute of Genomics and Integrative Biology	INSACOG et al
AY.71	EPI_ISL_4101625	Turkey/HSGM-E2940/2021	2021/3/9	Ministry of Health Turkey	Fatma Bayrakdar et al
AY.72	EPI_ISL_3844219	India/GJ-INSACOG-GBRC1740/2021	2021/4/15	Gujarat Biotechnology Research Centre	Bhadreshsinh Gohil et al
AY.72	EPI_ISL_3144453	Italy/APU-POLBA231/2021	2021/7/16	University of Bari Biomedical Sciences and Human Oncology	Maria Chironna et al
AY.75	EPI_ISL_4062648	Oman/rega-OM-90/2021	2021/1/6	KU Leuven, Rega Institute, Clinical and Epidemiological Virology	Tony Wawina-Bokalanga et al
AY.75	EPI_ISL_2107083	India/MH-NCCS-ND16337/2021	2021/3/21	National Centre For Cell Science	Dhiraj Paul et al
AY.75	EPI_ISL_5603889	USA/IL-S21WGS5409/2021	2021/1/10	Illinois Department of Public Health - Springfield Lab	Bryan Sim et al
AY.75	EPI_ISL_2897619	India/MH-NS1904/2021	2021/4/24	CDFD	Murali Bashyam et al
AY.75	EPI_ISL_2546522	India/GA-IGIB-NIV-INSACOG-GSEQ-2659/2021	2021/4/8	NIV Influenza	Potdar et al
AY.76	EPI_ISL_2233382	Malaysia/IMR_26135/2021	2021/5/4	Institute for Medical Research, Infectious Disease Research Centre, National Institutes of Health, Ministry of Health Malaysia	Suppiah J et al
AY.78	EPI_ISL_4435813	India/DL-NCDC-3009283/2021	2021/4/22	NCDC Delhi, Biotechnology Division - INSACOG	Radhakrishnan V. S et al
AY.79	EPI_ISL_5052019	Malaysia/UNIMAS-GHML333/2021	2021/3/9	Institute of Health and Community Medicine	Chan Chia Jui et al
AY.8	EPI_ISL_1790928	England/MILK-1526901/2021	2021/4/21	Wellcome Sanger Institute for the COVID-19 Genomics UK (COG-UK) Consortium	The Lighthouse Lab in Milton Keynes et al
AY.80	EPI_ISL_3843805	India/GJ-INSACOG-GBRC1806/2021	2021/6/1	Gujarat Biotechnology Research Centre	Nitin Shukla et al
AY.82	EPI_ISL_4820060	Fiji/FJ075/2021	2021/4/25	Microbiological Diagnostic Unit - Public Health Laboratory (MDU-PHL)	Sahukhan et al

AY.84	EPI_ISL_2107500	Australia/NSW-R0239/2021	2021/4/30	Microbiology RPAH	Foster et al
AY.84	EPI_ISL_4483993	Slovakia/UVZ_PL37_B1_18000/2021	2021/4/9	Laboratory of Genomics and Bioinformatics, Comenius University Science Park	Tatiana Sedláčková et al
AY.85	EPI_ISL_4647511	Thailand/DMSc-02063/2021	2021/1/9	Division of Genomic Medicine and Innovation support,Department of Medical Sciences, Ministry of Public Health, Thailand	Surakameth Mahasirimongkol et al
AY.88	EPI_ISL_3914982	Togo/NMIMR-21-TGS-155/2021	2021/4/12	Noguchi Memorial Institute for Medical Research, University of Ghana, Legon, Ghana	Afiwa W. Halatoko et al
AY.9	EPI_ISL_3776795	England/PHEC-U307UAAE/2021	2021/3/27	COVID-19 Genomics UK (COG-UK) Consortium	PHE Covid Sequencing Team et al
AY.9.2.1	EPI_ISL_4220741	Canada/QC-L00370038001/2021	2021/7/27	Laboratoire de santé publique du Québec	Sandrine Moreira et al
AY.90	EPI_ISL_2022131	England/MILK-1557372/2021	2021/5/2	Wellcome Sanger Institute for the COVID-19 Genomics UK (COG-UK) Consortium	The Lighthouse Lab in Milton Keynes et al
AY.91.1	EPI_ISL_5327896	Namibia/CERI-KRISP-K022511/2021	2021/6/7	CERI, Centre for Epidemic Response and Innvoation, Stellenbosch University and KRISP, KZN Research Innovation and Sequencing Platform, UKZN.	Iyaloo Constantinus et al
AY.93	EPI_ISL_2479874	Canada/AB-ABPHL-17309/2021	2021/4/25	Public Health Agency of Canada (PHAC) National Microbiology Laboratory	Buss et al
AY.94	EPI_ISL_4884550	Italy/SIC_CQRC_21070336/2021	2021/3/16	CQRC_QUALITY CONTROL CHEMICAL BIOLOGICAL RISK_AOOR Villa Sofia Cervello Palermo	Di Gaudio F. et al
AY.97	EPI_ISL_1994608	SouthAfrica/KRISP-K014463_2/2021	2021/4/30	KRISP, KZN Research Innovation and Sequencing Platform	Giandhari J et al
AY.98.1	EPI_ISL_5104615	Slovenia/17-047667-CE/2020	2020/11/13	NLZOH (National Laboratory for Health, Environment and Food) / CISLD (Clinical Institute of Special Laboratory Diagnostics), University Children's Hospital, University Medical Center Ljubljana	Sandra Janezic et al
AY.99.1	EPI_ISL_2379633	India/TN-C_83_S69_R1_001/2021	2021/4/29	inStem NCBS - INSACOG	Uma Ramakrishnan Dasaradhi Palakodeti Aswin SaiNarain et al

AZ.1	EPI_ISL_4026322	Nigeria/BCVL-21182/2021	2021/1/22	Northwestern University - Center for Pathogen Genomics and Microbial Evolution	Ramon Lorenzo-Redondo et al
AZ.2	EPI_ISL_2370918	Greece/196448/2021	2021/2/5	Greek Genome Center, Biomedical Research Foundation of the Academy of Athens (BRFAA)	Emmanouil Athanasiadis et al
AZ.2.1	EPI_ISL_1361866	Germany/ST-MD1536/2021	2021/3/15	Institute of Medical Microbiology and Hospital Hygiene	Prof. Dr. Achim Kaasch et al
AZ.3	EPI_ISL_4964826	USA/TX-CDC-LC0013987/2021	2021/2/8	Centers for Disease Control and Prevention Division of Viral Diseases, Pathogen Discovery	Dakota Howard et al
AZ.4	EPI_ISL_1180300	Ireland/D-NVRL-21IRL22786/2021	2021/2/8	National Virus Reference Laboratory	Guerrino Macori et al
AZ.5	EPI_ISL_2156221	Philippines/PH-PGC-06126/2021	2021/2/9	Philippine Genome Center	Francis A. Tablizo et al
B	EPI_ISL_402123	Wuhan/IPBCAMS-WH-01/2019	2019/12/24	Institute of Pathogen Biology, Chinese Academy of Medical Sciences & Peking Union Medical College	Lili Ren et al
B	EPI_ISL_417253	England/20108006603/2020	2020/3/3	Respiratory Virus Unit, Microbiology Services Colindale, Public Health England	Monica Galiano et al
B	EPI_ISL_420796	USA/TX-CDC-3063244-001/2020	2020/2/29	Pathogen Discovery, Respiratory Viruses Branch, Division of Viral Diseases, Centers for Disease Control and Prevention	Krista Queen et al
B	EPI_ISL_457853	Kenya/P1118/2020	2020/4/19	KEMRI-Wellcome Trust Research Programme/KEMRI-CGMR-C Kilifi	Githinji G. et al 2020 et al
B	EPI_ISL_2694591	Iran/GRC-10/2020	2020/5/17	Genetics Research Center, University of Social Welfare and Rehabilitation Sciences	Zohreh Fattahi et al
B.1	EPI_ISL_4537783	France/20_B./2020	2020/1/1	APHP Bichat-Claude Bernard	Samuel Lebourgeois et al
B.1	EPI_ISL_452107	USA/LA-CDC-03069817-001/2020	2020/3/10	Pathogen Discovery, Respiratory Viruses Branch, Division of Viral Diseases, Centers for Disease Control and Prevention	Yan Li et al
B.1	EPI_ISL_3138689	Belgium/MR71NY1731/2020	2020/5/4	Microbiology Department - University Hospital Brussel	Thomas Demuyser et al
B.1	EPI_ISL_1300948	USA/NY-MSHSPSP-PV15333/2020	2020/7/27	MSHS Pathogen Surveillance Program	Ana S. Gonzalez-Reiche et al

B.1	EPI_ISL_2445035	USA/AZ-ASPHL-1991/2020	2020/7/7	Arizona State Public Health Laboratory	Trung Huynh et al
B.1	EPI_ISL_2443765	USA/CA-UCI-079/2020	2020/4/13	University of California, Irvine	Amanda N. Pinski et al
B.1	EPI_ISL_2884955	USA/MD-HP06635-PIDLEHQTMF/2020	2020/4/2	Johns Hopkins Hospital Department of Pathology	C. Paul Morris et al
B.1	EPI_ISL_2219597	USA/TX-HMH-MCoV-35067/2020	2020/8/11	Houston Methodist Hospital	Randall J. Olsen et al
B.1	EPI_ISL_769328	England/MILK-C61001/2020	2020/12/13	Wellcome Sanger Institute for the COVID-19 Genomics UK (COG-UK) Consortium	The Lighthouse Lab in Milton Keynes et al
B.1	EPI_ISL_3410636	USA/MA-UMASSMED-P006E05/2020	2020/4/30	Center for Microbiome Research	Richard T. Ellison III et al
B.1	EPI_ISL_3275378	SouthAfrica/KRISP-K016875/2021	2021/4/9	KRISP, KZN Research Innovation and Sequencing Platform	Laguda-Akingba O et al
B.1	EPI_ISL_5384449	USA/TX-HMH-MCoV-49669/2020	2020/7/11	Houston Methodist Hospital	Randall J. Olsen et al
B.1	EPI_ISL_4419636	Canada/BC-BCCDC-165732/2020	2020/10/25	BCCDC Public Health Laboratory	Prystajecky Natalie et al
B.1.1	EPI_ISL_3568416	Australia/NSW-SAVID-4085/2020	2020/1/8	Virology Research Laboratory; Area of Virology, Serology and Virology Division (SAViD), New South Wales Health Pathology Randwick	Foster et al
B.1.1	EPI_ISL_2367394	Switzerland/VD-CHUV-GEN2537/2020	2020/3/16	Laboratory of genomics and metagenomics	Trestan Pillonel et al
B.1.1	EPI_ISL_5248196	Ireland/D-U-MAY-000235/2020	2020/3/18	Irish Coronavirus Sequencing Consortium - Maynooth University	David A Fitzpatrick et al
B.1.1	EPI_ISL_465738	England/20122017204/2020	2020/3/15	Respiratory Virus Unit, Microbiology Services Colindale, Public Health England	PHE Covid Sequencing Team et al
B.1.1	EPI_ISL_470842	Australia/WA24/2020	2020/4/1	PathWest Laboratory Medicine WA	Chisha Sikazwe et al
B.1.1	EPI_ISL_512751	Australia/WA98/2020	2020/3/29	PathWest Laboratory Medicine WA Microbial Surveillance Unit	PathWest Laboratory Medicine WA Microbial Surveillance Unit et al
B.1.1	EPI_ISL_762994	NorthMacedonia/ZMC5839/2020	2020/6/3	Laboratory of Genetics and Personalized Medicine, Zan Mitrev Clinic	Kungulovski et al.
B.1.1	EPI_ISL_775881	Germany/HH-hpi-p2081/2020	2020/3/20	Heinrich Pette Institute, Leibniz Institute for Experimental Virology	Alexis Robitaille et al
B.1.1	EPI_ISL_3730873	Italy/TAA-APSS-Ph2_RUN2_NB24/2020	2020/3/31	U.O. Microbiologia e Virologia, Azienda Provinciale per i Servizi Sanitari Provincia Autonoma di Trento, Ospedale S.Chiara	Mirko Moser et al

B.1.1	EPI_ISL_2596117	France/PAC-IHU-11363_Illu1/2020	2020/3/20	MEPHI, Aix Marseille University	Anthony LEVASSEUR et al
B.1.1	EPI_ISL_1577269	Italy/CAM-CRGS-634/2021	2021/3/3	1. Genome Research Center for Health (CRGS) / 2. Laboratory of Molecular Medicine and Genomics(LMMGe) / 3. Center for Research in Pure and Applied Mathematics (CRMPA)	Giorgio Giurato (Corresponding Author) et al
B.1.1	EPI_ISL_685346	Japan/PG-1740/2020	2020/5/2	Pathogen Genomics Center, National Institute of Infectious Diseases	Tsuyoshi Sekizuka et al
B.1.1	EPI_ISL_730576	Turkey/Ankara_GUMV_10711/2020	2020/6/25	Gazi University Faculty of Medicine, Medical Virology Laboratory	Erdem Şahin et al
B.1.1	EPI_ISL_1181730	Italy/VEN-SI_125/2020	2020/3/20	Department of Molecular Medicine,Computational Medicine Group,Univeresity of Padova,Padova,Italy	Elisa Franchin et al
B.1.1	EPI_ISL_684712	Japan/PG-1102/2020	2020/4/22	Pathogen Genomics Center, National Institute of Infectious Diseases	Tsuyoshi Sekizuka et al
B.1.1	EPI_ISL_3134730	Brazil/PE-FIOCRUZ-IAM2136/2020	2020/9/2	WallauLab on behalf of Fiocruz COVID-19 Genomic Surveillance Network	Marcelo Henrique dos Santos Paiva et al
B.1.1	EPI_ISL_5074032	USA/TX-HMH-MCoV-42583/2020	2020/7/19	Houston Methodist Hospital	Randall J. Olsen et al
B.1.1	EPI_ISL_5384262	USA/TX-HMH-MCoV-49490/2020	2020/7/13	Houston Methodist Hospital	Randall J. Olsen et al
B.1.1.1	EPI_ISL_416140	Denmark/SSI-05/2020	2020/3/2	Statens Serum Institute	Morten Rasmussen et al
B.1.1.1	EPI_ISL_1534645	Peru/LIM-INS-869/2020	2020/11/30	Laboratorio de Referencia Nacional de Biotecnología y Biología Molecular. Instituto Nacional de Salud Perú	Carlos Padilla Rojas et al
B.1.1.10	EPI_ISL_602518	Germany/NW-HHU-148/2020	2020/3/1	Center of Medical Microbiology, Virology, and Hospital Hygiene, University of Duesseldorf	Olympia E. Anastasiou et al
B.1.1.100	EPI_ISL_620841	Denmark/DCGC-5516/2020	2020/9/21	Albertsen lab, Department of Chemistry and Bioscience, Aalborg University, Denmark	Danish Covid-19 Genome Consortia et al
B.1.1.101	EPI_ISL_454565	India/MH-NIV-QA-710/2020	2020/3/31	NIV Influenza	Potdar et al
B.1.1.107	EPI_ISL_473446	England/BIRM-61243/2020	2020/6/6	COVID-19 Genomics UK (COG-UK) Consortium	Institute of Microbiology et al
B.1.1.109	EPI_ISL_559070	England/ALDP-52A636/2020	2020/6/1	Wellcome Sanger Institute for the COVID-19 Genomics UK (COG-UK) consortium	The Lighthouse Lab in Alderley Park et al

B.1.1.110	EPI_ISL_1239429	Finland/24MS1A6/2020	2020/3/24	Department of Virology, Faculty of Medicine, University of Helsinki, Helsinki, Finland	Teemu Smura et al
B.1.1.111	EPI_ISL_644795	Zimbabwe/ZW-6978/2020	2020/5/1	Quadram Institute Bioscience	Thanh Le Viet et al
B.1.1.112	EPI_ISL_523614	Netherlands/ZH-EMC-272/2020	2020/5/8	Erasmus Medical Center	Bas Oude Munnink et al
B.1.1.113	EPI_ISL_467608	USA/NM-UNM-00027/2020	2020/3/15	Center for Global Health, University of New Mexico Health Sciences Center	Daryl Domman et al
B.1.1.114	EPI_ISL_432321	Wales/PHWC-27B02/2020	2020/3/30	Public Health Wales Microbiology Cardiff	Catherine Moore et al
B.1.1.115	EPI_ISL_580835	England/ALDP-9EDB5A/2020	2020/5/9	Wellcome Sanger Institute for the COVID-19 Genomics UK (COG-UK) consortium	Jacquelyn Wynn et al
B.1.1.116	EPI_ISL_497204	USA/WA-S2572/2020	2020/6/17	Seattle Flu Study	Deborah A. Nickerson et al
B.1.1.117	EPI_ISL_500460	Brazil/PE-FIOCRUZ-IAM08/2020	2020/4/7	WallauLab, Aggeu Magalhaes Institute	Marcelo Henrique Santos Paiva et al
B.1.1.118	EPI_ISL_545087	USA/TX-HMH-2050/2020	2020/5/11	Houston Methodist Hospital	S. Wesley Long et al
B.1.1.119	EPI_ISL_489205	NorthernIreland/NIRE-103831/2020	2020/4/15	Wellcome Sanger Institute for the COVID-19 Genomics UK (COG-UK) consortium	Conall McCaughey et al
B.1.1.12	EPI_ISL_559004	England/ALDP-519746/2020	2020/6/2	Wellcome Sanger Institute for the COVID-19 Genomics UK (COG-UK) consortium	The Lighthouse Lab in Alderley Park et al
B.1.1.120	EPI_ISL_559078	England/ALDP-52AAA3/2020	2020/6/1	Wellcome Sanger Institute for the COVID-19 Genomics UK (COG-UK) consortium	The Lighthouse Lab in Alderley Park et al
B.1.1.121	EPI_ISL_484153	England/GSTT-265E0F8/2020	2020/3/31	COVID-19 Genomics UK (COG-UK) Consortium	Chloe Fisher et al
B.1.1.122	EPI_ISL_444168	England/LOND-D4A07/2020	2020/4/10	COVID-19 Genomics UK (COG-UK) Consortium	Sergi Castellano et al
B.1.1.123	EPI_ISL_589485	England/ALDP-9ED64A/2020	2020/5/18	Wellcome Sanger Institute for the COVID-19 Genomics UK (COG-UK) consortium	Jacquelyn Wynn et al
B.1.1.125	EPI_ISL_461931	England/NOTT-11184F/2020	2020/5/21	COVID-19 Genomics UK (COG-UK) Consortium	Gemma Clark et al
B.1.1.127	EPI_ISL_450256	Russia/StPetersburg-RII7520S/2020	2020/4/20	WHO National Influenza Centre Russian Federation	Andrey Komissarov et al
B.1.1.128	EPI_ISL_515459	USA/NV-NSPHL-A0205/2020	2020/6/2	Nevada State Public Health Laboratory	Richard Tillett et al

B.1.1.129	EPI_ISL_430084	Russia/StPetersburg-RII5017V/2020	2020/4/9	WHO National Influenza Centre Russian Federation	Andrey Komissarov et al
B.1.1.13	EPI_ISL_524622	England/LOND-126027C/2020	2020/3/14	Wellcome Sanger Institute for the COVID-19 Genomics UK (COG-UK) consortium	Ling Li et al
B.1.1.130	EPI_ISL_536998	England/QEUH-961848/2020	2020/7/31	Wellcome Sanger Institute for the COVID-19 Genomics UK (COG-UK) consortium	Harper VanSteenhouse et al
B.1.1.132	EPI_ISL_631667	USA/TX-DSHS-1205/2020	2020/3/17	Texas Department of State Health Services	Rashmi Tuladhar et al
B.1.1.133	EPI_ISL_914979	Canada/NS-NML-912/2020	2020/3/22	National Microbiology Laboratory (NML)	Anna Majer et al
B.1.1.134	EPI_ISL_432425	Wales/PHWC-28600/2020	2020/4/1	Public Health Wales Microbiology Cardiff	Catherine Moore et al
B.1.1.135	EPI_ISL_475129	Sweden/20-50299/2020	2020/3/12	The Public Health Agency of Sweden	Oskar Karlsson Lindsjo et al
B.1.1.136	EPI_ISL_480662	Australia/VIC2020/2020	2020/6/3	VIDRL and MDU-PHL	Caly L. et al
B.1.1.137	EPI_ISL_420173	England/SHEF-C08F5/2020	2020/3/24	Department of Infection, Immunity and Cardiovascular Disease, The Florey Institute, The Medical School, University of Sheffield	Thushan de Silva et al
B.1.1.138	EPI_ISL_540217	Wales/QEUH-9A4506/2020	2020/4/4	Wellcome Sanger Institute for the COVID-19 Genomics UK (COG-UK) consortium	Harper VanSteenhouse et al
B.1.1.138	EPI_ISL_814089	Turkey/HSGM-2004082/2020	2020/5/15	Ministry of Health Turkey	Fatma Bayrakdar et al
B.1.1.139	EPI_ISL_857000	USA/CA-CZB-9973/2020	2020/6/17	Chan-Zuckerberg Biohub	CZB Cliahub Consortium et al
B.1.1.14	EPI_ISL_433117	Scotland/EDB1683/2020	2020/4/16	COVID-19 Genomics UK (COG-UK) Consortium	McHugh M et al
B.1.1.141	EPI_ISL_596264	Russia/NVS-RII-MH1087V/2020	2020/7/22	WHO National Influenza Centre Russian Federation	Andrey Komissarov et al
B.1.1.142	EPI_ISL_475831	Austria/CeMM0391/2020	2020/3/10	Bergthaler laboratory, CeMM Research Center for Molecular Medicine of the Austrian Academy of Sciences	Alexandra Popa et al
B.1.1.144	EPI_ISL_1495592	Austria/CeMM5636/2020	2020/8/25	Bergthaler laboratory, CeMM Research Center for Molecular Medicine of the Austrian Academy of Sciences	Lukas Endler et al
B.1.1.147	EPI_ISL_492193	England/CAMB-1B4820/2020	2020/6/4	Wellcome Sanger Institute for the COVID-19 Genomics UK (COG-UK) consortium	Luke W Meredith et al

B.1.1.148	EPI_ISL_470028	NorthernIreland/NIRE-FC368/2020	2020/4/12	Wellcome Sanger Institute for the COVID-19 Genomics UK (COG-UK) consortium	Conall McCaughey et al
B.1.1.149	EPI_ISL_448406	England/NOTT-110CEA/2020	2020/5/2	COVID-19 Genomics UK (COG-UK) Consortium	Gemma Clark et al
B.1.1.15	EPI_ISL_554634	England/MILK-957D82/2020	2020/6/9	Wellcome Sanger Institute for the COVID-19 Genomics UK (COG-UK) consortium	The Lighthouse Lab in Milton Keynes et al
B.1.1.152	EPI_ISL_596363	Russia/SPE-RII-MH953S/2020	2020/9/3	WHO National Influenza Centre Russian Federation	Andrey Komissarov et al
B.1.1.153	EPI_ISL_889356	CzechRepublic/IAB20_016_031/2020	2020/8/6	Institute of Applied Biotechnologies a.s.	Petr Klempt et al
B.1.1.154	EPI_ISL_432628	England/SHEF-D13D2/2020	2020/3/30	COVID-19 Genomics UK (COG-UK) Consortium	Thushan de Silva et al
B.1.1.155	EPI_ISL_559032	England/ALDP-51F142/2020	2020/6/2	Wellcome Sanger Institute for the COVID-19 Genomics UK (COG-UK) consortium	The Lighthouse Lab in Alderley Park et al
B.1.1.157	EPI_ISL_1523399	Canada/QC-HMR-97204348/2020	2020/5/20	Laboratoire de santé publique du Québec	Sandrine Moreira et al
B.1.1.158	EPI_ISL_496205	USA/WA-S1779/2020	2020/6/1	Seattle Flu Study	Deborah A. Nickerson et al
B.1.1.159	EPI_ISL_596770	Australia/WA207/2020	2020/8/14	PathWest Laboratory Medicine WA Microbial Surveillance Unit	PathWest Laboratory Medicine WA Microbial Surveillance Unit et al
B.1.1.16	EPI_ISL_434473	Greece/136/2020	2020/3/11	Laboratory of Biology, Department of Medicine, Democritus University of Thrace	Kassela K. et al
B.1.1.160	EPI_ISL_489355	NorthernIreland/NIRE-100184/2020	2020/5/3	Wellcome Sanger Institute for the COVID-19 Genomics UK (COG-UK) consortium	Conall McCaughey et al
B.1.1.161	EPI_ISL_895763	CzechRepublic/FNHK-03501/2020	2020/3/19	Molecular biology division, Institute of Clinical Biochemistry and Diagnostics, Charles University, Faculty of Medicine in Hradec Králové and University Hospital Hradec Králové	Helena Kovaříková et al
B.1.1.162	EPI_ISL_1586301	Iceland/1014/2020	2020/3/21	deCODE genetics	Daniel F Gudbjartsson et al
B.1.1.163	EPI_ISL_486829	Russia/CRIE187963/2020	2020/4/1	Group of Genomics and Postgenomic Technologies of Central Research Institute of Epidemilology	Speranskaya AS et al

B.1.1.164	EPI_ISL_479933	Japan/PG-0243/2020	2020/3/16	Pathogen Genomics Center, National Institute of Infectious Diseases	Tsuyoshi Sekizuka et al
B.1.1.165	EPI_ISL_590390	England/ALDP-9B5C0D/2020	2020/8/28	Wellcome Sanger Institute for the COVID-19 Genomics UK (COG-UK) consortium	Jacquelyn Wynn et al
B.1.1.168	EPI_ISL_458605	England/NORT-289744/2020	2020/3/31	Wellcome Sanger Institute for the COVID-19 Genomics UK (COG-UK) consortium	Chris Duncan et al
B.1.1.169	EPI_ISL_545081	USA/TX-HMH-2042/2020	2020/5/4	Houston Methodist Hospital	S. Wesley Long et al
B.1.1.17	EPI_ISL_417757	Iceland/62/2020	2020/3/6	deCODE genetics	Daniel F Gudbjartsson et al
B.1.1.170	EPI_ISL_532876	Scotland/QEUH-7B078B/2020	2020/7/19	Wellcome Sanger Institute for the COVID-19 Genomics UK (COG-UK) consortium	Ana da Silva Filipe et al
B.1.1.171	EPI_ISL_420489	England/20129040602/2020	2020/3/20	Respiratory Virus Unit, Microbiology Services Colindale, Public Health England	Monica Galiano et al
B.1.1.172	EPI_ISL_475663	USA/CA-CSMC50/2020	2020/4/6	Cedars-Sinai Medical Center, Molecular Pathology Laboratory of Department of Pathology & Laboratory Medicine and Genomic Core	Wenjuan Zhang et al
B.1.1.175	EPI_ISL_605905	Bangladesh/DNAS-RAB-isl-118/2020	2020/4/29	NGS Lab, DNA SOLUTION LTD.	Khan et al
B.1.1.176	EPI_ISL_535744	Canada/QC-LSPQ-L00216190/2020	2020/3/12	Laboratoire de santé publique du Québec	Sandrine Moreira et al
B.1.1.177	EPI_ISL_545532	USA/TX-HMH-2757/2020	2020/6/4	Houston Methodist Hospital	S. Wesley Long et al
B.1.1.178	EPI_ISL_441289	England/CAMB-7A14A/2020	2020/4/2	Wellcome Sanger Institute for the COVID-19 Genomics UK (COG-UK) consortium	Luke W Meredith et al
B.1.1.180	EPI_ISL_517875	USA/FL-BPHL-0985/2020	2020/6/8	Florida Bureau of Public Health Laboratories	Sarah Schmedes et al
B.1.1.181	EPI_ISL_586305	Canada/ON-S1230/2020	2020/3/22	McMaster University	Allison McGeer et al
B.1.1.182	EPI_ISL_582662	UnitedArabEmirates/skmc-3580730/2020	2020/9/9	Molecular/Surveillance lab Sheikh Khalifa Medical City	Amirtharaj Francis et al
B.1.1.184	EPI_ISL_569735	Russia/OMS-ORINFI-5710S/2020	2020/5/8	WHO National Influenza Centre Russian Federation	Artem Fadeev et al
B.1.1.185	EPI_ISL_455196	Netherlands/NA_394/2020	2020/3/31	Erasmus Medical Center	Bas Oude Munnink et al

B.1.1.186	EPI_ISL_1533465	USA/GA-CDC-4049061-001/2020	2020/6/25	Genomics and Discovery, Respiratory Viruses Branch, Division of Viral Diseases, Centers for Disease Control and Prevention	Yan Li et al
B.1.1.187	EPI_ISL_778771	Italy/CAM-TIGEM-452/2020	2020/6/24	TIGEM	Antonio Grimaldi et al
B.1.1.189	EPI_ISL_707793	Tunisia/9111/2020	2020/4/11	1-Clinical and Experimental Pharmacology Lab, LR16SP02, National Center of Pharmacovigilance, University of Tunis El Manar, Tunis, Tunisia. 2-Neurodegenerative diseases and psychiatric troubles, LR18SP03, Razi Hospital, University of Tunis El Manar, Tunis, Tunisia. 3- Ministry of Health, National Observatory of New and Emerging Diseases, 1006, Tunis, Tunisia	Ilhem Boutiba-Ben Boubaker et al
B.1.1.190	EPI_ISL_3568108	Australia/NSW-SAVID-3200/2020	2020/3/17	Virology Research Laboratory; Area of Virology, Serology and Virology Division (SAViD), New South Wales Health Pathology Randwick	Foster et al
B.1.1.191	EPI_ISL_654953	Sweden/20-23086/2020	2020/3/20	The Public Health Agency of Sweden	Anna-Malin Linde et al
B.1.1.192	EPI_ISL_480297	Bulgaria/06/2020	2020/3/21	NRL-HIV	Ivan Ivanov et al
B.1.1.193	EPI_ISL_600046	England/MILK-A0556C/2020	2020/9/30	Wellcome Sanger Institute for the COVID-19 Genomics UK (COG-UK) consortium	The Lighthouse Lab in Milton Keynes et al
B.1.1.194	EPI_ISL_534986	England/OXON-B49E2/2020	2020/3/13	COVID-19 Genomics UK (COG-UK) Consortium	Tanya Golubchik et al
B.1.1.196	EPI_ISL_551986	England/MILK-993397/2020	2020/8/26	Wellcome Sanger Institute for the COVID-19 Genomics UK (COG-UK) consortium	The Lighthouse Lab in Milton Keynes et al
B.1.1.197	EPI_ISL_433888	England/CAMB-7AEAD/2020	2020/4/9	COVID-19 Genomics UK (COG-UK) Consortium	Luke W Meredith et al
B.1.1.198	EPI_ISL_497864	HongKong/HKU-200723-097/2020	2020/3/18	Department of Microbiology, The University of Hong Kong	Kelvin K.W. To et al
B.1.1.200	EPI_ISL_462195	Belgium/LL-0328341/2020	2020/3/28	KU Leuven, Rega Institute, Clinical and Epidemiological Virology	Tony Wawina-Bokalanga et al

B.1.1.201	EPI_ISL_458037	India/TN-CCMB-C7/2020	2020/4/29	CSIR-Centre for Cellular and Molecular Biology	K.Kaveri et al
B.1.1.202	EPI_ISL_493330	Italy/LAZ-INMI18/2020	2020/5/10	INMI Lazzaro Spallanzani IRCCS	Cesare E.M. Gruber et al
B.1.1.203	EPI_ISL_520697	UnitedArabEmirates/L3290250769/2020	2020/5/20	Al Jalila Genomics Center	Ahmad Abou Tayoun et al
B.1.1.204	EPI_ISL_1181818	Italy/VEN-SI_139/2020	2020/3/7	Department of Molecular Medicine,Computational Medicine Group,Univeresity of Padova,Padova,Italy	Elisa Franchin et al
B.1.1.205	EPI_ISL_672264	USA/CA-CZB-10225/2020	2020/5/16	Chan-Zuckerberg Biohub	CZB Cliahub Consortium et al
B.1.1.207	EPI_ISL_2425975	Norway/2439/2020	2020/4/14	Norwegian Institute of Public Health, Department of Virology	Kathrine Stene-Johansen et al
B.1.1.208	EPI_ISL_440072	England/CAMB-72D11/2020	2020/3/22	Wellcome Sanger Institute for the COVID-19 Genomics UK (COG-UK) consortium	Luke W Meredith et al
B.1.1.209	EPI_ISL_523469	Netherlands/un-EMC-714/2020	2020/5/6	Erasmus Medical Center	Bas Oude Munnink et al
B.1.1.210	EPI_ISL_560647	USA/DE-DHSS-F918664/2020	2020/3/21	Delaware Public Health Lab	Gregory Hovan et al
B.1.1.213	EPI_ISL_424033	England/20129036204/2020	2020/3/20	Respiratory Virus Unit, Microbiology Services Colindale, Public Health England	Monica Galiano et al
B.1.1.214	EPI_ISL_1431487	Japan/PG-27639/2020	2020/2/22	Pathogen Genomics Center, National Institute of Infectious Diseases	Tsuyoshi Sekizuka et al
B.1.1.216	EPI_ISL_508504	India/HR-TF38/2020	2020/5/6	National Institute of Biomedical Genomics	Arindam Maitra et al
B.1.1.216	EPI_ISL_667577	Japan/IC-0148/2020	2020/8/3	Pathogen Genomics Center, National Institute of Infectious Diseases	Tsuyoshi Sekizuka et al
B.1.1.217	EPI_ISL_559696	England/MILK-482D1A/2020	2020/4/26	Wellcome Sanger Institute for the COVID-19 Genomics UK (COG-UK) consortium	The Lighthouse Lab in Milton Keynes et al
B.1.1.217	EPI_ISL_690984	Japan/PG-2858/2020	2020/4/13	Pathogen Genomics Center, National Institute of Infectious Diseases	Tsuyoshi Sekizuka et al
B.1.1.218	EPI_ISL_480308	Bulgaria/37/2020	2020/5/11	NRL-HIV	Ivan Ivanov et al
B.1.1.219	EPI_ISL_549128	Norway/3104/2020	2020/8/4	Norwegian Institute of Public Health, Department of Virology	Kathrine Stene-Johansen et al
B.1.1.220	EPI_ISL_501573	England/BRIS-130E0C/2020	2020/3/23	Wellcome Sanger Institute for the COVID-19 Genomics UK (COG-UK) consortium	Stephanie Hutchings et al

B.1.1.221	EPI_ISL_462211	Belgium/VHEJ-0329357/2020	2020/3/29	KU Leuven, Rega Institute, Clinical and Epidemiological Virology	Tony Wawina-Bokalanga et al
B.1.1.222	EPI_ISL_837628	Mexico/CMX-INER-0026/2020	2020/4/4	Instituto Nacional de Enfermedades Respiratorias (INER)	Celia Boukadida et al
B.1.1.222	EPI_ISL_4220071	Mexico/CMX-INMEGEN-04-07-303/2020	2020/8/21	Instituto Nacional de Medicina Genomica	Cedro-Tanda A et al
B.1.1.224	EPI_ISL_615422	Denmark/DCGC-3650/2020	2020/8/31	Albertsen lab, Department of Chemistry and Bioscience, Aalborg University, Denmark	Danish Covid-19 Genome Consortia et al
B.1.1.225	EPI_ISL_610002	USA/TX-DSHS-0866/2020	2020/6/16	Texas Department of State Health Services	Rashmi Tuladhar et al
B.1.1.226	EPI_ISL_1446540	USA/TX-TCH-TCMC04002/2020	2020/4/21	Texas Children's Microbiome Center	Ruth Ann Luna et al
B.1.1.227	EPI_ISL_554886	England/ALDP-95AA1F/2020	2020/6/13	Wellcome Sanger Institute for the COVID-19 Genomics UK (COG-UK) consortium	The Lighthouse Lab in Alderley Park et al
B.1.1.228	EPI_ISL_696265	USA/AZ-TG668340/2020	2020/5/13	TGen North	Jolene Bowers et al
B.1.1.229	EPI_ISL_788954	Italy/APU-PODV-15925058/2020	2020/8/18	Beaconlab (Bioinformatics, Evolution and Comparative Genomics lab), Dept of Biosciences, University on Milan	Iacobellis M et al
B.1.1.230	EPI_ISL_545216	USA/TX-HMH-2267/2020	2020/5/24	Houston Methodist Hospital	S. Wesley Long et al
B.1.1.231	EPI_ISL_604962	USA/UT-QDX-2572/2020	2020/3/13	Quest Diagnostics	Rosenthal et al
B.1.1.232	EPI_ISL_602509	Germany/NW-HHU-187/2020	2020/3/28	Center of Medical Microbiology, Virology, and Hospital Hygiene, University of Duesseldorf	Olympia E. Anastasiou et al
B.1.1.232	EPI_ISL_2312538	Greece/69758/2020	2020/12/6	Greek Genome Center, Biomedical Research Foundation of the Academy of Athens (BRFAA)	Emmanouil Athanasiadis et al
B.1.1.234	EPI_ISL_5917781	Romania/DJ14317/2020	2020/7/20	Medical Genetics Laboratory, Regional Centre of Medical Genetics, Emergency County Hospital Craiova	Anca-Lelia (Riza) Costache et al
B.1.1.237	EPI_ISL_472789	Wales/PHWC-15E05D/2020	2020/5/15	COVID-19 Genomics UK (COG-UK) Consortium	Catherine Moore et al
B.1.1.239	EPI_ISL_548404	USA/CA-CZB-3738/2020	2020/7/21	Chan-Zuckerberg Biohub	CZB Cliahub Consortium et al
B.1.1.240	EPI_ISL_540650	England/MILK-992152/2020	2020/8/27	COVID-19 Genomics UK (COG-UK) Consortium	Gemma Clark et al
B.1.1.241	EPI_ISL_3716388	Madagascar/IPM-05252/2020	2020/4/29	Institut Pateur de Dakar	Norosoa Razanajatovo et al

B.1.1.242	EPI_ISL_561164	Gambia/GC19-3099/2020	2020/7/25	MRCG at LSHTM Genomics lab	Abdul Karim sesay et al
B.1.1.243	EPI_ISL_512640	Ukraine/203100361/2020	2020/4/24	Respiratory Virus Unit, Microbiology Services Colindale, Public Health England	PHE Covid Sequencing Team et al
B.1.1.244	EPI_ISL_525784	USA/TX-DSHS-0225/2020	2020/4/7	Texas Department of State Health Services	Jenny Zhang et al
B.1.1.249	EPI_ISL_472490	Wales/PHWC-15C462/2020	2020/3/26	COVID-19 Genomics UK (COG-UK) Consortium	Catherine Moore et al
B.1.1.25	EPI_ISL_458372	England/BRIS-129932/2020	2020/3/31	Wellcome Sanger Institute for the COVID-19 Genomics UK (COG-UK) consortium	Stephanie Hutchings et al
B.1.1.251	EPI_ISL_812233	USA/ND-NDDH-0001/2020	2020/6/2	North Dakota Department of Health, Public Health Laboratory	Lisa Wingerter et al
B.1.1.253	EPI_ISL_580650	England/ALDP-9EDBD2/2020	2020/5/9	Wellcome Sanger Institute for the COVID-19 Genomics UK (COG-UK) consortium	Jacquelyn Wynn et al
B.1.1.254	EPI_ISL_641552	France/OCC-SC417/2020	2020/3/14	CNR Virus des Infections Respiratoires - France SUD	Antonin Bal et al
B.1.1.255	EPI_ISL_473392	England/BIRM-5FC59/2020	2020/5/21	COVID-19 Genomics UK (COG-UK) Consortium	Institute of Microbiology et al
B.1.1.256	EPI_ISL_441892	England/NOTT-110A95/2020	2020/4/30	COVID-19 Genomics UK (COG-UK) Consortium	Gemma Clark et al
B.1.1.257	EPI_ISL_414444	Netherlands/ZuidHolland_1/2020	2020/3/2	Erasmus Medical Center	David Nieuwenhuijse et al
B.1.1.258	EPI_ISL_545303	USA/TX-HMH-2422/2020	2020/5/26	Houston Methodist Hospital	S. Wesley Long et al
B.1.1.26	EPI_ISL_434241	USA/WA-S481/2020	2020/4/2	Seattle Flu Study	Chu et al
B.1.1.261	EPI_ISL_3085630	Sweden/01_SE100_21CS504527/2020	2020/3/31	Karolinska University Hospital	Jan Albert et al
B.1.1.262	EPI_ISL_473358	England/BIRM-5F9BC/2020	2020/5/14	COVID-19 Genomics UK (COG-UK) Consortium	Institute of Microbiology et al
B.1.1.263	EPI_ISL_582627	UnitedArabEmirates/skmc-35676/2020	2020/4/3	Molecular/Surveillance lab Sheikh Khalifa Medical City	Amirtharaj Francis et al
B.1.1.265	EPI_ISL_1166102	Italy/LOM-Pavia-41147/2020	2020/4/11	Molecular Virology Unit, Microbiology and Virology Depertment, Fondazione IRCCS Policlinico San Matteo, Pavia	Monica Tallarita et al
B.1.1.266	EPI_ISL_541333	CzechRepublic/NRL-6834/2020	2020/5/23	State Veterinary Institute Prague	Nagy et al
B.1.1.267	EPI_ISL_596647	Ireland/D-SVUH-R5315/2020	2020/3/24	St.Vincent's University Hospital	Mary Lucey et al

B.1.1.268	EPI_ISL_545446	USA/TX-HMH-2639/2020	2020/6/5	Houston Methodist Hospital	S. Wesley Long et al
B.1.1.269	EPI_ISL_491226	Portugal/IGC4089/2020	2020/6/1	Instituto Gulbenkian de Ciência	Cathy Paulino et al
B.1.1.27	EPI_ISL_457988	Oman/RESP-20-4153/2020	2020/3/21	Oman-NIC	Samira Al-Maruqi et al
B.1.1.270	EPI_ISL_440000	England/BRIS-121D98/2020	2020/3/21	Wellcome Sanger Institute for the COVID-19 Genomics UK (COG-UK) consortium	Stephanie Hutchings et al
B.1.1.271	EPI_ISL_583597	Austria/CeMM0512/2020	2020/3/11	Bergthaler laboratory, CeMM Research Center for Molecular Medicine of the Austrian Academy of Sciences	Alexandra Popa et al
B.1.1.272	EPI_ISL_615213	Denmark/DCGC-5282/2020	2020/8/17	Albertsen lab, Department of Chemistry and Bioscience, Aalborg University, Denmark	Danish Covid-19 Genome Consortia et al
B.1.1.273	EPI_ISL_647986	USA/IN-CDC-3778675-001/2020	2020/4/23	Pathogen Discovery, Respiratory Viruses Branch, Division of Viral Diseases, Centers for Disease Control and Prevention	Yan Li et al
B.1.1.274	EPI_ISL_442562	England/CAMB-806AC/2020	2020/4/10	Wellcome Sanger Institute for the COVID-19 Genomics UK (COG-UK) consortium	Luke W Meredith et al
B.1.1.275	EPI_ISL_483968	England/GSTT-265D4DF/2020	2020/3/19	COVID-19 Genomics UK (COG-UK) Consortium	Chloe Fisher et al
B.1.1.277	EPI_ISL_420289	England/SHEF-C0619/2020	2020/3/17	Department of Infection, Immunity and Cardiovascular Disease, The Florey Institute, The Medical School, University of Sheffield	Thushan de Silva et al
B.1.1.279	EPI_ISL_457989	Oman/RESP-20-4252/2020	2020/3/21	Oman-NIC	Samira Al-Maruqi et al
B.1.1.28	EPI_ISL_416036	Brazil/SP-14/2020	2020/3/5	Instituto Adolfo Lutz, Interdiciplinary Procedures Center, Strategic Laboratory	Claudio Tavares Sacchi et al
B.1.1.280	EPI_ISL_636843	Lithuania/MR-LUHS-Eilnr201/2020	2020/7/30	Lithuanian University of Health Sciences, Molecular cardiology lab.	Lukas Zemaitis et al
B.1.1.282	EPI_ISL_460948	Netherlands/Gelderland_48/2020	2020/3/26	Erasmus Medical Center	Bas Oude Munnink et al
B.1.1.283	EPI_ISL_479894	Japan/PG-0189/2020	2020/3/16	Pathogen Genomics Center, National Institute of Infectious Diseases	Tsuyoshi Sekizuka et al
B.1.1.284	EPI_ISL_692598	Japan/PG-4371/2020	2020/5/22	Pathogen Genomics Center, National Institute of Infectious Diseases	Tsuyoshi Sekizuka et al
B.1.1.285	EPI_ISL_2671842	Japan/20200409-129/2020	2020/1/9	Juntendo University Hospital	Koji Tuchiya et al

B.1.1.286	EPI_ISL_2883698	Canada/SK-NML-686/2020	2020/6/4	National Microbiology Laboratory (NML)	Anna Majer et al
B.1.1.289	EPI_ISL_420926	Wales/PHWC-24F09/2020	2020/3/20	Public Health Wales Microbiology Cardiff	Catherine Moore et al
B.1.1.29	EPI_ISL_446035	Wales/PHWC-2DEB8/2020	2020/4/8	Public Health Wales Microbiology Cardiff	Catherine Moore et al
B.1.1.290	EPI_ISL_495690	USA/WA-S1240/2020	2020/5/11	Seattle Flu Study	Deborah A. Nickerson et al
B.1.1.291	EPI_ISL_2233972	USA/CA-CDPH383/2020	2020/3/9	California Department of Public Health	CDPH IDLB COVIDNet et al
B.1.1.294	EPI_ISL_462150	Russia/CRIE162784/2020	2020/3/31	Group of Genomics and Postgenomic Technologies of Central Research Institute of Epidemilology	Speranskaya AS et al
B.1.1.296	EPI_ISL_512410	England/PORT-2D2041/2020	2020/3/20	COVID-19 Genomics UK (COG-UK) Consortium	Angela Beckett et al
B.1.1.297	EPI_ISL_515061	Belgium/ULG-10254/2020	2020/7/16	GIGA Medical Genomics	Keith Durkin et al
B.1.1.298	EPI_ISL_618034	Denmark/DCGC-8476/2020	2020/6/8	Albertsen lab, Department of Chemistry and Bioscience, Aalborg University, Denmark	Danish Covid-19 Genome Consortia et al
B.1.1.299	EPI_ISL_559410	England/ALDP-4A29A7/2020	2020/5/22	Wellcome Sanger Institute for the COVID-19 Genomics UK (COG-UK) consortium	The Lighthouse Lab in Alderley Park et al
B.1.1.3	EPI_ISL_440368	England/CAMB-75895/2020	2020/3/18	Wellcome Sanger Institute for the COVID-19 Genomics UK (COG-UK) consortium	Luke W Meredith et al
B.1.1.30	EPI_ISL_560392	Lithuania/C20-05-R11/2020	2020/5/18	Institute of Biotechnology, Life Sciences Center, Vilnius University and Thermo Fisher Scientific	Justinas Slikas et al
B.1.1.301	EPI_ISL_559630	England/ALDP-49F5B8/2020	2020/5/22	Wellcome Sanger Institute for the COVID-19 Genomics UK (COG-UK) consortium	The Lighthouse Lab in Alderley Park et al
B.1.1.302	EPI_ISL_534241	Sweden/20-52386/2020	2020/7/6	The Public Health Agency of Sweden	Anna-Malin Linde et al
B.1.1.304	EPI_ISL_439524	England/CAMB-77990/2020	2020/3/31	Wellcome Sanger Institute for the COVID-19 Genomics UK (COG-UK) consortium	Luke W Meredith et al
B.1.1.305	EPI_ISL_684600	Japan/PG-0990/2020	2020/4/23	Pathogen Genomics Center, National Institute of Infectious Diseases	Tsuyoshi Sekizuka et al
B.1.1.306	EPI_ISL_2758212	India/un-IRSHA-CD210761/2020	2020/3/1	Communicable Diseases, Interactive Research School for Health Affairs (IRSHA)	Shrivastava et al

B.1.1.307	EPI_ISL_532583	England/QEUH-94334B/2020	2020/7/29	Wellcome Sanger Institute for the COVID-19 Genomics UK (COG-UK) Consortium	Harper VanSteenhouse et al
B.1.1.308	EPI_ISL_549450	England/MILK-9AB648/2020	2020/8/31	COVID-19 Genomics UK (COG-UK) Consortium	Tanya Golubchik et al
B.1.1.31	EPI_ISL_569768	Russia/OMS-ORINFI-5350S/2020	2020/6/7	WHO National Influenza Centre Russian Federation	Artem Fadeev et al
B.1.1.310	EPI_ISL_556972	England/CAMC-945051/2020	2020/7/15	Wellcome Sanger Institute for the COVID-19 Genomics UK (COG-UK) consortium	Rob Howes et al
B.1.1.311	EPI_ISL_536972	England/QEUH-9613CF/2020	2020/7/29	Wellcome Sanger Institute for the COVID-19 Genomics UK (COG-UK) consortium	Harper VanSteenhouse et al
B.1.1.312	EPI_ISL_755232	Jordan/SEARCH-5424/2020	2020/8/13	Andersen lab at Scripps Research	Issa Abu-Dayyeh et al
B.1.1.315	EPI_ISL_859676	UnitedArabEmirates/2890/2020	2020/8/20	BTC, Khalifa University	Al Safar et al
B.1.1.316	EPI_ISL_1011533	USA/MA-MGH-03932/2020	2020/5/17	Infectious Disease Program, Broad Institute of Harvard and MIT	Lemieux et al
B.1.1.317	EPI_ISL_455703	Vietnam/VNHN_3085/2020	2020/3/27	Oxford University Clinical Research Unit, Hanoi, Vietnam	Nguyen Thi Tam et al
B.1.1.318	EPI_ISL_977560	Nigeria/CV702/2021	2021/1/7	African Centre of Excellence for Genomics of Infectious Diseases (ACEGID), Redeemer’s University	Olawoye I. B. et al
B.1.1.318	EPI_ISL_2166882	Canada/AB-ABPHL-08599/2021	2021/2/21	Public Health Agency of Canada (PHAC) National Microbiology Laboratory	Buss et al
B.1.1.319	EPI_ISL_417427	Belgium/VI-03027/2020	2020/3/2	KU Leuven, Clinical and Epidemiological Virology	Joan Marti-Carerras et al
B.1.1.320	EPI_ISL_542068	USA/NMDOH-2020321601/2020	2020/9/1	New Mexico Department of Health Scientific Laboratory	Ellie Johnson et al
B.1.1.322	EPI_ISL_1301488	Mexico/CMX-INER-IBT-38/2020	2020/5/7	Instituto de Biotecnología de la UNAM	Authors from IBT et al
B.1.1.323	EPI_ISL_417688	Iceland/12/2020	2020/3/1	deCODE genetics	Daniel F Gudbjartsson et al
B.1.1.324	EPI_ISL_746784	Chile/RM-72850/2020	2020/5/22	Instituto de Salud Publica de Chile	Javier Tognarelli et al
B.1.1.325	EPI_ISL_557211	England/MILK-8274D2/2020	2020/7/16	Wellcome Sanger Institute for the COVID-19 Genomics UK (COG-UK) consortium	The Lighthouse Lab in Milton Keynes et al
B.1.1.326	EPI_ISL_826805	Ecuador/EC-G-2565/2020	2020/3/23	Instituto de Salud Publica de Chile	Javier Tognarelli et al
B.1.1.327	EPI_ISL_721950	Switzerland/ZH-ETHZ-380368/2020	2020/11/22	Department of Biosystems Science and Engineering, ETH Zürich	Christian Beisel et al

B.1.1.328	EPI_ISL_512662	CostaRica/INC-0056/2020	2020/6/12	Inciensa, Instituto Costarricense de Investigación y Enseñanza en Nutrición y Salud	Francisco Duarte et al
B.1.1.329	EPI_ISL_1301696	Mexico/ROO-InDRE-IBT-11/2020	2020/6/2	Instituto de Biotecnología de la UNAM	Authors from IBT et al
B.1.1.33	EPI_ISL_457796	USA/DC-HP00052/2020	2020/3/7	Johns Hopkins Hospital Department of Pathology	Peter M. Thielen et al
B.1.1.33	EPI_ISL_2017478	Brazil/GO-HLAGYN-814562/2020	2020/6/19	HLAGYN - Laboratorio de Imunologia de Transplantes de Goias	Fernando Antonio Vinhal dos Santos et al
B.1.1.330	EPI_ISL_796017	Hebei/IVDC-03-05/2021	2021/1/2	Hebei Provincial Center for Disease Control and Prevention, Shijiazhuang, Hebei Province; National Institute for Viral Disease Control and Prevention, China CDC	Shunxiang Qi et al
B.1.1.331	EPI_ISL_419656	Austria/CeMM0003/2020	2020/2/26	Bergthaler laboratory, CeMM Research Center for Molecular Medicine of the Austrian Academy of Sciences	Alexandra Popa et al
B.1.1.332	EPI_ISL_735423	Brazil/SP-783/2020	2020/7/1	Instituto Adolfo Lutz, Interdiciplinary Procedures Center, Strategic Laboratory	Claudio Tavares Sacchi et al
B.1.1.333	EPI_ISL_2426018	Norway/7651/2020	2020/1/29	Norwegian Institute of Public Health, Department of Virology	Kathrine Stene-Johansen et al
B.1.1.334	EPI_ISL_779331	USA/WI-UW-2499/2020	2020/12/30	University of Wisconsin-Madison AIDS Vaccine Research Laboratories	Gage Moreno et al
B.1.1.335	EPI_ISL_2869911	Germany/NW-KRO-446/2020	2020/4/3	Center of Medical Microbiology, Virology, and Hospital Hygiene, University of Duesseldorf	Dennis Deschka et al
B.1.1.336	EPI_ISL_733289	Russia/KDA-RII-MH4936S/2020	2020/10/14	WHO National Influenza Centre Russian Federation	Andrey Komissarov et al
B.1.1.337	EPI_ISL_570516	USA/WA-UW-12364/2020	2020/6/27	UW Virology Lab	Pavitra Roychoudhury et al
B.1.1.338	EPI_ISL_1195353	Germany/BW-FR0586/2020	2020/12/2	Institute of Virology, Clinial Virus Genomics, Medical Center, University of Freiburg, Freiburg, Germany	Jonas Fuchs et al
B.1.1.339	EPI_ISL_1390779	Canada/QC-CHUM-2007902694A/2020	2020/3/19	Laboratoire de santé publique du Québec	Sandrine Moreira et al
B.1.1.34	EPI_ISL_3067991	SouthAfrica/Tygerberg_66/2020	2020/4/16	Division of Medical Virology, Stellenbosch University and NHLS Tygerberg Hospital	Susan Engelbrecht et al
B.1.1.340	EPI_ISL_591365	Japan/IC-0020/2020	2020/4/6	Pathogen Genomics Center, National Institute of Infectious Diseases	Tsuyoshi Sekizuka et al

B.1.1.341	EPI_ISL_589828	England/QEUH-9D0F03/2020	2020/9/14	Wellcome Sanger Institute for the COVID-19 Genomics UK (COG-UK) consortium	Harper VanSteenhouse et al
B.1.1.342	EPI_ISL_543456	USA/TX-HMH-4243/2020	2020/6/19	Houston Methodist Hospital	S. Wesley Long et al
B.1.1.343	EPI_ISL_1739654	Canada/ON-SC2318/2020	2020/11/9	McMaster University	Allison McGeer et al
B.1.1.344	EPI_ISL_1301682	Mexico/AGU-InDRE-IBT-43/2020	2020/4/9	Instituto de Biotecnología de la UNAM	Authors from IBT et al
B.1.1.345	EPI_ISL_1009196	USA/MS-UT-1751/2020	2020/11/10	Colleen B. Jonsson	Mariah K. Taylor et al
B.1.1.346	EPI_ISL_621092	Denmark/DCGC-5983/2020	2020/9/21	Albertsen lab, Department of Chemistry and Bioscience, Aalborg University, Denmark	Danish Covid-19 Genome Consortia et al
B.1.1.347	EPI_ISL_5917671	Romania/DJ13378/2020	2020/7/14	Medical Genetics Laboratory, Regional Centre of Medical Genetics, Emergency County Hospital Craiova	Anca-Lelia (Riza) Costache et al
B.1.1.348	EPI_ISL_941965	Colombia/GUR-06575/2020	2020/4/30	Centro de Investigaciones en Microbiología y Biotecnología-UR (CIMBIUR), Facultad de Ciencias Naturales, Universidad del Rosario, Bogotá, Colombia Instituto Nacional de Salud, Bogotá, Colombia Icahn School of Medicine at Mount Sinai, New York, USA	Luz Helena Patiño et al
B.1.1.349	EPI_ISL_524492	England/BRIS-133838/2020	2020/8/4	Wellcome Sanger Institute for the COVID-19 Genomics UK (COG-UK) consortium	Stephanie Hutchings et al
B.1.1.350	EPI_ISL_437094	Latvia/015/2020	2020/3/23	Latvian Biomedical Research and Study Centre	Ivars Silamiķelis et al
B.1.1.351	EPI_ISL_567220	England/QEUH-9C89E2/2020	2020/9/10	Wellcome Sanger Institute for the COVID-19 Genomics UK (COG-UK) consortium	Harper VanSteenhouse et al
B.1.1.352	EPI_ISL_2919826	USA/AZ-TG955539/2020	2020/9/29	TGen North	"Jolene Bowers et al
B.1.1.353	EPI_ISL_593741	Australia/NSW1127/2020	2020/9/8	NSW Health Pathology - Institute of Clinical Pathology and Medical Research; Westmead Hospital; University of Sydney	CIDM-PH et al.
B.1.1.354	EPI_ISL_2501532	India/AS-DIB_10072/2020	2020/5/19	Regional VRDL,Dibrugarh	B Borkakoty et al
B.1.1.355	EPI_ISL_1225757	USA/IL-IDPH-OGL-S-000928/2020	2020/6/2	Gagnon Lab, Southern Illinois University	Keith Gagnon et al

B.1.1.356	EPI_ISL_586426	Canada/ON-S67/2020	2020/3/19	McMaster University	Allison McGeer et al
B.1.1.358	EPI_ISL_2006728	USA/CA-9200066222/2020	2020/9/13	Moderna Inc.	Yamuna Paila et al
B.1.1.359	EPI_ISL_622565	Denmark/DCGC-1828/2020	2020/7/6	Albertsen lab, Department of Chemistry and Bioscience, Aalborg University, Denmark	Danish Covid-19 Genome Consortia et al
B.1.1.360	EPI_ISL_474308	Wales/PHWC-2C314/2020	2020/4/3	COVID-19 Genomics UK (COG-UK) Consortium	Catherine Moore et al
B.1.1.361	EPI_ISL_559283	England/MILK-4B620F/2020	2020/6/4	Wellcome Sanger Institute for the COVID-19 Genomics UK (COG-UK) consortium	The Lighthouse Lab in Milton Keynes et al
B.1.1.362	EPI_ISL_543882	USA/TX-HMH-2946/2020	2020/6/9	Houston Methodist Hospital	S. Wesley Long et al
B.1.1.363	EPI_ISL_432211	Wales/PHWC-2764D/2020	2020/3/30	Public Health Wales Microbiology Cardiff	Catherine Moore et al
B.1.1.364	EPI_ISL_688814	Japan/PG-5690/2020	2020/5/22	Pathogen Genomics Center, National Institute of Infectious Diseases	Tsuyoshi Sekizuka et al
B.1.1.365	EPI_ISL_558929	England/ALDP-52B8A8/2020	2020/6/1	Wellcome Sanger Institute for the COVID-19 Genomics UK (COG-UK) consortium	The Lighthouse Lab in Alderley Park et al
B.1.1.366	EPI_ISL_435124	UnitedArabEmirates/L0879/2020	2020/3/16	Al Jalila Genomics Center	Ahmad Abou Tayoun et al
B.1.1.367	EPI_ISL_544022	USA/TX-HMH-3143/2020	2020/6/11	Houston Methodist Hospital	S. Wesley Long et al
B.1.1.368	EPI_ISL_5926948	Guatemala/ASI1123/2020	2020/6/30	Asociación de Salud Integral / Clínica Familiar "Luis Ángel García"	Eduardo Arathon et al
B.1.1.369	EPI_ISL_500085	England/LIVE-A4D52/2020	2020/3/2	COVID-19 Genomics UK (COG-UK) Consortium	Sam Haldenby et al
B.1.1.37	EPI_ISL_2443745	USA/CA-UCI-040/2020	2020/4/5	University of California, Irvine	Amanda N. Pinski et al
B.1.1.370	EPI_ISL_427322	Russia/StPetersburg-RII4655S/2020	2020/4/5	WHO National Influenza Centre Russian Federation	Andrey Komissarov et al
B.1.1.371	EPI_ISL_420451	Belgium/HC-030760/2020	2020/3/7	KU Leuven, Clinical and Epidemiological Virology	Joan Marti-Carreras et al
B.1.1.372	EPI_ISL_500072	England/LIVE-A4B0D/2020	2020/3/8	COVID-19 Genomics UK (COG-UK) Consortium	Sam Haldenby et al
B.1.1.373	EPI_ISL_1400558	Russia/PRI-RII-MH820S/2020	2020/8/7	WHO National Influenza Centre Russian Federation	Andrey Komissarov et al
B.1.1.375	EPI_ISL_629391	England/MILK-AC841D/2020	2020/10/21	Wellcome Sanger Institute for the COVID-19 Genomics UK (COG-UK) consortium	The Lighthouse Lab in Milton Keynes et al
B.1.1.376	EPI_ISL_672244	USA/CA-CZB-10172/2020	2020/5/11	Chan-Zuckerberg Biohub	CZB CliaHub Consortium et al

B.1.1.379	EPI_ISL_442171	England/CAMB-7CC38/2020	2020/4/5	Wellcome Sanger Institute for the COVID-19 Genomics UK (COG-UK) consortium	Luke W Meredith et al
B.1.1.38	EPI_ISL_490740	Wales/PHWC-16590D/2020	2020/5/25	COVID-19 Genomics UK (COG-UK) Consortium	Catherine Moore et al
B.1.1.380	EPI_ISL_635381	USA/CA-ALSR-2190/2020	2020/6/12	Andersen lab at Scripps Research	SEARCH Alliance San Diego with Tracy Basler et al
B.1.1.381	EPI_ISL_536515	Peru/LIM-INS-117/2020	2020/5/4	Laboratorio de Infecciones Respiratorias Agudas	Eduardo Juscamayta Lopez et al
B.1.1.382	EPI_ISL_3068004	SouthAfrica/Tygerberg_83/2020	2020/4/19	Division of Medical Virology, Stellenbosch University and NHLS Tygerberg Hospital	Susan Engelbrecht et al
B.1.1.383	EPI_ISL_467477	SouthAfrica/KRISP-0125/2020	2020/5/13	KRISP, KZN Research Innovation and Sequencing Platform	Giandhari J et al
B.1.1.384	EPI_ISL_847890	Switzerland/GE-32815676/2020	2020/12/28	HUG, Laboratory of Virology and the Health2030 Genome Center	Samuel Cordey et al
B.1.1.385	EPI_ISL_666647	Germany/NW-HHU-360/2020	2020/9/11	Center of Medical Microbiology, Virology, and Hospital Hygiene, University of Duesseldorf	Maximilian Damagnez et al
B.1.1.386	EPI_ISL_467498	SouthAfrica/KRISP-0148/2020	2020/5/30	KRISP, KZN Research Innovation and Sequencing Platform	Giandhari J et al
B.1.1.387	EPI_ISL_733167	Russia/AST-RII-MH4130S/2020	2020/8/4	WHO National Influenza Centre Russian Federation	Andrey Komissarov et al
B.1.1.388	EPI_ISL_456154	Colombia/NAR-INS-103303/2020	2020/4/26	Instituto Nacional de Salud, Universidad Cooperativa de Colombia, Instituto Alexander von Humboldt, Imperial College-London, London School of Hygiene & Tropical Medicine	Katherine Laiton-Donato et al
B.1.1.389	EPI_ISL_512665	CostaRica/INC-0059/2020	2020/6/18	Inciensa, Instituto Costarricense de Investigación y Enseñanza en Nutrición y Salud	Francisco Duarte et al
B.1.1.39	EPI_ISL_751326	Italy/VEN-UniVR-85/2020	2020/3/23	University of Verona, Department of Biotechnology	Antonio Mori et al
B.1.1.391	EPI_ISL_520724	UnitedArabEmirates/L920633058/2020	2020/3/30	Al Jalila Genomics Center	Ahmad Abou Tayoun et al
B.1.1.392	EPI_ISL_614060	USA/VA-DCLS-1809/2020	2020/9/16	Virginia DCLS	Virginia DCLS et al
B.1.1.393	EPI_ISL_1544191	India/GJ-GBRC518/2020	2020/11/24	Gujarat Biotechnology Research Centre	Ramesh Pandit et al

B.1.1.394	EPI_ISL_693616	Portugal/PT1614/2020	2020/7/8	Instituto Nacional de Saude (INSA)	Borges et al
B.1.1.395	EPI_ISL_488032	England/NORT-2972D9/2020	2020/4/27	Wellcome Sanger Institute for the COVID-19 Genomics UK (COG-UK) consortium	Chris Duncan et al
B.1.1.396	EPI_ISL_569793	Russia/OMS-ORINFI-9295S/2020	2020/7/25	WHO National Influenza Centre Russian Federation	Artem Fadeev et al
B.1.1.397	EPI_ISL_596287	Russia/SPE-RII-MH1516S/2020	2020/9/11	WHO National Influenza Centre Russian Federation	Andrey Komissarov et al
B.1.1.398	EPI_ISL_425246	England/CAMB-7386A/2020	2020/3/31	COVID-19 Genomics UK (COG-UK) Consortium	Luke W Meredith et al
B.1.1.399	EPI_ISL_767807	Ireland/LH-NVRL-MPZ41611/2020	2020/4/15	Irish Coronavirus Sequencing Consortium - Teagasc Moorepark	Calum Walsh et al
B.1.1.4	EPI_ISL_438391	England/CAMB-71DA9/2020	2020/3/31	Wellcome Sanger Institute for the COVID-19 Genomics UK (COG-UK) consortium	Luke W Meredith et al
B.1.1.40	EPI_ISL_640080	SouthAfrica/NHLS-UCT-GS-2440/2020	2020/4/24	NHLS/UCT	Arash Iranzadeh et al
B.1.1.400	EPI_ISL_3730993	Italy/TAA-APSS-Ph2_RUN3_NB02/2020	2020/3/29	U.O. Microbiologia e Virologia, Azienda Provinciale per i Servizi Sanitari Provincia Autonoma di Trento, Ospedale S.Chiara	Mirko Moser et al
B.1.1.401	EPI_ISL_491225	Portugal/IGC4033/2020	2020/6/1	Instituto Gulbenkian de Ciência	Cathy Paulino et al
B.1.1.402	EPI_ISL_542813	USA/TX-HMH-1750/2020	2020/5/12	Houston Methodist Hospital	S. Wesley Long et al
B.1.1.403	EPI_ISL_635806	USA/CA-ALSR-3037/2020	2020/9/3	Andersen lab at Scripps Research	SEARCH Alliance San Diego with Tracy Basler et al
B.1.1.404	EPI_ISL_660477	BurkinaFaso/608/2020	2020/6/21	Centre Muraz	Abdoul-Salam Ouedraogo et al
B.1.1.405	EPI_ISL_815356	Germany/SH-Cento-37955598/2020	2020/9/14	Centogene	Peter Bauer et al
B.1.1.406	EPI_ISL_710490	Spain/AS-HUCA-232023718/2020	2020/3/25	Virology (microbiology), Hospital Universitario Central de Asturias	Castello et al
B.1.1.407	EPI_ISL_524045	Russia/SPE-RII-22478V/2020	2020/6/9	WHO National Influenza Centre Russian Federation	Andrey Komissarov et al
B.1.1.408	EPI_ISL_529663	England/HECH-259A4F5/2020	2020/7/11	COVID-19 Genomics UK (COG-UK) Consortium	Institute of Microbiology et al
B.1.1.409	EPI_ISL_516850	England/LOND-126092C/2020	2020/3/16	Wellcome Sanger Institute for the COVID-19 Genomics UK (COG-UK) consortium	Ling Li et al

B.1.1.409	EPI_ISL_1503349	USA/TX-DSHS-4988/2020	2020/7/1	Texas Department of State Health Services (TXDSHS)	Rashmi Tuladhar et al
B.1.1.41	EPI_ISL_440297	England/CAMB-73141/2020	2020/3/21	Wellcome Sanger Institute for the COVID-19 Genomics UK (COG-UK) consortium	Luke W Meredith et al
B.1.1.410	EPI_ISL_491227	Portugal/IGC4092/2020	2020/6/1	Instituto Gulbenkian de Ciência	Cathy Paulino et al
B.1.1.411	EPI_ISL_574294	Ecuador/2479/2020	2020/3/23	Instituto Nacional de Investigación en Salud Pública	Leandro Patino Patino et al
B.1.1.412	EPI_ISL_515832	SouthAfrica/KRISP-K001347/2020	2020/7/1	KRISP, KZN Research Innovation and Sequencing Platform	Giandhari J et al
B.1.1.413	EPI_ISL_566603	England/MILK-9C23DB/2020	2020/9/7	Wellcome Sanger Institute for the COVID-19 Genomics UK (COG-UK) consortium	The Lighthouse Lab in Milton Keynes et al
B.1.1.414	EPI_ISL_699946	India/MH-ACTREC-294/2020	2020/7/14	Hematopathology Laboratory, ACTREC, TMC	Hematopathology Laboratory et al
B.1.1.415	EPI_ISL_854144	Austria/CeMM1772/2020	2020/11/28	Bergthaler laboratory, CeMM Research Center for Molecular Medicine of the Austrian Academy of Sciences	Lukas Endler et al
B.1.1.416	EPI_ISL_419887	Australia/VIC191/2020	2020/3/19	Victorian Infectious Diseases Reference Laboratory and Microbiological Diagnostic Unit Public Health Laboratory, Doherty Institute	Caly L. et al
B.1.1.417	EPI_ISL_570003	Canada/ON-UHTC-0277/2020	2020/6/23	Ontario Institute for Cancer Research	Ramzi Fattouh et al
B.1.1.419	EPI_ISL_488826	England/NORT-281418/2020	2020/3/14	Wellcome Sanger Institute for the COVID-19 Genomics UK (COG-UK) consortium	Chris Duncan et al
B.1.1.420	EPI_ISL_3152081	Senegal/SC20-5366/2020	2020/8/2	LBV Le Dantec	Halimatou Diop Ndiaye et al
B.1.1.421	EPI_ISL_693559	Portugal/PT1546/2020	2020/6/20	Instituto Nacional de Saude (INSA)	Borges et al
B.1.1.422	EPI_ISL_860817	Belarus/ChVir21889/2020	2020/10/26	Charité Universitätsmedizin Berlin, Institut für Virologie	Victor M Corman et al
B.1.1.423	EPI_ISL_513872	USA/CA-CZB-2467/2020	2020/5/19	Chan-Zuckerberg Biohub	CZB Cliahub Consortium et al
B.1.1.424	EPI_ISL_678283	Russia/SAR-RARI-3573/2020	2020/5/2	Mikrobiologie, RARI	Krasnov et al
B.1.1.425	EPI_ISL_552330	England/MILK-994E59/2020	2020/8/25	Wellcome Sanger Institute for the COVID-19 Genomics UK (COG-UK) consortium	The Lighthouse Lab in Milton Keynes et al
B.1.1.426	EPI_ISL_2773937	USA/LA-UW-5500544/2020	2020/6/16	UW Virology Lab	Pavitra Roychoudhury et al

B.1.1.427	EPI_ISL_732995	Russia/SVE-RII-MH216S/2020	2020/7/25	WHO National Influenza Centre Russian Federation	Andrey Komissarov et al
B.1.1.428	EPI_ISL_626060	Denmark/DCGC-8747/2020	2020/10/26	Albertsen lab, Department of Chemistry and Bioscience, Aalborg University, Denmark	Danish Covid-19 Genome Consortia et al
B.1.1.429	EPI_ISL_770056	Latvia/222/2020	2020/9/15	Latvian Biomedical Research and Study Centre	Ivars Silamiķelis et al
B.1.1.43	EPI_ISL_433327	Scotland/CVR702/2020	2020/3/25	COVID-19 Genomics UK (COG-UK) Consortium	Ana da Silva Filipe et al
B.1.1.431	EPI_ISL_521499	Australia/VIC2653/2020	2020/7/1	VIDRL and MDU-PHL	Caly L. et al
B.1.1.432	EPI_ISL_872097	Mexico/VER-InDRE-104/2020	2020/4/24	Instituto de Diagnostico y Referencia Epidemiologicos (INDRE)	Abril Rodriguez-Maldonado et al
B.1.1.433	EPI_ISL_1498929	Slovenia/66807/2020	2020/8/9	Institute of Microbiology and Immunology, Faculty of Medicine, University of Ljubljana	Alen Suljič et al
B.1.1.434	EPI_ISL_3098576	USA/MD-HP07302-PIDOBBJPQM/2020	2020/6/1	Johns Hopkins Hospital Department of Pathology	C. Paul Morris et al
B.1.1.435	EPI_ISL_732663	Russia/MOS-CRIE-13385391/2020	2020/11/6	Group of Genomics and Postgenomic Technologies of Central Research Institute of Epidemilology	Samoilov AE et al
B.1.1.436	EPI_ISL_420416	Belgium/FMC-0323159/2020	2020/3/23	KU Leuven, Clinical and Epidemiological Virology	Joan Marti-Carreras et al
B.1.1.437	EPI_ISL_4418817	USA/MD-HP09200-PIDKCKUCMM/2020	2020/5/14	Johns Hopkins Hospital Department of Pathology	C. Paul Morris et al
B.1.1.438	EPI_ISL_660384	Sweden/20-22286/2020	2020/3/12	The Public Health Agency of Sweden	Anna-Malin Linde et al
B.1.1.44	EPI_ISL_491093	CzechRepublic/NRL_4039/2020	2020/3/18	The National Institute of Public Health and State Veterinary Institute Prague	Nagy et al
B.1.1.440	EPI_ISL_522352	USA/TX-DSHS-0066/2020	2020/5/9	Texas Department of State Health Services	Rashmi Tuladhar et al
B.1.1.441	EPI_ISL_631464	USA/WI-WSLH-200707/2020	2020/6/26	Wisconsin State Laboratory of Hygiene Communicable Disease Division	Kelsey R. Florek et al
B.1.1.442	EPI_ISL_981038	Argentina/PAIS-F0040/2020	2020/5/16	Laboratorio Central Mg. Luis Alfredo Pianciola on behalf of 'Proyecto Argentino Interinstitucional de genomica de SARS-CoV-2' (PAIS Consortium)	Liliana Fonseca et al
B.1.1.444	EPI_ISL_515762	SouthAfrica/KRISP-K002398/2020	2020/7/27	KRISP, KZN Research Innovation and Sequencing Platform	Giandhari J et al

B.1.1.445	EPI_ISL_733497	Switzerland/ZH-UZH-IMV127/2020	2020/12/14	Institute of Medical Virology, University of Zurich	Stefan Schmutz et al
B.1.1.446	EPI_ISL_2643656	Estonia/Cov0223/2020	2020/9/14	Department of Microbiology, Institute of Biomedicine and Translational Medicine, University of Tartu	Radko Avi et al
B.1.1.447	EPI_ISL_516698	USA/VA-DCLS-0824/2020	2020/5/5	Virginia DCLS	Virginia DCLS et al
B.1.1.448	EPI_ISL_2444003	SouthAfrica/NICD-N00163/2020	2020/5/4	National Institute for Communicable Diseases of the National Health Laboratory Service	Amoako DG et al
B.1.1.449	EPI_ISL_596250	Russia/SPE-RII-MH1007S/2020	2020/9/4	WHO National Influenza Centre Russian Federation	Andrey Komissarov et al
B.1.1.45	EPI_ISL_559611	England/ALDP-49F3BE/2020	2020/5/22	Wellcome Sanger Institute for the COVID-19 Genomics UK (COG-UK) consortium	The Lighthouse Lab in Alderley Park et al
B.1.1.450	EPI_ISL_738746	USA/CA-CZB-15072/2020	2020/11/20	Chan-Zuckerberg Biohub	CZB Cliahub Consortium et al
B.1.1.451	EPI_ISL_635143	Norway/4276/2020	2020/10/10	Norwegian Institute of Public Health, Department of Virology	Kathrine Stene-Johansen et al
B.1.1.452	EPI_ISL_639949	Russia/PER-RII-MH1647S/2020	2020/9/10	WHO National Influenza Centre Russian Federation	Andrey Komissarov et al
B.1.1.453	EPI_ISL_754963	USA/CA-CDPH090/2020	2020/8/5	California Department of Public Health	CDPH IDLB COVIDNet et al
B.1.1.456	EPI_ISL_487319	SouthAfrica/KRISP-0368/2020	2020/6/18	KRISP, KZN Research Innovation and Sequencing Platform	Giandhari J et al
B.1.1.458	EPI_ISL_469059	Sweden/20-14647/2020	2020/5/14	The Public Health Agency of Sweden	Oskar Karlsson Lindsjo et al
B.1.1.459	EPI_ISL_640096	SouthAfrica/NHLS-UCT-GP-5016/2020	2020/4/15	NHLS/UCT	Arash Iranzadeh et al
B.1.1.46	EPI_ISL_447587	India/TN-CCMB-C4-15/2020	2020/4/20	CSIR-Centre for Cellular and Molecular Biology	K Kaveri et al
B.1.1.462	EPI_ISL_511770	Portugal/PT1227/2020	2020/3/25	Instituto Nacional de Saude (INSA) and Instituto Gulbenkian de Ciencia (IGC)	Borges et al
B.1.1.463	EPI_ISL_672258	USA/CA-CZB-10202/2020	2020/5/11	Chan-Zuckerberg Biohub	CZB Cliahub Consortium et al
B.1.1.464	EPI_ISL_2896976	USA/WI-MCW-CA085/2020	2020/4/1	Infectious Disease Core Research, Abbott Diagnostics Division	Forberg et al
B.1.1.465	EPI_ISL_4418186	Japan/CA001/2020	2020/4/21	Department of Emerging Infectious Diseases, Institute of Tropical Medicine, Nagasaki University	Haruka Abe et al
B.1.1.466	EPI_ISL_561105	Gambia/GC19-2427/2020	2020/7/21	MRCG at LSHTM Genomics lab	Abdul Karim sesay et al

B.1.1.467	EPI_ISL_633056	USA/NY-NYCPHL-001044/2020	2020/10/8	New York City Public Health Laboratory	Jade Wang et al
B.1.1.47	EPI_ISL_489977	Switzerland/VD-ETHZ-180039/2020	2020/7/1	Department of Biosystems Science and Engineering, ETH Zürich	Christian Beisel et al
B.1.1.48	EPI_ISL_685524	Japan/PG-1918/2020	2020/3/27	Pathogen Genomics Center, National Institute of Infectious Diseases	Tsuyoshi Sekizuka et al
B.1.1.480	EPI_ISL_695809	USA/AZ-TG640888/2020	2020/4/13	TGen North	Jolene Bowers et al
B.1.1.481	EPI_ISL_733180	Russia/AST-RII-MH4157S/2020	2020/8/18	WHO National Influenza Centre Russian Federation	Andrey Komissarov et al
B.1.1.482	EPI_ISL_459686	Scotland/GCVR-17027D/2020	2020/3/24	Wellcome Sanger Institute for the COVID-19 Genomics UK (COG-UK) consortium	Ana da Silva Filipe et al
B.1.1.483	EPI_ISL_745217	Belgium/UGent-332/2020	2020/12/11	Onderzoeksgroep Virologie	Nick Vereecke et al
B.1.1.484	EPI_ISL_729926	Nigeria/NA-CV240/2020	2020/5/31	African Centre of Excellence for Genomics of Infectious Diseases (ACEGID), Redeemer's University, Ede, Osun State, Nigeria	Oluniyi P.E. et al
B.1.1.485	EPI_ISL_591210	Canada/ON-S737/2020	2020/4/9	McMaster University	Allison McGeer et al
B.1.1.486	EPI_ISL_802165	USA/NY-MSHSPSP-PV16551/2020	2020/8/13	MSHS Pathogen Surveillance Program	Ana S. Gonzalez-Reiche et al
B.1.1.487	EPI_ISL_654794	Nigeria/NG57839/2020	2020/3/22	Centre for Human Virology & Genomics, Nigerian Institute of Medical Research	Shaibu et al
B.1.1.49	EPI_ISL_432384	Wales/PHWC-27249/2020	2020/3/30	Public Health Wales Microbiology Cardiff	Catherine Moore et al
B.1.1.5	EPI_ISL_417551	Iceland/187/2020	2020/3/16	deCODE genetics	Daniel F Gudbjartsson et al
B.1.1.50	EPI_ISL_447450	Israel/13075703/2020	2020/3/29	Stern Lab	Stern Lab et al
B.1.1.500	EPI_ISL_470903	Russia/Moscow_PMVL-10/2020	2020/4/10	Pathogenic Microorganisms Variability Laboratory	Alexey Shchetinin et al
B.1.1.506	EPI_ISL_569742	Russia/OMS-ORINFI-7121S/2020	2020/5/14	WHO National Influenza Centre Russian Federation	Artem Fadeev et al
B.1.1.51	EPI_ISL_538900	England/LEED-2AA3C1/2020	2020/3/30	Wellcome Sanger Institute for the COVID-19 Genomics UK (COG-UK) consortium	Louissa Macfarlane-Smith et al
B.1.1.512	EPI_ISL_635635	USA/CA-ALSR-2692/2020	2020/7/21	Andersen lab at Scripps Research	SEARCH Alliance San Diego with Tracy Basler et al
B.1.1.513	EPI_ISL_525622	USA/NY-Wadsworth-22513-01/2020	2020/3/16	Wadsworth Center, New York State Department of Health	Kirsten St. George et al
B.1.1.514	EPI_ISL_583187	USA/CA-CZB-6113/2020	2020/5/1	Chan-Zuckerberg Biohub	CZB Cliahub Consortium et al

B.1.1.515	EPI_ISL_428490	Taiwan/78/2020	2020/3/16	Centers for Disease Control, R.O.C. (Taiwan)	Ji-Rong Yang et al
B.1.1.516	EPI_ISL_1517405	CostaRica/HNN-0361/2020	2020/6/2	Inciensa, Instituto Costarricense de Investigación y Enseñanza en Nutrición y Salud	Cristian Peréz-Corrales et al
B.1.1.517	EPI_ISL_3536877	USA/CA-CDPH1435/2020	2020/12/7	California Department of Public Health	CDPH IDLB COVIDNet et al
B.1.1.518	EPI_ISL_4219952	Mexico/CMX-INMEGEN-04-07-31/2020	2020/6/5	Instituto Nacional de Medicina Genomica	Cedro-Tanda A et al
B.1.1.519	EPI_ISL_2222621	USA/TX-HMH-MCoV-39891/2020	2020/8/6	Houston Methodist Hospital	Randall J. Olsen et al
B.1.1.519	EPI_ISL_3151148	Mexico/YUC-NYGC-39037-20/2020	2020/8/28	New York Genome Center	Michael Zody et al
B.1.1.519	EPI_ISL_1841710	Finland/2342/2021	2021/2/14	Department of Virology, Faculty of Medicine, University of Helsinki, Helsinki, Finland	Teemu Smura et al
B.1.1.52	EPI_ISL_2444005	SouthAfrica/NICD-N00165/2020	2020/4/11	National Institute for Communicable Diseases of the National Health Laboratory Service	Amoako DG et al
B.1.1.528	EPI_ISL_745141	SouthAfrica/Tygerberg-460/2020	2020/12/7	National Health Laboratory Service (NHLS), Tygerberg	Susan Engelbrecht et al
B.1.1.529	EPI_ISL_6825395	SouthAfrica/CERI-KRISP-K032355/2021	2021/11/16	CERI, Centre for Epidemic Response and Innvoation, Stellenbosch University and KRISP, KZN Research Innovation and Sequencing Platform, UKZN.	Amy Strydom et al
B.1.1.53	EPI_ISL_490277	SouthAfrica/R10427/2020	2020/6/4	National Institute for Communicable Diseases of the National Health Laboratory Service	Allam M et al
B.1.1.54	EPI_ISL_507973	USA/MN-MDH-1386/2020	2020/3/19	Minnesota Department of Health, Public Health Laboratory	Matt Plumb et al
B.1.1.55	EPI_ISL_441501	England/NOTT-110330/2020	2020/4/12	COVID-19 Genomics UK (COG-UK) Consortium	Gemma Clark et al
B.1.1.56	EPI_ISL_467493	SouthAfrica/KRISP-0143/2020	2020/5/21	KRISP, KZN Research Innovation and Sequencing Platform	Giandhari J et al
B.1.1.57	EPI_ISL_455639	SouthAfrica/KRISP-109/2020	2020/3/31	KRISP, KZN Research Innovation and Sequencing Platform	Giandhari J et al
B.1.1.58	EPI_ISL_1854982	Italy/LAZ-AMC-2004088031-DS/2020	2020/4/8	Virology Laboratory, Scientific Department, Army Medical Center	Silvia Fillo et al
B.1.1.59	EPI_ISL_479385	Wales/PHWC-164E0F/2020	2020/6/1	COVID-19 Genomics UK (COG-UK) Consortium	Catherine Moore et al

B.1.1.61	EPI_ISL_419557	USA/GA-CDC-03063103-001/2020	2020/2/29	Pathogen Discovery, Respiratory Viruses Branch, Division of Viral Diseases, Centers for Disease Control and Prevention	Ying Tao et al
B.1.1.62	EPI_ISL_3068127	SouthAfrica/Tygerberg_257/2020	2020/5/7	Division of Medical Virology, Stellenbosch University and NHLS Tygerberg Hospital	Susan Engelbrecht et al
B.1.1.63	EPI_ISL_3105881	Philippines/PH-RITM-0074/2020	2020/6/23	Research Institute for Tropical Medicine	Lei Lanna Dancel et al
B.1.1.67	EPI_ISL_776018	Germany/HH-hpi-p1516/2020	2020/3/15	Heinrich Pette Institute, Leibniz Institute for Experimental Virology	Alexis Robitaille et al
B.1.1.7	EPI_ISL_1603195	Spain/CL-COV00781/2020	2020/2/7	SARS-CoV-2 Sequencing Castilla y Leon-Spain Consortium	Antonio Orduña-Domingo et al
B.1.1.7	EPI_ISL_1100034	England/RAND-12E0265/2021	2021/2/10	Wellcome Sanger Institute for the COVID-19 Genomics UK (COG-UK) Consortium	Randox Laboratories et al
B.1.1.70	EPI_ISL_856906	Italy/UMB-ISS-3867/2020	2020/3/6	Virology Laboratory, Scientific Department, Army Medical Center	Paola Stefanelli et al
B.1.1.71	EPI_ISL_419748	Australia/VIC29/2020	2020/3/10	Victorian Infectious Diseases Reference Laboratory and Microbiological Diagnostic Unit Public Health Laboratory, Doherty Institute	Caly L. et al
B.1.1.72	EPI_ISL_577556	USA/MI-MDHHS-SC22003/2020	2020/8/20	Michigan Department of Health and Human Services, Bureau of Laboratories	Blankenship HM et al
B.1.1.74	EPI_ISL_448972	NorthernIreland/NIRE-FB730/2020	2020/3/25	COVID-19 Genomics UK (COG-UK) Consortium	Conall McCaughey et al
B.1.1.75	EPI_ISL_468744	Belgium/UGent-96/2020	2020/3/17	Onderzoeksgroep Virologie	Nick Vereecke et al
B.1.1.77	EPI_ISL_541158	USA/FL-BPHL-1234/2020	2020/5/28	Florida Bureau of Public Health Laboratories, Florida Department of Health	Schmedes et al
B.1.1.8	EPI_ISL_604245	USA/NY-QDX-1894/2020	2020/3/15	Quest Diagnostics	Rosenthal et al
B.1.1.82	EPI_ISL_445688	Wales/PHWC-2ACA2/2020	2020/4/6	Public Health Wales Microbiology Cardiff	Catherine Moore et al
B.1.1.83	EPI_ISL_441399	NorthernIreland/NIRE-1022DA/2020	2020/3/23	COVID-19 Genomics UK (COG-UK) Consortium	Conall McCaughey et al
B.1.1.84	EPI_ISL_487316	SouthAfrica/KRISP-0365/2020	2020/6/25	KRISP, KZN Research Innovation and Sequencing Platform	Giandhari J et al

B.1.1.86	EPI_ISL_559631	England/ALDP-49F6A6/2020	2020/5/22	Wellcome Sanger Institute for the COVID-19 Genomics UK (COG-UK) consortium	The Lighthouse Lab in Alderley Park et al
B.1.1.87	EPI_ISL_526215	Hungary/MH-597/2020	2020/4/10	National Laboratory of Virology, Szentágothai Research Centre	Endre Gábor Tóth et al
B.1.1.88	EPI_ISL_454237	Portugal/PT0513/2020	2020/3/20	Instituto Nacional de Saude (INSA)	Borges et al
B.1.1.89	EPI_ISL_465000	England/20118179304/2020	2020/3/12	Respiratory Virus Unit, Microbiology Services Colindale, Public Health England	PHE Covid Sequencing Team et al
B.1.1.90	EPI_ISL_3085643	Sweden/01_SE100_21CS504562/2020	2020/4/24	Karolinska University Hospital	Jan Albert et al
B.1.1.91	EPI_ISL_3946506	Sweden/01_SE100_21CS508530/2020	2020/4/10	Karolinska University Hospital	Jan Albert et al
B.1.1.92	EPI_ISL_555640	England/ALDP-94477B/2020	2020/6/7	Wellcome Sanger Institute for the COVID-19 Genomics UK (COG-UK) consortium	The Lighthouse Lab in Alderley Park et al
B.1.1.93	EPI_ISL_636089	USA/CA-ALSR-3570/2020	2020/6/16	Andersen lab at Scripps Research	SEARCH Alliance San Diego with Tracy Basler et al
B.1.1.95	EPI_ISL_490775	Wales/PHWC-165BAD/2020	2020/4/3	COVID-19 Genomics UK (COG-UK) Consortium	Catherine Moore et al
B.1.1.97	EPI_ISL_589454	England/ALDP-9EE272/2020	2020/5/6	Wellcome Sanger Institute for the COVID-19 Genomics UK (COG-UK) consortium	Jacquelyn Wynn et al
B.1.1.98	EPI_ISL_696373	USA/AZ-TG670175/2020	2020/4/5	TGen North	Jolene Bowers et al
B.1.1.99	EPI_ISL_493744	Scotland/CVR1838/2020	2020/4/7	COVID-19 Genomics UK (COG-UK) Consortium	Ana da Silva Filipe et al
B.1.103	EPI_ISL_565968	USA/MI-MDHHS-SC21907/2020	2020/4/9	Michigan Department of Health and Human Services, Bureau of Laboratories	Blankenship HM et al
B.1.104	EPI_ISL_431080	USA/CT-Yale-079/2020	2020/3/2	Grubaugh Lab - Yale School of Public Health	Joseph Fauver et al
B.1.105	EPI_ISL_440240	England/CAMB-73114/2020	2020/3/21	Wellcome Sanger Institute for the COVID-19 Genomics UK (COG-UK) consortium	Luke W Meredith et al
B.1.106	EPI_ISL_575081	USA/UT-UPHL-02807/2020	2020/4/23	Utah Public Health Laboratory	Erin Young et al
B.1.108	EPI_ISL_418895	USA/CT-UW328/2020	2020/3/14	UW Virology Lab	Pavitra Roychoudhury et al

B.1.110	EPI_ISL_424855	USA/FL-CDC-03067097-001/2020	2020/3/2	Pathogen Discovery, Respiratory Viruses Branch, Division of Viral Diseases, Centers for Disease Control and Prevention	Yan Li et al
B.1.110.1	EPI_ISL_801603	Chile/Santiago-PUC_MVL_0011/2020	2020/3/18	MSHS Pathogen Surveillance Program	Leonardo I. Almonacid et al
B.1.110.2	EPI_ISL_539017	England/LEED-2A87C7/2020	2020/4/3	Wellcome Sanger Institute for the COVID-19 Genomics UK (COG-UK) consortium	Louissa Macfarlane-Smith et al
B.1.110.3	EPI_ISL_460047	USA/MN-MDH-106/2020	2020/3/15	Minnesota Department of Health, Public Health Laboratory	Matt Plumb et al
B.1.111	EPI_ISL_424850	USA/IL-CDC-03068473-001/2020	2020/3/7	Pathogen Discovery, Respiratory Viruses Branch, Division of Viral Diseases, Centers for Disease Control and Prevention	Yan Li et al
B.1.112	EPI_ISL_623194	USA/UT-UPHL-201114999/2020	2020/4/6	Utah Public Health Laboratory	Erin Young et al
B.1.113	EPI_ISL_469038	India/GJ-GBRC193b/2020	2020/6/3	Gujarat Biotechnology Research Centre	Komal Patel et al
B.1.115	EPI_ISL_447309	Israel/701002314/2020	2020/3/21	Stern Lab	Stern Lab et al
B.1.116	EPI_ISL_683682	USA/MN-MDH-2053/2020	2020/3/29	Minnesota Department of Health, Public Health Laboratory	Alexandra Lorentz et al
B.1.117	EPI_ISL_466120	England/20149006704/2020	2020/3/30	Respiratory Virus Unit, Microbiology Services Colindale, Public Health England	PHE Covid Sequencing Team et al
B.1.118	EPI_ISL_614581	Denmark/DCGC-991/2020	2020/3/9	Albertsen lab, Department of Chemistry and Bioscience, Aalborg University, Denmark	Danish Covid-19 Genome Consortia et al
B.1.119	EPI_ISL_436808	USA/MI-MDHHS-SC20179/2020	2020/3/12	Michigan Department of Health and Human Services, Bureau of Laboratories	Blankenship HM et al
B.1.12	EPI_ISL_420442	Belgium/BGM-030444/2020	2020/3/4	KU Leuven, Clinical and Epidemiological Virology	Joan Marti-Carreras et al
B.1.120	EPI_ISL_422216	Wales/PHWC-26ACA/2020	2020/3/27	Public Health Wales Microbiology Cardiff	Catherine Moore et al
B.1.124	EPI_ISL_434164	USA/WA-S404/2020	2020/3/26	Seattle Flu Study	Chu et al
B.1.126	EPI_ISL_475232	USA/CA-11567/2020	2020/5/5	UNMC COVID-19 Response Team	UNMC COVID-19 Response Team et al
B.1.127	EPI_ISL_625897	Denmark/DCGC-8549/2020	2020/10/26	Albertsen lab, Department of Chemistry and Bioscience, Aalborg University, Denmark	Danish Covid-19 Genome Consortia et al

B.1.128	EPI_ISL_2367357	Switzerland/VD-CHUV-GEN2477/2020	2020/3/9	Laboratory of genomics and metagenomics	Trestan Pillonel et al
B.1.13	EPI_ISL_418668	England/20110136302/2020	2020/3/9	Respiratory Virus Unit, Microbiology Services Colindale, Public Health England	Monica Galiano et al
B.1.131	EPI_ISL_979970	Ukraine/Vinnytsia_12/2020	2020/5/27	Department of Respiratory and other Viral Infections of L.V.Gromashevsky Institute of Epidemiology & Infectious Diseases NAMS of Ukraine, JSC "Farmak"	Alla Mironenko et al
B.1.134	EPI_ISL_3730960	Italy/TAA-APSS-Ph2_RUN1_NB15/2020	2020/4/3	U.O. Microbiologia e Virologia, Azienda Provinciale per i Servizi Sanitari Provincia Autonoma di Trento, Ospedale S.Chiara	Mirko Moser et al
B.1.137	EPI_ISL_470670	USA/UT-02054/2020	2020/4/20	Utah Public Health Laboratory	Erin Young et al
B.1.139	EPI_ISL_4025983	USA/IL-NM-0293/2020	2020/3/26	Northwestern University - Center for Pathogen Genomics and Microbial Evolution	Ramon Lorenzo-Redondo et al
B.1.14	EPI_ISL_435660	USA/CA-SCCPHD-UC181/2020	2020/3/16	Chiu Laboratory, University of California, San Francisco	Xianding Deng et al
B.1.140	EPI_ISL_5422012	USA/NE-MP_NCOV20-9850/2020	2020/4/30	NPHL COVID-19 Response Team	NPHL COVID-19 Response Team et al
B.1.142	EPI_ISL_429160	Sweden/20-50312/2020	2020/3/12	The Public Health Agency of Sweden	Martin Sundqvist et al
B.1.143	EPI_ISL_429770	Luxembourg/LNS4530810/2020	2020/3/28	Laboratoire National de Sante, Microbiology, Epidemiology and Microbial Genomics	Anke Wienecke-Baldacchino et al
B.1.145	EPI_ISL_435056	India/GJ-GBRC9/2020	2020/4/21	Gujarat Biotechnology Research Centre	Maharshi Pandya et al
B.1.146	EPI_ISL_486535	Switzerland/BE-ETHZ-170023/2020	2020/6/23	Department of Biosystems Science and Engineering, ETH Zürich	Christian Beisel et al
B.1.149	EPI_ISL_2307340	Japan/YCH0069/2020	2020/3/31	Genome Analysis Center, Yamanashi Central Hospital	Yosuke Hirotsu et al
B.1.151	EPI_ISL_753918	Germany/BE-ChVir-D762-6625/2020	2020/3/13	Charité Universitätsmedizin Berlin, Institut für Virologie	Victor M Corman et al
B.1.153	EPI_ISL_450108	USA/NY-PV09431/2020	2020/3/11	MSHS Pathogen Surveillance Program	Ana S. Gonzalez-Reiche et al
B.1.157	EPI_ISL_428679	Spain/MD-H12-LP23-5852/2020	2020/3/8	Hospital Universitario 12 de Octubre	Elias Dahdouh et al

B.1.158	EPI_ISL_418390	Finland/13M33/2020	2020/3/13	Department of Virology, Faculty of Medicine, University of Helsinki, Helsinki, Finland	Teemu Smura et al
B.1.159	EPI_ISL_940432	France/IDF_HB_112003063212/2020	2020/3/17	IAME UMR1137 Inserm, Université de Paris, Hôpital Bichat	Antoine Bridier-Nahmias et al
B.1.160	EPI_ISL_4899892	Morocco/INH-106/2020	2020/2/2	Functional Genomic Platform UATRS-biology, CNRST	Hicham Oumzil et al
B.1.160.10	EPI_ISL_3357877	France/IDF-APHPSorbonneUniversite_632/2020	2020/8/27	Department of Virology, Pitié-Salpêtrière hospital	Valentin Leducq et al
B.1.160.11	EPI_ISL_603549	Switzerland/BL-ETHZ-310479/2020	2020/10/8	Department of Biosystems Science and Engineering, ETH Zürich	Christian Beisel et al
B.1.160.13	EPI_ISL_578025	Netherlands/ZH-EMC-597/2020	2020/9/18	Erasmus Medical Center	Bas Oude Munnink et al
B.1.160.14	EPI_ISL_541417	Switzerland/BE-ETHZ-270048/2020	2020/9/1	Department of Biosystems Science and Engineering, ETH Zürich	Christian Beisel et al
B.1.160.15	EPI_ISL_603297	Switzerland/UR-ETHZ-300265/2020	2020/9/30	Department of Biosystems Science and Engineering, ETH Zürich	Christian Beisel et al
B.1.160.16	EPI_ISL_603405	Switzerland/BE-ETHZ-300453/2020	2020/9/30	Department of Biosystems Science and Engineering, ETH Zürich	Christian Beisel et al
B.1.160.16	EPI_ISL_2757556	France/PAC-IHU-13469_Nova1/2020	2020/9/15	MEPHI, Aix Marseille University	Anthony LEVASSEUR et al
B.1.160.17	EPI_ISL_668504	Denmark/DCGC-9534/2020	2020/11/9	Albertsen Lab, Department of Chemistry and Bioscience, Aalborg University, Denmark	Danish Covid-19 Genome Consortium et al
B.1.160.18	EPI_ISL_730649	France/ARA-104848/2020	2020/11/10	CNR Virus des Infections Respiratoires - France SUD	Antonin Bal et al
B.1.160.19	EPI_ISL_2611008	Switzerland/LU-UHB-42555800/2020	2020/11/24	Clinical Bacteriology	Tim Roloff et al
B.1.160.20	EPI_ISL_737834	Switzerland/VS-ETHZ-320410/2020	2020/10/12	Department of Biosystems Science and Engineering, ETH Zürich	Chaoran Chen et al
B.1.160.21	EPI_ISL_744845	Luxembourg/LNS9084900/2020	2020/11/24	Laboratoire national de santé, Microbiology, Microbial Genomics Platform	Anke Wienecke-Baldacchino et al
B.1.160.22	EPI_ISL_603526	Switzerland/TI-ETHZ-310454/2020	2020/10/7	Department of Biosystems Science and Engineering, ETH Zürich	Christian Beisel et al
B.1.160.23	EPI_ISL_566530	England/MILK-9C297F/2020	2020/9/7	Wellcome Sanger Institute for the COVID-19 Genomics UK (COG-UK) consortium	The Lighthouse Lab in Milton Keynes et al

B.1.160.24	EPI_ISL_711390	Denmark/DCGC-14903/2020	2020/11/23	Albertsen Lab, Department of Chemistry and Bioscience, Aalborg University, Denmark	Danish Covid-19 Genome Consortium et al
B.1.160.25	EPI_ISL_918554	Brazil/AP-IEC-177390/2020	2020/12/16	Evandro Chagas Institute	Santos et al
B.1.160.26	EPI_ISL_603330	Switzerland/VD-ETHZ-300298/2020	2020/9/25	Department of Biosystems Science and Engineering, ETH Zürich	Christian Beisel et al
B.1.160.27	EPI_ISL_855362	Mayotte/IPP00169/2021	2021/1/5	National Reference Center for Viruses of Respiratory Infections, Institut Pasteur, Paris	Marion Barbet et al
B.1.160.28	EPI_ISL_707715	Belgium/ULG-10947/2020	2020/9/5	GIGA Medical Genomics	Keith Durkin et al
B.1.160.29	EPI_ISL_721807	Switzerland/GE-ETHZ-340094/2020	2020/10/26	Department of Biosystems Science and Engineering, ETH Zürich	Christian Beisel et al
B.1.160.30	EPI_ISL_537141	England/QEUH-95DF1D/2020	2020/8/7	Wellcome Sanger Institute for the COVID-19 Genomics UK (COG-UK) consortium	Harper VanSteenhouse et al
B.1.160.30	EPI_ISL_1140271	Germany/NW-RKI-I-010146/2020	2020/9/25	Robert Koch Institute	?
B.1.160.31	EPI_ISL_603484	Switzerland/SZ-ETHZ-310410/2020	2020/10/8	Department of Biosystems Science and Engineering, ETH Zürich	Christian Beisel et al
B.1.160.32	EPI_ISL_2596349	France/PAC-IHU-11568_Illu1/2020	2020/4/22	MEPHI, Aix Marseille University	Anthony LEVASSEUR et al
B.1.160.33	EPI_ISL_5314801	France/ARA-HCL990000125202/2020	2020/10/26	CNR Virus des Infections Respiratoires - France SUD	Antonin Bal et al
B.1.160.7	EPI_ISL_595857	Wales/QEUH-A31057/2020	2020/10/3	COVID-19 Genomics UK (COG-UK) Consortium	Dave J. Baker et al
B.1.160.8	EPI_ISL_619449	Denmark/DCGC-6549/2020	2020/10/5	Albertsen lab, Department of Chemistry and Bioscience, Aalborg University, Denmark	Danish Covid-19 Genome Consortia et al
B.1.160.9	EPI_ISL_1496478	Switzerland/BL-ETHZ-330467/2020	2020/10/19	Department of Biosystems Science and Engineering, ETH Zürich	Christian Beisel et al
B.1.161	EPI_ISL_482455	USA/OR-PROV-220/2020	2020/3/27	Providence St. Joseph Health Molecular Genomics Laboratory	Alexa K Dowdell et al
B.1.162	EPI_ISL_444599	USA/IL-NM0116/2020	2020/3/19	Ozer Lab	Ramon Lorenzo-Redondo et al
B.1.163	EPI_ISL_468389	USA/CA-CZB-1414/2020	2020/3/16	Chan-Zuckerberg Biohub	CZB Cliahub Consortium et al
B.1.164	EPI_ISL_456565	Australia/VIC1664/2020	2020/5/10	Microbiological Diagnostic Unit Public Health Laboratory and Victorian Infectious Diseases Reference Laboratory, Doherty Institute	Caly L. et al

B.1.165	EPI_ISL_458367	England/BRIS-12ACFC/2020	2020/5/1	Wellcome Sanger Institute for the COVID-19 Genomics UK (COG-UK) consortium	Stephanie Hutchings et al
B.1.166	EPI_ISL_483194	USA/CA-ALSR-0515-SAN/2020	2020/3/4	Andersen lab at Scripps Research	SEARCH Alliance San Diego with David Pride et al
B.1.167	EPI_ISL_443943	England/BRIS-1253DE/2020	2020/3/27	Wellcome Sanger Institute for the COVID-19 Genomics UK (COG-UK) consortium	Stephanie Hutchings et al
B.1.168	EPI_ISL_453122	Scotland/EDB4335/2020	2020/4/23	COVID-19 Genomics UK (COG-UK) Consortium	McHugh M et al
B.1.169	EPI_ISL_570230	USA/WA-UW-9195/2020	2020/5/15	UW Virology Lab	Pavitra Roychoudhury et al
B.1.170	EPI_ISL_603907	USA/CA-QDX-1625/2020	2020/3/19	Quest Diagnostics	Rosenthal et al
B.1.173	EPI_ISL_537248	England/SHEF-10B2E79/2020	2020/5/16	Wellcome Sanger Institute for the COVID-19 Genomics UK (COG-UK) consortium	Thushan de Silva et al
B.1.177	EPI_ISL_4899898	Morocco/INH-107/2020	2020/2/2	Functional Genomic Platform UATRS-biology, CNRST	Mohamed Rhajaoui et al
B.1.177	EPI_ISL_3054039	France/PAC-IHU-15335-N1/2021	2020/7/29	MEPHI, Aix Marseille University	Anthony LEVASSEUR et al
B.1.177	EPI_ISL_2017564	Spain/IB-HUSE-00072/2020	2020/9/26	HOSPITAL UNIVERSITARIO SON ESPASES	Carla López-Causapé et al
B.1.177.10	EPI_ISL_590540	England/QEUH-9B7746/2020	2020/9/2	Wellcome Sanger Institute for the COVID-19 Genomics UK (COG-UK) consortium	Harper VanSteenhouse et al
B.1.177.11	EPI_ISL_760151	SouthKorea/KDCA0367/2020	2020/4/4	Division of Emerging Infectious Diseases, Bureau of Infectious Diseases Diagnosis Control, Korea Disease Control and Prevention Agency	Ae Kyung Park et al
B.1.177.12	EPI_ISL_671267	Denmark/DCGC-12020/2020	2020/3/30	Albertsen Lab, Department of Chemistry and Bioscience, Aalborg University, Denmark	Danish Covid-19 Genome Consortium et al
B.1.177.14	EPI_ISL_4533657	Spain/UN-ORC01386/2020	2020/7/27	IAME UMR1137 Inserm, Université de Paris, Hôpital Bichat	Antoine Bridier-Nahmias et al
B.1.177.15	EPI_ISL_560081	Wales/MILK-9A89BB/2020	2020/9/1	COVID-19 Genomics UK (COG-UK) Consortium	Tanya Golubchik et al
B.1.177.16	EPI_ISL_567654	England/ALDP-9C5BB2/2020	2020/9/10	Wellcome Sanger Institute for the COVID-19 Genomics UK (COG-UK) consortium	Jacquelyn Wynn et al

B.1.177.17	EPI_ISL_590323	England/QEUH-9B4A13/2020	2020/8/2	Wellcome Sanger Institute for the COVID-19 Genomics UK (COG-UK) consortium	Harper VanSteenhouse et al
B.1.177.18	EPI_ISL_573138	England/CAMB-1B5F2A/2020	2020/9/14	COVID-19 Genomics UK (COG-UK) Consortium	Aminu S. Jahun et al
B.1.177.19	EPI_ISL_590415	England/QEUH-9B4E35/2020	2020/9/3	Wellcome Sanger Institute for the COVID-19 Genomics UK (COG-UK) consortium	Harper VanSteenhouse et al
B.1.177.2	EPI_ISL_590273	England/QEUH-9B4BC5/2020	2020/9/3	Wellcome Sanger Institute for the COVID-19 Genomics UK (COG-UK) consortium	Harper VanSteenhouse et al
B.1.177.20	EPI_ISL_611771	England/PORT-2D2111/2020	2020/3/21	COVID-19 Genomics UK (COG-UK) Consortium	Angela Beckett et al
B.1.177.21	EPI_ISL_616626	Denmark/DCGC-2791/2020	2020/8/24	Albertsen lab, Department of Chemistry and Bioscience, Aalborg University, Denmark	Danish Covid-19 Genome Consortia et al
B.1.177.23	EPI_ISL_2790847	France/IDF-IPP5558/2020	2020/4/8	National Reference Center for Viruses of Respiratory Infections, Institut Pasteur, Paris	Marion Barbet et al
B.1.177.24	EPI_ISL_616503	Denmark/DCGC-2624/2020	2020/8/17	Albertsen lab, Department of Chemistry and Bioscience, Aalborg University, Denmark	Danish Covid-19 Genome Consortia et al
B.1.177.25	EPI_ISL_613639	Gibraltar/204421176/2020	2020/10/27	Respiratory Virus Unit, Microbiology Services Colindale, Public Health England	PHE Covid Sequencing Team et al
B.1.177.26	EPI_ISL_595162	England/NORW-EFE2B/2020	2020/10/8	COVID-19 Genomics UK (COG-UK) Consortium	Dave J. Baker et al
B.1.177.27	EPI_ISL_620364	Denmark/DCGC-6464/2020	2020/10/12	Albertsen lab, Department of Chemistry and Bioscience, Aalborg University, Denmark	Danish Covid-19 Genome Consortia et al
B.1.177.28	EPI_ISL_614910	Switzerland/BE-ETHZ-330427/2020	2020/10/18	Department of Biosystems Science and Engineering, ETH Zürich	Christian Beisel et al
B.1.177.29	EPI_ISL_871914	Spain/CT-IBV-98013069/2020	2020/12/18	SeqCOVID-SPAIN consortium/IBV(CSIC)	Elisa Martró et al
B.1.177.3	EPI_ISL_552340	England/MILK-995201/2020	2020/8/24	Wellcome Sanger Institute for the COVID-19 Genomics UK (COG-UK) consortium	The Lighthouse Lab in Milton Keynes et al

B.1.177.30	EPI_ISL_649884	England/204393759/2020	2020/10/23	COVID-19 Genomics UK (COG-UK) Consortium	PHE Covid Sequencing Team et al
B.1.177.31	EPI_ISL_541841	England/QEUH-999100/2020	2020/8/24	Wellcome Sanger Institute for the COVID-19 Genomics UK (COG-UK) consortium	Harper VanSteenhouse et al
B.1.177.32	EPI_ISL_2323027	Spain/CL-IBV-99016864/2020	2020/9/29	SeqCOVID-SPAIN consortium/IBV(CSIC)	Ana Carvajal et al
B.1.177.33	EPI_ISL_837273	Italy/CAM-TIGEM-1017/2020	2020/9/2	TIGEM	Antonio Grimaldi et al
B.1.177.34	EPI_ISL_722792	Netherlands/ZH-EMC-1052/2020	2020/12/6	Erasmus Medical Center	Bas Oude Munnink et al
B.1.177.35	EPI_ISL_2099982	Spain/AN-IBV-99009691/2020	2020/9/26	SeqCOVID-SPAIN consortium/IBV(CSIC)	Federico García et al
B.1.177.36	EPI_ISL_1311034	Netherlands/NB-EMC-754/2020	2020/9/14	Erasmus Medical Center	Bas Oude Munnink et al
B.1.177.37	EPI_ISL_661244	Belgium/ULG-10821/2020	2020/11/16	GIGA Medical Genomics	Keith Durkin et al
B.1.177.38	EPI_ISL_567667	England/ALDP-9C5B3A/2020	2020/9/11	Wellcome Sanger Institute for the COVID-19 Genomics UK (COG-UK) consortium	Jacquelyn Wynn et al
B.1.177.39	EPI_ISL_1120921	Spain/MD-IBV-99020702/2020	2020/9/2	SeqCOVID-SPAIN consortium/IBV(CSIC)	Esther Viedma et al
B.1.177.4	EPI_ISL_950611	England/NOTT-246F3C/2020	2020/6/3	COVID-19 Genomics UK (COG-UK) Consortium	Gemma Clark et al
B.1.177.40	EPI_ISL_1819069	Spain/AN-IBV-97014065/2020	2020/8/15	SeqCOVID-SPAIN consortium/IBV(CSIC)	Inmaculada de Toro Peinado et al
B.1.177.41	EPI_ISL_635185	Norway/3621/2020	2020/9/20	Norwegian Institute of Public Health, Department of Virology	Kathrine Stene-Johansen et al
B.1.177.42	EPI_ISL_617404	Denmark/DCGC-8449/2020	2020/10/26	Albertsen lab, Department of Chemistry and Bioscience, Aalborg University, Denmark	Danish Covid-19 Genome Consortia et al
B.1.177.43	EPI_ISL_965734	Netherlands/LI-MUMC-166/2020	2020/8/17	Medical Microbiology, Maastricht University Medical Centre	Jozef Dingemans* et al
B.1.177.45	EPI_ISL_826922	Iceland/3230/2020	2020/9/12	deCODE genetics	Daniel F Gudbjartsson et al
B.1.177.46	EPI_ISL_648159	Sweden/20-53276/2020	2020/10/29	The Public Health Agency of Sweden	Anna-Malin Linde et al
B.1.177.47	EPI_ISL_724276	England/EXET-13DB08/2020	2020/12/1	COVID-19 Genomics UK (COG-UK) Consortium	Ben Temperton et al
B.1.177.48	EPI_ISL_580212	England/ALDP-9E9F16/2020	2020/9/24	Wellcome Sanger Institute for the COVID-19 Genomics UK (COG-UK) consortium	Jacquelyn Wynn et al

B.1.177.49	EPI_ISL_831148	Spain/MD-IBV-99013265/2020	2020/9/11	SeqCOVID-SPAIN consortium/IBV(CSIC)	María Rodríguez-Tejedor et al
B.1.177.5	EPI_ISL_540362	Scotland/QEUH-98F404/2020	2020/8/29	Wellcome Sanger Institute for the COVID-19 Genomics UK (COG-UK) consortium	Harper VanSteenhouse et al
B.1.177.50	EPI_ISL_539605	Germany/NW-HHU-89/2020	2020/8/17	Center of Medical Microbiology, Virology, and Hospital Hygiene, University of Duesseldorf	Maximilian Damagnez et al
B.1.177.51	EPI_ISL_3800826	Italy/LIG-111980-037-01/2020	2020/10/1	Istituto Zooprofilattico Sperimentale della Lombardia e dell'Emilia Romagna (IZSLER), Risk Analysis and Genomic Epidemiology Unit	Bianca Bruzzone et al
B.1.177.52	EPI_ISL_553019	England/MILK-9714F6/2020	2020/8/18	Wellcome Sanger Institute for the COVID-19 Genomics UK (COG-UK) consortium	The Lighthouse Lab in Milton Keynes et al
B.1.177.53	EPI_ISL_532369	England/QEUH-95FBF9/2020	2020/8/3	Wellcome Sanger Institute for the COVID-19 Genomics UK (COG-UK) consortium	Harper VanSteenhouse et al
B.1.177.54	EPI_ISL_578257	Ireland/D-NVRL-72IRL36222/2020	2020/8/17	National Virus Reference Laboratory	Michael Carr et al
B.1.177.55	EPI_ISL_573020	England/ALDP-9B661D/2020	2020/8/28	COVID-19 Genomics UK (COG-UK) Consortium	Tanya Golubchik et al
B.1.177.56	EPI_ISL_534365	Scotland/QEUH-964335/2020	2020/8/12	Wellcome Sanger Institute for the COVID-19 Genomics UK (COG-UK) consortium	Ana da Silva Filipe et al
B.1.177.57	EPI_ISL_950607	England/NOTT-246E4E/2020	2020/6/2	COVID-19 Genomics UK (COG-UK) Consortium	Gemma Clark et al
B.1.177.58	EPI_ISL_580665	England/MILK-9F0811/2020	2020/9/25	Wellcome Sanger Institute for the COVID-19 Genomics UK (COG-UK) consortium	The Lighthouse Lab in Milton Keynes et al
B.1.177.59	EPI_ISL_578262	Ireland/D-NVRL-73IRL25053/2020	2020/9/5	National Virus Reference Laboratory	Michael Carr et al
B.1.177.6	EPI_ISL_524856	USA/UT-UPHL-2008837/2020	2020/5/29	Utah Public Health Laboratory	Erin L. Young et al
B.1.177.60	EPI_ISL_534665	Scotland/QEUH-96A96A/2020	2020/8/17	Wellcome Sanger Institute for the COVID-19 Genomics UK (COG-UK) consortium	Ana da Silva Filipe et al
B.1.177.60	EPI_ISL_1312496	Latvia/985/2020	2020/9/28	Latvian Biomedical Research and Study Centre	Janis Pjalkovskis et al

B.1.177.62	EPI_ISL_2869783	Germany/NW-KRO-273/2020	2020/10/2	Center of Medical Microbiology, Virology, and Hospital Hygiene, University of Duesseldorf	Dennis Deschka et al
B.1.177.63	EPI_ISL_595582	Wales/PHWC-47ED20/2020	2020/10/13	COVID-19 Genomics UK (COG-UK) Consortium	Catherine Moore et al
B.1.177.64	EPI_ISL_818534	Denmark/DCGC-25502/2021	2021/1/4	Albertsen Lab, Department of Chemistry and Bioscience, Aalborg University, Denmark	Danish Covid-19 Genome Consortium et al
B.1.177.65	EPI_ISL_532427	Wales/QEUH-95E978/2020	2020/8/6	Wellcome Sanger Institute for the COVID-19 Genomics UK (COG-UK) consortium	Harper VanSteenhouse et al
B.1.177.66	EPI_ISL_768786	Ireland/D-NVRL-AIIDV1268v1/2020	2020/10/30	Irish Coronavirus Sequencing Consortium - National Virus Reference Laboratory	Michael Carr et al
B.1.177.67	EPI_ISL_752524	Ireland/MH-NVRL-77IRL72701/2020	2020/11/5	National Virus Reference Laboratory	Michael Carr et al
B.1.177.68	EPI_ISL_837373	Ireland/LK-NVRL-81IRL14907/2020	2020/12/27	National Virus Reference Laboratory	Michael Carr et al
B.1.177.69	EPI_ISL_599678	England/QEUH-9DCE55/2020	2020/9/21	Wellcome Sanger Institute for the COVID-19 Genomics UK (COG-UK) consortium	Harper VanSteenhouse et al
B.1.177.7	EPI_ISL_693277	Australia/QLD1276/2020	2020/3/21	Queensland Health Forensic and Scientific Services	Son Nguyen et al
B.1.177.70	EPI_ISL_669121	Denmark/DCGC-9709/2020	2020/11/9	Albertsen Lab, Department of Chemistry and Bioscience, Aalborg University, Denmark	Danish Covid-19 Genome Consortium et al
B.1.177.71	EPI_ISL_1296403	France/IDF_HB_112009081106/2020	2020/9/21	IAME UMR1137 Inserm, Université de Paris, Hôpital Bichat	Antoine Bridier-Nahmias et al
B.1.177.72	EPI_ISL_1119698	Switzerland/SO-ETHZ-501186/2020	2020/11/7	Department of Biosystems Science and Engineering, ETH Zürich	Chaoran Chen et al
B.1.177.73	EPI_ISL_1311716	Netherlands/ZH-EMC-1816/2020	2020/4/12	Erasmus Medical Center	Bas Oude Munnink et al
B.1.177.74	EPI_ISL_2458149	Spain/CT-HUVH-R85736/2020	2020/10/27	Hospital Universitari Vall d'Hebron - Vall d'Hebron Institut de Recerca	Cristina Andrés et al

B.1.177.75	EPI_ISL_1692830	Italy/CAM-TIGEM-IZSM-COLLI-9497/2020	2020/9/2	Telethon Institute of Genetics and Medicine (TIGEM)	Antonio Grimaldi Patrizia Annunziata Francesco Panariello Teresa Giuliano Michele Cennamo Valentina Bouche Chiara Colantuono Lucio Di Filippo Mariano Fiorenza Anna Manfredi Marcello Salvi Giuseppe Portella Andrea Ballabio Davide Cacchiarelli et al
B.1.177.76	EPI_ISL_722256	Spain/AR-IBV-98010614/2020	2020/10/1	SeqCOVID-SPAIN consortium/IBV(CSIC)	Antonio Rezusta López et al
B.1.177.77	EPI_ISL_3356319	Belgium/LHUB11590048/2020	2020/3/31	LHUB-ULB	Charlotte Michel et al
B.1.177.8	EPI_ISL_560031	Scotland/QEUH-9AF18F/2020	2020/9/3	COVID-19 Genomics UK (COG-UK) Consortium	Tanya Golubchik et al
B.1.177.80	EPI_ISL_661290	Sweden/20-53262/2020	2020/10/21	The Public Health Agency of Sweden	Department of Microbiology et al
B.1.177.81	EPI_ISL_1311761	Netherlands/NB-EMC-847/2020	2020/8/10	Erasmus Medical Center	Bas Oude Munnink et al
B.1.177.82	EPI_ISL_552201	England/MILK-99390E/2020	2020/8/26	Wellcome Sanger Institute for the COVID-19 Genomics UK (COG-UK) consortium	The Lighthouse Lab in Milton Keynes et al
B.1.177.83	EPI_ISL_547965	Italy/SAR-ATS-246/2020	2020/8/25	Laboratorio Specialistico UOC Ematologia Ospedale San Francesco - ATS ASSL NUORO	Piras Giovanna et al
B.1.177.84	EPI_ISL_566346	England/ALDP-9CDC5D/2020	2020/9/16	Wellcome Sanger Institute for the COVID-19 Genomics UK (COG-UK) consortium	Jacquelyn Wynn et al
B.1.177.85	EPI_ISL_740149	Luxembourg/LNS2593581/2020	2020/10/23	Laboratoire national de santé, Microbiology, Microbial Genomics Platform	Anke Wienecke-Baldacchino et al
B.1.177.86	EPI_ISL_621760	Denmark/DCGC-5222/2020	2020/9/21	Albertsen lab, Department of Chemistry and Bioscience, Aalborg University, Denmark	Danish Covid-19 Genome Consortia et al
B.1.177.87	EPI_ISL_552100	England/MILK-992EB5/2020	2020/8/26	Wellcome Sanger Institute for the COVID-19 Genomics UK (COG-UK) consortium	The Lighthouse Lab in Milton Keynes et al
B.1.177.88	EPI_ISL_837496	Italy/CAM-TIGEM-1344/2020	2020/12/26	TIGEM	Antonio Grimaldi et al

B.1.177.89	EPI_ISL_1658340	Switzerland/BE-ETHZ-570061/2020	2020/12/16	Department of Biosystems Science and Engineering, ETH Zurich	Christian Beisel et al
B.1.177.9	EPI_ISL_638633	England/QEUH-9AE33F/2020	2020/9/2	COVID-19 Genomics UK (COG-UK) Consortium	Aminu S. Jahun et al
B.1.178	EPI_ISL_417437	DRC/KN-0054/2020	2020/3/17	Pathogen Sequencing Lab, National Institute for Biomedical Research (INRB)	Placide Mbala-Kingebeni et al
B.1.179	EPI_ISL_614557	Denmark/DCGC-961/2020	2020/3/9	Albertsen lab, Department of Chemistry and Bioscience, Aalborg University, Denmark	Danish Covid-19 Genome Consortia et al
B.1.180	EPI_ISL_1501744	USA/IL-LCH-0052/2020	2020/4/6	Northwestern University - Ozer Lab	Ramon Lorenzo-Redondo et al
B.1.181	EPI_ISL_482362	USA/OR-PROV-091/2020	2020/4/20	Providence St. Joseph Health Molecular Genomics Laboratory	Alexa K Dowdell et al
B.1.182	EPI_ISL_509724	USA/FL-BPHL-0666/2020	2020/3/13	Florida Bureau of Public Health Laboratories	Sarah Schmedes et al
B.1.184	EPI_ISL_524739	India/GJ-GBRC-380a/2020	2020/6/18	Gujarat Biotechnology Research Centre	Pinal Trivedi et al
B.1.187	EPI_ISL_428927	Poland/PL_P4/2020	2020/3/28	ViroGenetics - BSL3 Laboratory of Virology; Human Genome Variation Research Group & Genomics Centre MCB; Bioinformatics Research Group	Wojciech Branicki et al
B.1.188	EPI_ISL_509203	USA/OR-OHSU-1447/2020	2020/7/17	Oregon SARS-CoV-2 Genome Sequencing Center	Brendan L. O'Connell et al
B.1.189	EPI_ISL_837604	Mexico/CMX-INER-0051/2020	2020/4/14	Instituto Nacional de Enfermedades Respiratorias (INER)	Celia Boukadida et al
B.1.190	EPI_ISL_547557	Netherlands/ZH-RIVM-10050/2020	2020/3/13	National Institute for Public Health and the Environment (RIVM)	Adam Meijer et al
B.1.192	EPI_ISL_648326	EquatorialGuinea/3350/2020	2020/4/24	University Hospital Basel, Clinical Bacteriology	Carlos Cortes et al
B.1.194	EPI_ISL_545420	USA/TX-HMH-2603/2020	2020/6/4	Houston Methodist Hospital	S. Wesley Long et al
B.1.195	EPI_ISL_541340	Brazil/PR-FIOCRUZ-5617/2020	2020/3/17	Laboratory of Respiratory Viruses and Measles, Oswaldo Cruz Institute, FIOCRUZ	Paola Resende et al
B.1.198	EPI_ISL_440738	England/BRIS-122BAC/2020	2020/4/3	Wellcome Sanger Institute for the COVID-19 Genomics UK (COG-UK) consortium	Stephanie Hutchings et al
B.1.199	EPI_ISL_467811	USA/CA-QDX-117/2020	2020/3/16	Quest Diagnostics	Anderson et al
B.1.2	EPI_ISL_2835566	USA/CA-SEARCH-103149/2020	2020/1/21	Andersen lab at Scripps Research	SEARCH Alliance San Diego with Aaron Harding et al

B.1.201	EPI_ISL_464549	England/20109028804/2020	2020/3/6	Respiratory Virus Unit, Microbiology Services Colindale, Public Health England	PHE Covid Sequencing Team et al
B.1.203	EPI_ISL_527748	CostaRica/INC-0080/2020	2020/7/5	Inciensa, Instituto Costarricense de Investigación y Enseñanza en Nutrición y Salud	Francisco Duarte et al
B.1.205	EPI_ISL_482468	Peru/LIM-01-2/2020	2020/3/5	Laboratorio de Referencia Nacional de Biotecnologia y Biologia Molecular. Instituto Nacional de Salud Peru	Carlos Padilla Rojas et al
B.1.206	EPI_ISL_511903	India/WB-IK62/2020	2020/4/9	National Institute of Biomedical Genomics - DBT's PAN-INDIA 1000 SARS--CoV-2 RNA Genome Sequencing Consortium	Arindam Maitra et al
B.1.208	EPI_ISL_418414	France/ARA-09434/2020	2020/3/15	CNR Virus des Infections Respiratoires - France SUD	Antonin Bal et al
B.1.210	EPI_ISL_1055383	India/MH-ICMR-C300/2020	2020/3/10	Indian Council of Medical Research- National Institute of Virology, Microbial Containment Complex	Pragya D. Yadav et al
B.1.211	EPI_ISL_416749	France/ARA-532/2020	2020/3/4	CNR Virus des Infections Respiratoires - France SUD	Bal et al
B.1.212	EPI_ISL_414629	France/HDF-1870/2020	2020/3/3	National Reference Center for Viruses of Respiratory Infections, Institut Pasteur, Paris	Mélnie Albert et al
B.1.213	EPI_ISL_645071	Poland/PL_MCB_72/2020	2020/5/10	Human Genome Variation Research Group, Malopolska Centre of Biotechnology	Kowalski et al
B.1.214	EPI_ISL_437357	DRC/3070/2020	2020/4/18	Pathogen Sequencing Lab, National Institute for Biomedical Research (INRB)	Placide Mbala-Kingebeni et al
B.1.214.1	EPI_ISL_912354	Congo/Q001C088AF_2101-TM-r1-002/2020	2020/12/5	NGS Competence Center Tuebingen, Institut für Medizinische Mikrobiologie und Hygiene, Universitaetsklinikum Tübingen	Angel Angelov et al
B.1.214.3	EPI_ISL_1384313	Luxembourg/LNS7752025/2020	2020/12/14	Laboratoire national de sante, Microbiology, Microbial Genomics Platform	Anke Wienecke-Baldacchino et al
B.1.214.4	EPI_ISL_971814	Denmark/DCGC-40137/2021	2021/1/25	Aalborg University	Danish Covid-19 Genome Consortium et al
B.1.215	EPI_ISL_485853	USA/VA-DCLS-0552/2020	2020/3/14	Virginia DCLS	Virginia DCLS et al

B.1.218	EPI_ISL_618374	Denmark/DCGC-2112/2020	2020/7/20	Albertsen lab, Department of Chemistry and Bioscience, Aalborg University, Denmark	Danish Covid-19 Genome Consortia et al
B.1.219	EPI_ISL_536230	Canada/Qc-LSPQ-L00239009/2020	2020/3/22	Laboratoire de santé publique du Québec	Sandrine Moreira et al
B.1.22	EPI_ISL_523199	Netherlands/GE-EMC-217/2020	2020/3/5	Erasmus Medical Center	Bas Oude Munnink et al
B.1.22.1	EPI_ISL_636603	SintEustatius/BQ-RIVM-10373/2020	2020/9/7	National Institute for Public Health and the Environment (RIVM)	Adam Meijer et al
B.1.220	EPI_ISL_457974	Oman/RESP-20-2282/2020	2020/3/14	Oman-NIC	Samira Al-Maruqi et al
B.1.221	EPI_ISL_2631277	Poland/KCZ_1.73/2020	2020/1/14	Department of Infectious, Tropical Diseases and Immune Deficiency, Pomeranian Medical University in Szczecin, Szczecin, Poland	Karol Serwin et al
B.1.221	EPI_ISL_1225484	Panama/GMI-PA395551/2020	2020/6/24	Gorgas Memorial Laboratory of Health Studies	Yamilka Diaz et al
B.1.221.1	EPI_ISL_553089	England/MILK-970CFF/2020	2020/8/18	Wellcome Sanger Institute for the COVID-19 Genomics UK (COG-UK) consortium	The Lighthouse Lab in Milton Keynes et al
B.1.221.2	EPI_ISL_2448328	Germany/SL-SU-10446284/2020	2020/9/10	Epigenetics, Saarland University	Kathrin Kattler et al
B.1.221.3	EPI_ISL_615254	Denmark/DCGC-5323/2020	2020/9/14	Albertsen lab, Department of Chemistry and Bioscience, Aalborg University, Denmark	Danish Covid-19 Genome Consortia et al
B.1.221.4	EPI_ISL_744659	Luxembourg/LNS7948120/2020	2020/11/2	Laboratoire national de santé, Microbiology, Microbial Genomics Platform	Anke Wienecke-Baldacchino et al
B.1.222	EPI_ISL_779712	Italy/LOM-UniMI08/2020	2020/2/24	Laboratory of Infectious Diseases, Department of Biomedical and Clinical Sciences L. Sacco, University of Milan	Alessia Lai et al
B.1.223	EPI_ISL_418152	Wales/PHWC-241A9/2020	2020/3/15	Public Health Wales Microbiology Cardiff	Catherine Moore et al
B.1.224	EPI_ISL_511642	Portugal/PT1095/2020	2020/3/16	Instituto Nacional de Saude (INSA)	Borges et al
B.1.225	EPI_ISL_549484	England/NORW-EFADC/2020	2020/4/1	COVID-19 Genomics UK (COG-UK) Consortium	Dave J. Baker et al
B.1.227	EPI_ISL_831975	Sweden/20-23088/2020	2020/3/16	The Public Health Agency of Sweden	Department of Microbiology et al
B.1.229	EPI_ISL_1620714	Ireland/KY-NVRL-20G33897/2020	2020/3/19	Irish Coronavirus Sequencing Consortium - Teagasc Moorepark	Jose Sanchez-Morgado et al

B.1.23	EPI_ISL_794712	Australia/WA540/2020	2020/3/18	PathWest Laboratory Medicine WA Microbial Surveillance Unit	PathWest Laboratory Medicine WA Microbial Surveillance Unit et al
B.1.231	EPI_ISL_478763	England/OXON-AF6C5/2020	2020/4/8	COVID-19 Genomics UK (COG-UK) Consortium	Tanya Golubchik et al
B.1.232	EPI_ISL_487893	Scotland/EDB3155/2020	2020/4/6	Wellcome Sanger Institute for the COVID-19 Genomics UK (COG-UK) consortium	McHugh M et al
B.1.233	EPI_ISL_1017380	USA/OR-PROV-398/2020	2020/6/16	Providence St. Joseph Health Molecular Genomics Laboratory	Alexa K Dowdell et al
B.1.234	EPI_ISL_694614	USA/AZ-TG376210/2020	2020/6/15	TGen North	Jolene Bowers et al
B.1.235	EPI_ISL_553969	England/ALDP-9562B1/2020	2020/6/8	Wellcome Sanger Institute for the COVID-19 Genomics UK (COG-UK) consortium	The Lighthouse Lab in Alderley Park et al
B.1.237	EPI_ISL_700567	SouthAfrica/NHLS-UCT-GP-5183/2020	2020/4/24	NHLS/UCT	Arash Iranzadeh et al
B.1.238	EPI_ISL_533364	England/QEUH-544733/2020	2020/5/14	Wellcome Sanger Institute for the COVID-19 Genomics UK (COG-UK) consortium	Harper VanSteenhouse et al
B.1.239	EPI_ISL_2712642	Mexico/BCN-SEARCH-102575/2020	2020/4/9	Andersen lab at Scripps Research	SEARCH Alliance with Gisela Barrera Badillo et al
B.1.240	EPI_ISL_422555	USA/NY-PV09200/2020	2020/3/22	MSHS Pathogen Surveillance Program	Ana S. Gonzalez-Reiche et al
B.1.240	EPI_ISL_1760240	PuertoRico/PR-CDC-S298/2021	2021/1/19	Centers for Disease Control and Prevention, Dengue Branch	Gilberto A. Santiago et al
B.1.240.1	EPI_ISL_636514	Aruba/AW-RIVM-10388/2020	2020/8/4	National Institute for Public Health and the Environment (RIVM)	Adam Meijer et al
B.1.240.2	EPI_ISL_969220	Canada/BC-BCCDC-4087/2020	2020/8/24	BCCDC Public Health Laboratory	Prystajecky Natalie et al
B.1.241	EPI_ISL_2712687	Mexico/BCN-SEARCH-102621/2020	2020/4/15	Andersen lab at Scripps Research	SEARCH Alliance with Gisela Barrera Badillo et al
B.1.241	EPI_ISL_1383041	Spain/NC-CHN-01000218/2020	2020/3/24	Centro de Secuenciación NASERTIC	Carmen Ezpeleta Baquedano et al
B.1.242	EPI_ISL_3031179	Norway/2783/2020	2020/6/12	Norwegian Institute of Public Health, Department of Virology	Kathrine Stene-Johansen et al
B.1.243	EPI_ISL_494714	USA/CA-ALSR-1649/2020	2020/3/23	Andersen lab at Scripps Research	SEARCH Alliance San Diego with Tracy Basler et al
B.1.243.1	EPI_ISL_5628643	USA/AZ-ASU18869/2021	2021/1/18	Arizona State University	Ajeet Bains et al

B.1.245	EPI_ISL_475618	USA/CA-CSMC14/2020	2020/3/24	Cedars-Sinai Medical Center, Molecular Pathology Laboratory of Department of Pathology & Laboratory Medicine and Genomic Core	Wenjuan Zhang et al
B.1.247	EPI_ISL_444484	India/GJ-GBRC53/2020	2020/5/4	Gujarat Biotechnology Research Centre	Pranay Shah et al
B.1.248	EPI_ISL_489620	Scotland/GCVR-17176E/2020	2020/3/28	Wellcome Sanger Institute for the COVID-19 Genomics UK (COG-UK) consortium	Ana da Silva Filipe et al
B.1.250	EPI_ISL_425483	England/NOTT-10E001/2020	2020/3/16	COVID-19 Genomics UK (COG-UK) Consortium	Gemma Clark et al
B.1.251	EPI_ISL_559053	England/ALDP-52B8D5/2020	2020/6/1	Wellcome Sanger Institute for the COVID-19 Genomics UK (COG-UK) consortium	The Lighthouse Lab in Alderley Park et al
B.1.252	EPI_ISL_443908	England/BRIS-1254AE/2020	2020/3/27	Wellcome Sanger Institute for the COVID-19 Genomics UK (COG-UK) consortium	Stephanie Hutchings et al
B.1.254	EPI_ISL_442230	England/CAMB-7CEC9/2020	2020/4/6	Wellcome Sanger Institute for the COVID-19 Genomics UK (COG-UK) consortium	Luke W Meredith et al
B.1.256	EPI_ISL_489646	Scotland/GCVR-16FD16/2020	2020/3/22	Wellcome Sanger Institute for the COVID-19 Genomics UK (COG-UK) consortium	Ana da Silva Filipe et al
B.1.258	EPI_ISL_423656	England/20130069504/2020	2020/3/22	Respiratory Virus Unit, Microbiology Services Colindale, Public Health England	Monica Galiano et al
B.1.258.10	EPI_ISL_612125	Scotland/CVR4698/2020	2020/9/20	COVID-19 Genomics UK (COG-UK) Consortium	Ana da Silva Filipe et al
B.1.258.11	EPI_ISL_615212	Denmark/DCGC-5281/2020	2020/9/14	Albertsen lab, Department of Chemistry and Bioscience, Aalborg University, Denmark	Danish Covid-19 Genome Consortia et al
B.1.258.14	EPI_ISL_882747	Italy/CAM-CRGS-241/2020	2020/10/6	1. Genome Research Center for Health (CRGS) / 2. Laboratory of Molecular Medicine and Genomics(LMMGe) / 3. Center for Research in Pure and Applied Mathematics (CRMPA)	Giorgio Giurato et al
B.1.258.15	EPI_ISL_6230322	Denmark/DCGC-207339/2020	2020/11/10	Statens Serum Institut Bioinformatics and Microbial Genomics	Danish Covid-19 Genome Consortium et al

B.1.258.16	EPI_ISL_711874	Denmark/DCGC-14621/2020	2020/11/23	Albertsen Lab, Department of Chemistry and Bioscience, Aalborg University, Denmark	Danish Covid-19 Genome Consortium et al
B.1.258.17	EPI_ISL_1499000	Slovenia/77414/2020	2020/8/28	Institute of Microbiology and Immunology, Faculty of Medicine, University of Ljubljana	Alen Suljič et al
B.1.258.18	EPI_ISL_722425	Netherlands/ZH-EMC-1068/2020	2020/10/5	Erasmus Medical Center	Bas Oude Munnink et al
B.1.258.19	EPI_ISL_711361	Denmark/DCGC-14599/2020	2020/11/23	Albertsen Lab, Department of Chemistry and Bioscience, Aalborg University, Denmark	Danish Covid-19 Genome Consortium et al
B.1.258.2	EPI_ISL_558199	England/ALDP-672355/2020	2020/7/3	Wellcome Sanger Institute for the COVID-19 Genomics UK (COG-UK) consortium	The Lighthouse Lab in Alderley Park et al
B.1.258.20	EPI_ISL_815374	Germany/BY-Cento-32458760/2020	2020/9/23	Centogene	Peter Bauer et al
B.1.258.21	EPI_ISL_632445	Netherlands/FL-EMC-35/2020	2020/10/8	Erasmus Medical Center	Bas Oude Munnink et al
B.1.258.22	EPI_ISL_2898873	Sweden/01_SE100_21CS503818/2020	2020/12/7	Karolinska University Hospital	Jan Albert et al
B.1.258.23	EPI_ISL_849870	USA/UT-UPHL-2101980997/2020	2020/12/19	Utah Public Health Laboratory	Erin L. Young et al
B.1.258.24	EPI_ISL_804053	Italy/FVG-ASUGI-152122/2020	2020/12/21	ARGO Laboratorio Genomica ed Epigenomica	Licastro D et al
B.1.258.3	EPI_ISL_557984	England/MILK-622EBD/2020	2020/7/6	Wellcome Sanger Institute for the COVID-19 Genomics UK (COG-UK) consortium	The Lighthouse Lab in Milton Keynes et al
B.1.258.4	EPI_ISL_590381	England/QEUH-9B5520/2020	2020/9/3	Wellcome Sanger Institute for the COVID-19 Genomics UK (COG-UK) consortium	Harper VanSteenhouse et al
B.1.258.5	EPI_ISL_572850	England/ALDP-9BCEA6/2020	2020/9/9	COVID-19 Genomics UK (COG-UK) Consortium	Tanya Golubchik et al
B.1.258.6	EPI_ISL_567431	England/QEUH-9C9F2B/2020	2020/9/10	Wellcome Sanger Institute for the COVID-19 Genomics UK (COG-UK) consortium	Harper VanSteenhouse et al
B.1.258.7	EPI_ISL_549868	England/MILK-99D096/2020	2020/8/29	Wellcome Sanger Institute for the COVID-19 Genomics UK (COG-UK) consortium	The Lighthouse Lab in Milton Keynes et al

B.1.258.9	EPI_ISL_616087	Denmark/DCGC-3333/2020	2020/8/31	Albertsen lab, Department of Chemistry and Bioscience, Aalborg University, Denmark	Danish Covid-19 Genome Consortia et al
B.1.260	EPI_ISL_678037	SaudiArabia/KAUST-MADINAH665/2020	2020/3/26	Pathogen Genomics Lab King Abdullah University of Science and Technology(KAUST)	Sara Mfarrej et al
B.1.263	EPI_ISL_452032	Denmark/ALAB-HH-157/2020	2020/3/19	Albertsen lab, Department of Chemistry and Bioscience, Aalborg University, Denmark	Rasmus Kirkegaard et al
B.1.264	EPI_ISL_604309	USA/CA-QDX-2021/2020	2020/3/17	Quest Diagnostics	Rosenthal et al
B.1.264.1	EPI_ISL_733209	Russia/AST-RII-MH4485S/2020	2020/8/26	WHO National Influenza Centre Russian Federation	Andrey Komissarov et al
B.1.265	EPI_ISL_571780	USA/MN-QDX-981/2020	2020/3/15	Quest Diagnostics	Rosenthal et al
B.1.267	EPI_ISL_494727	USA/CA-ALSR-1667/2020	2020/3/24	Andersen lab at Scripps Research	SEARCH Alliance San Diego with Tracy Basler et al
B.1.268	EPI_ISL_444022	USA/TX_GCID_192000003/2020	2020/3/18	Baylor College of Medicine: HGSC	Vasanthi Avadhanula et al
B.1.270	EPI_ISL_586299	Canada/ON-S1222/2020	2020/3/18	McMaster University	Allison McGeer et al
B.1.273	EPI_ISL_940393	France/IDF_HB_112003050544/2020	2020/3/13	IAME UMR1137 Inserm, Université de Paris, Hôpital Bichat	Antoine Bridier-Nahmias et al
B.1.274	EPI_ISL_1001043	Cameroon/Yaounde-20V-6189/2020	2020/4/13	Institut Pasteur de Dakar	Njouom Richard et al
B.1.276	EPI_ISL_434852	USA/TX-HMH0153/2020	2020/3/22	Houston Methodist Hospital	S. Wesley Long et al
B.1.277	EPI_ISL_414637	France/HDF-1993/2020	2020/3/4	National Reference Center for Viruses of Respiratory Infections, Institut Pasteur, Paris	Mélnie Albert et al
B.1.280	EPI_ISL_512564	USA/FL-BPHL-0758/2020	2020/6/27	Florida Bureau of Public Health Laboratories	Sarah Schmedes et al
B.1.281	EPI_ISL_483563	Bahrain/BAH-22/2020	2020/4/8	Erasmus Medical Center	Bas Oude Munnink et al
B.1.282	EPI_ISL_558971	England/ALDP-52B87B/2020	2020/6/1	Wellcome Sanger Institute for the COVID-19 Genomics UK (COG-UK) consortium	The Lighthouse Lab in Alderley Park et al
B.1.284	EPI_ISL_420643	England/20136094704/2020	2020/3/26	Respiratory Virus Unit, Microbiology Services Colindale, Public Health England	Monica Galiano et al
B.1.284	EPI_ISL_604936	USA/LA-QDX-2624/2020	2020/3/16	Quest Diagnostics	Rosenthal et al
B.1.285	EPI_ISL_544039	USA/TX-HMH-3163/2020	2020/6/10	Houston Methodist Hospital	S. Wesley Long et al

B.1.287	EPI_ISL_569689	USA/TX-DSHS-0577/2020	2020/6/2	Texas Department of State Health Services	Rashmi Tuladhar et al
B.1.289	EPI_ISL_914632	USA/AZ-TG655061/2020	2020/3/29	TGen North	"Jolene Bowers et al
B.1.291	EPI_ISL_1517403	CostaRica/HNN-0359/2020	2020/5/31	Inciensa, Instituto Costarricense de Investigación y Enseñanza en Nutrición y Salud	Cristian Peréz-Corrales et al
B.1.292	EPI_ISL_427490	USA/NY-NYUMC195/2020	2020/4/1	Departments of Pathology and Medicine, New York University School of Medicine	Maria Aguero-Rosenfeld et al
B.1.293	EPI_ISL_603938	USA/FL-QDX-1620/2020	2020/3/16	Quest Diagnostics	Rosenthal et al
B.1.294	EPI_ISL_484840	USA/WI-UW-465/2020	2020/6/16	University of Wisconsin-Madison AIDS Vaccine Research Laboratories	Gage Moreno et al
B.1.298	EPI_ISL_676923	USA/NY-Wadsworth-96601-01/2020	2020/3/20	Wadsworth Center, New York State Department.of Health	Kirsten St. George et al
B.1.3	EPI_ISL_4025997	USA/IL-NM-8510/2020	2020/4/18	Northwestern University - Center for Pathogen Genomics and Microbial Evolution	Ramon Lorenzo-Redondo et al
B.1.301	EPI_ISL_493063	USA/WY-WYPHL-20007007/2020	2020/4/1	Wyoming Public Health Laboratory	Noah Hull et al
B.1.302	EPI_ISL_631571	USA/NY-NYCPHL-000078/2020	2020/3/12	New York City Public Health Laboratory	Jade Wang et al
B.1.304	EPI_ISL_484886	USA/WI-UW-511/2020	2020/6/5	University of Wisconsin-Madison AIDS Vaccine Research Laboratories	Gage Moreno et al
B.1.305	EPI_ISL_1321842	USA/TX-DSHS-4209/2020	2020/6/9	TXDSHS	Rashmi Tuladhar et al
B.1.306	EPI_ISL_594313	USA/FL-BPHL-1623/2020	2020/6/3	Florida Bureau of Public Health Laboratories	Sarah Schmedes et al
B.1.308	EPI_ISL_805923	Canada/AB-35896/2020	2020/3/22	Alberta Precision Labs (APL)	Gordon P et al
B.1.309	EPI_ISL_576529	USA/CA-IGI-0340/2020	2020/6/29	Innovative Genomics Institute, UC Berkeley	Stacia Wyman et al
B.1.310	EPI_ISL_447404	Israel/2046434/2020	2020/4/1	Stern Lab	Stern Lab et al
B.1.311	EPI_ISL_578151	USA/MI-UM-10033882288/2020	2020/4/8	Lauring Lab, University of Michigan, Department of Microbiology and Immunology	Valesano et al
B.1.313	EPI_ISL_696019	USA/AZ-TG654719/2020	2020/4/1	TGen North	Jolene Bowers et al
B.1.314	EPI_ISL_421354	USA/NY-PV08125/2020	2020/3/13	MSHS Pathogen Surveillance Program	Ana S. Gonzalez-Reiche et al
B.1.315	EPI_ISL_428202	USA/NE-875/2020	2020/3/25	UNMC COVID-19 Response Team	UNMC COVID-19 Response Team et al
B.1.316	EPI_ISL_586325	Canada/ON-S1274/2020	2020/4/29	McMaster University	Allison McGeer et al

B.1.318	EPI_ISL_514188	USA/FL-BPHL-0842/2020	2020/6/22	Florida Bureau of Public Health Laboratories	Sarah Schmedes et al
B.1.319	EPI_ISL_482469	Canada/NS_40/2020	2020/3/5	National Microbiology Laboratory	Anna Majer et al
B.1.320	EPI_ISL_424890	USA/UT-CDC-03068786-001/2020	2020/3/7	Pathogen Discovery, Respiratory Viruses Branch, Division of Viral Diseases, Centers for Disease Control and Prevention	Yan Li et al
B.1.321	EPI_ISL_418985	Belgium/LT-030956/2020	2020/3/9	KU Leuven, Clinical and Epidemiological Virology	Bert Vanmechelen et al
B.1.323	EPI_ISL_450111	USA/NY-PV09434/2020	2020/3/13	MSHS Pathogen Surveillance Program	Ana S. Gonzalez-Reiche et al
B.1.324	EPI_ISL_467906	USA/TX-QDX-25/2020	2020/3/15	Quest Diagnostics	Anderson et al
B.1.325	EPI_ISL_545312	USA/TX-HMH-2445/2020	2020/6/1	Houston Methodist Hospital	S. Wesley Long et al
B.1.326	EPI_ISL_542539	USA/TX-HMH-1113/2020	2020/4/25	Houston Methodist Hospital	S. Wesley Long et al
B.1.328	EPI_ISL_509478	USA/MD-MDH-0074/2020	2020/5/23	Maryland Department of Health	Keller et al
B.1.329	EPI_ISL_2869924	Germany/NW-KRO-463/2020	2020/4/9	Center of Medical Microbiology, Virology, and Hospital Hygiene, University of Duesseldorf	Dennis Deschka et al
B.1.330	EPI_ISL_483013	USA/MN-MDH-1293/2020	2020/3/24	Minnesota Department of Health, Public Health Laboratory	Matt Plumb et al
B.1.332	EPI_ISL_632050	USA/NY-NYCPHL-000592/2020	2020/3/10	New York City Public Health Laboratory	Jade Wang et al
B.1.333	EPI_ISL_604460	USA/MI-QDX-2087/2020	2020/3/17	Quest Diagnostics	Rosenthal et al
B.1.334	EPI_ISL_632146	USA/NY-NYCPHL-000766/2020	2020/3/20	New York City Public Health Laboratory	Jade Wang et al
B.1.335	EPI_ISL_467407	USA/NY-NYUMC914/2020	2020/5/8	Departments of Pathology and Medicine, New York University School of Medicine	Maria Aguero-Rosenfeld et al
B.1.336	EPI_ISL_468562	USA/CA-QDX-110/2020	2020/3/13	Quest Diagnostics	Anderson et al
B.1.337	EPI_ISL_2727987	USA/WY-WYPHL-20037947/2020	2020/7/5	Wyoming Public Health Laboratory	Jim Mildenberger et al
B.1.338	EPI_ISL_604872	USA/WA-QDX-2548/2020	2020/3/16	Quest Diagnostics	Rosenthal et al
B.1.340	EPI_ISL_3726054	USA/TN-VUMC-000080/2020	2020/3/13	Dr. Suman Das Lab - Vanderbilt University Medical Center (VUMC) (https://my.vanderbilt.edu/daslab/)	Grant Vestal et al
B.1.341	EPI_ISL_570200	USA/WA-UW-8165/2020	2020/4/25	UW Virology Lab	Pavitra Roychoudhury et al
B.1.342	EPI_ISL_510834	Sweden/20-51697/2020	2020/5/23	The Public Health Agency of Sweden	Oskar Karlsson Lindsjo et al
B.1.343	EPI_ISL_429376	Denmark/ALAB-SSI160/2020	2020/3/9	Albertsen lab, Department of Chemistry and Bioscience, Aalborg University, Denmark	Rasmus Kirkegaard et al

B.1.344	EPI_ISL_2549656	USA/CO-CDPHE-2003120688/2020	2020/3/12	Colorado Department of Public Health and Environment	Laura Bankers et al
B.1.346	EPI_ISL_1973129	Japan/Donner270/2020	2020/4/13	Keio University School of Medicine	Kenjiro Kosaki et al
B.1.349	EPI_ISL_1241045	Canada/QC-JEW-Q8292157/2020	2020/4/29	Laboratoire de santé publique du Québec	Sandrine Moreira et al
B.1.35	EPI_ISL_514566	Wales/PHWC-169A15/2020	2020/3/20	COVID-19 Genomics UK (COG-UK) Consortium	Catherine Moore et al
B.1.350	EPI_ISL_452327	Beijing/DT-travelAT01/2020	2020/3/13	Laboratory of Infectious Diseases Center of Beijing Ditan Hospital	Siyuan Yang et al
B.1.350.1	EPI_ISL_512433	England/PORT-2F5DE7/2020	2020/5/25	COVID-19 Genomics UK (COG-UK) Consortium	Angela Beckett et al
B.1.351	EPI_ISL_2493065	Zimbabwe/CERI-KRISP-K011662/2020	2020/5/27	CERI, Centre for Epidemic Response and Innvoation, Stellenbosch University and KRISP, KZN Research Innovation and Sequencing Platform, UKZN.	Air Comodor Dr J. Chimedza et al
B.1.351.2	EPI_ISL_736966	SouthAfrica/KRISP-MDSH920866/2020	2020/12/6	KRISP, KZN Research Innovation and Sequencing Platform	Giandhari J et al
B.1.351.3	EPI_ISL_943561	Bangladesh/BCSIR-NILMRC-448/2021	2021/1/24	Genomic Research Lab, BCSIR	Tanjina Akhtar Banu et al
B.1.351.5	EPI_ISL_1700678	EquatorialGuinea/81769/2021	2021/1/14	Swiss Tropical and Public Health Institute	Salome Hosch et al
B.1.354	EPI_ISL_418228	France/HDF-2405/2020	2020/3/12	National Reference Center for Viruses of Respiratory Infections, Institut Pasteur, Paris	Mélanie Albert et al
B.1.355	EPI_ISL_449800	Chile/RM-ISPCH-53/2020	2020/4/1	Instituto de Salud Publica de Chile	Andrés E Castillo et al
B.1.356	EPI_ISL_418206	Senegal/003/2020	2020/2/28	Institut Pasteur de Dakar	Ndongo Dia et al
B.1.357	EPI_ISL_1017469	USA/OR-PROV-535/2020	2020/5/21	Providence St. Joseph Health Molecular Genomics Laboratory	Alexa K Dowdell et al
B.1.358	EPI_ISL_437630	Denmark/ALAB-SSI-1046/2020	2020/4/1	Albertsen lab, Department of Chemistry and Bioscience, Aalborg University, Denmark	Rasmus Kirkegaard et al
B.1.359	EPI_ISL_525612	USA/NY-Wadsworth-14422-01/2020	2020/3/9	Wadsworth Center, New York State Department of Health	Kirsten St. George et al
B.1.36	EPI_ISL_490003	SaudiArabia/562/2020	2020/2/16	King Fahad Medical City	Alosaimi et al
B.1.36.1	EPI_ISL_490042	Australia/NSW633/2020	2020/7/1	NSW Health Pathology - Institute of Clinical Pathology and Medical Research; Westmead Hospital; University of Sydney	CIDM-PH et al.

B.1.36.10	EPI_ISL_513168	SaudiArabia/KAUST-MADINAH599/2020	2020/4/30	Pathogen Genomics Lab King Abdullah University of Science and Technology(KAUST)	Sara Mfarrej et al
B.1.36.12	EPI_ISL_558361	England/MILK-566C5A/2020	2020/6/12	Wellcome Sanger Institute for the COVID-19 Genomics UK (COG-UK) consortium	The Lighthouse Lab in Milton Keynes et al
B.1.36.16	EPI_ISL_450344	Bangladesh/BARJ-CVASU-CTG-517/2020	2020/5/3	Basic and Applied Research on Jute Project	Rasel Ahmed et al
B.1.36.17	EPI_ISL_533016	Scotland/QEUH-789563/2020	2020/7/16	Wellcome Sanger Institute for the COVID-19 Genomics UK (COG-UK) consortium	Ana da Silva Filipe et al
B.1.36.18	EPI_ISL_3276881	India/DL-ILBS-17864/2020	2020/6/5	ILBS	Ekta Gupta et al
B.1.36.19	EPI_ISL_956311	Indonesia/JI-ITDua-17047NTv/2020	2020/7/16	Institute of Tropical Disease, Universitas Airlangga	Kazufumi Shimizu et al
B.1.36.2	EPI_ISL_638446	England/CAMC-A65DDB/2020	2020/10/11	COVID-19 Genomics UK (COG-UK) Consortium	Aminu S. Jahun et al
B.1.36.20	EPI_ISL_534634	Scotland/QEUH-96D396/2020	2020/8/20	Wellcome Sanger Institute for the COVID-19 Genomics UK (COG-UK) Consortium	Ana da Silva Filipe et al
B.1.36.21	EPI_ISL_775318	Norway/4694/2020	2020/10/7	Norwegian Institute of Public Health, Department of Virology	Kathrine Stene-Johansen et al
B.1.36.22	EPI_ISL_1586054	Iceland/926/2020	2020/4/14	deCODE genetics	Daniel F Gudbjartsson et al
B.1.36.23	EPI_ISL_4482150	Netherlands/ZH-EMC-3480/2020	2020/5/8	Erasmus Medical Center	Bas Oude Munnink et al
B.1.36.24	EPI_ISL_1751685	Germany/BY-MVP-000002132/2020	2020/10/27	Laboratory for Functional Genome Analysis; Dept. Genomics; Gene Center of the LMU Munich	Max Muenchhoff et al
B.1.36.25	EPI_ISL_618931	Denmark/DCGC-7478/2020	2020/10/12	Albertsen lab, Department of Chemistry and Bioscience, Aalborg University, Denmark	Danish Covid-19 Genome Consortia et al
B.1.36.26	EPI_ISL_876599	Canada/ON-SC0258/2020	2020/8/26	McMaster University	Allison McGeer et al
B.1.36.27	EPI_ISL_760032	HongKong/VM20123554/2020	2020/9/18	School of Public Health, The University of Hong Kong	Daniel Chu et al
B.1.36.28	EPI_ISL_566961	England/MILK-9C3E9D/2020	2020/9/9	Wellcome Sanger Institute for the COVID-19 Genomics UK (COG-UK) consortium	The Lighthouse Lab in Milton Keynes et al
B.1.36.29	EPI_ISL_862465	India/AP-CS0392/2020	2020/6/27	CSIR Institute of Genomics and Integrative Biology	Pallavali Roja Rani et al

B.1.36.31	EPI_ISL_1229177	Australia/SA0633/2020	2020/3/8	SA Pathology	Lex Leong et al
B.1.36.33	EPI_ISL_596752	Australia/WA189/2020	2020/5/23	PathWest Laboratory Medicine WA Microbial Surveillance Unit	PathWest Laboratory Medicine WA Microbial Surveillance Unit et al
B.1.36.34	EPI_ISL_569969	Canada/ON-UHTC-0199/2020	2020/6/10	Ontario Institute for Cancer Research	Ramzi Fattouh et al
B.1.36.35	EPI_ISL_1009016	Lebanon/LAU-R87/2020	2020/9/1	School of Pharmacy	Ahmed Kandeil et al
B.1.36.36	EPI_ISL_483663	Switzerland/GE-ETHZ-150035/2020	2020/6/3	Department of Biosystems Science and Engineering, ETH Zürich	Christian Beisel et al
B.1.36.37	EPI_ISL_4421463	Canada/BC-BCCDC-35581/2020	2020/10/6	BCCDC Public Health Laboratory	Prystajecky Natalie et al
B.1.36.38	EPI_ISL_1109628	Egypt/MASRI-C5-013/2020	2020/6/8	Medical Ain Shams Research Institute (MASRI), Ain Shams University	Hesham Elghazaly et al
B.1.36.39	EPI_ISL_584847	England/QEUH-9E3495/2020	2020/9/21	COVID-19 Genomics UK (COG-UK) Consortium	Darren L Smith et al
B.1.36.7	EPI_ISL_1014687	Iran/Tehran-055M/2020	2020/8/17	National Influenza Center	J Yavarian et al
B.1.36.8	EPI_ISL_450783	India/GJ-GBRC95b/2020	2020/4/11	Gujarat Biotechnology Research Centre	Shirish Patel et al
B.1.36.9	EPI_ISL_513222	SaudiArabia/KAUST-MAKKAH301/2020	2020/4/21	Pathogen Genomics Lab King Abdullah University of Science and Technology(KAUST)	Sharif Hala et al
B.1.360	EPI_ISL_437943	Austria/CeMM0074/2020	2020/3/17	Bergthaler laboratory, CeMM Research Center for Molecular Medicine of the Austrian Academy of Sciences	Alexandra Popa et al
B.1.361	EPI_ISL_602312	USA/FL-B2TM/2020	2020/8/2	University of Miami Immunology and Histocompatibility Laboratory	Emilio Margolles-Clark et al
B.1.362	EPI_ISL_447427	Israel/13077723/2020	2020/4/3	Stern Lab	Stern Lab et al
B.1.362.1	EPI_ISL_601611	England/CAMC-9D2095/2020	2020/9/19	Wellcome Sanger Institute for the COVID-19 Genomics UK (COG-UK) consortium	Rob Howes et al
B.1.362.2	EPI_ISL_575332	Israel/CVL-s2049/2020	2020/9/1	Israel Central Virology laboratory	Neta Zuckerman et al
B.1.363	EPI_ISL_3009337	USA/AZ-TG968075/2020	2020/9/3	TGen North	Jolene Bowers et al
B.1.366	EPI_ISL_494543	USA/TX-QDX-188/2020	2020/4/27	Quest Diagnostics	Anderson et al
B.1.367	EPI_ISL_510700	Switzerland/GE-ETHZ-200011/2020	2020/7/13	Department of Biosystems Science and Engineering, ETH Zürich	Christian Beisel et al
B.1.369	EPI_ISL_1591225	USA/NE-CDC-3960290-001/2020	2020/3/11	Pathogen Discovery, Respiratory Viruses Branch, Division of Viral Diseases, Centers for Disease Control and Prevention	Yan Li et al

B.1.369.1	EPI_ISL_961578	Canada/NB-NML-3272/2020	2020/12/29	National Microbiology Laboratory (NML)	Anna Majer et al
B.1.37	EPI_ISL_452112	USA/TX-CDC-03070108-001/2020	2020/3/10	Pathogen Discovery, Respiratory Viruses Branch, Division of Viral Diseases, Centers for Disease Control and Prevention	Yan Li et al
B.1.370	EPI_ISL_417971	USA/UT-028/2020	2020/3/19	Utah Public Health Laboratory	Erin Young et al
B.1.371	EPI_ISL_414616	USA/WA-UW29/2020	2020/3/8	UW Virology Lab	Pavitra Roychoudhury et al
B.1.372	EPI_ISL_622596	Denmark/DCGC-1846/2020	2020/3/30	Albertsen lab, Department of Chemistry and Bioscience, Aalborg University, Denmark	Danish Covid-19 Genome Consortia et al
B.1.375	EPI_ISL_976921	USA/MA-Broad_UMMS-00165/2020	2020/9/14	Infectious Disease Program, Broad Institute of Harvard and MIT	Lemieux et al
B.1.377	EPI_ISL_1392875	USA/MO-MSPHL-000515/2020	2020/4/19	Missouri State Public Health Laboratory	Matthew Sinn et al
B.1.378	EPI_ISL_571600	USA/IL-QDX-831/2020	2020/3/14	Quest Diagnostics	Rosenthal et al
B.1.379	EPI_ISL_440220	England/BRIS-12193A/2020	2020/3/16	Wellcome Sanger Institute for the COVID-19 Genomics UK (COG-UK) consortium	Stephanie Hutchings et al
B.1.38	EPI_ISL_418954	USA/WA-UW389/2020	2020/3/17	UW Virology Lab	Pavitra Roychoudhury et al
B.1.380	EPI_ISL_960302	Rwanda/NRLNAT2054/2020	2020/5/27	GIGA Medical Genomics	Yvan Butera et al
B.1.381	EPI_ISL_3068006	SouthAfrica/Tygerberg_60/2020	2020/4/14	Division of Medical Virology, Stellenbosch University and NHLS Tygerberg Hospital	Susan Engelbrecht et al
B.1.382	EPI_ISL_590777	USA/MN-MDH-1774/2020	2020/4/13	Minnesota Department of Health, Public Health Laboratory	Matt Plumb et al
B.1.383	EPI_ISL_413588	Netherlands/Utrecht_1363564/2020	2020/3/1	Erasmus Medical Center	David Nieuwenhuijse et al
B.1.384	EPI_ISL_509587	USA/UT-03775/2020	2020/4/1	Utah Public Health Laboratory	Heidi Butz et al
B.1.385	EPI_ISL_676618	USA/TX-DSHS-1485/2020	2020/6/22	Texas Department of State Health Services	Rashmi Tuladhar et al
B.1.387	EPI_ISL_416756	France/ARA-06531/2020	2020/3/6	CNR Virus des Infections Respiratoires - France SUD	Bal et al
B.1.388	EPI_ISL_614355	Coted-Ivoire/BKE0125/2020	2020/6/10	Project group Epidemology of Highly Pathogenic Microorganisms, Robert Koch-Institute	Chantal Akoua-Koffi et al

B.1.389	EPI_ISL_618138	Denmark/DCGC-1568/2020	2020/6/15	Albertsen lab, Department of Chemistry and Bioscience, Aalborg University, Denmark	Danish Covid-19 Genome Consortia et al
B.1.39	EPI_ISL_614577	Denmark/DCGC-979/2020	2020/3/9	Albertsen lab, Department of Chemistry and Bioscience, Aalborg University, Denmark	Danish Covid-19 Genome Consortia et al
B.1.390	EPI_ISL_460122	USA/MA-MGH-00080/2020	2020/3/25	Infectious Disease Program, Broad Institute of Harvard and MIT	Lemieux et al
B.1.391	EPI_ISL_464737	England/201140585/2020	2020/3/10	Respiratory Virus Unit, Microbiology Services Colindale, Public Health England	PHE Covid Sequencing Team et al
B.1.393	EPI_ISL_2779421	Kenya/BUS-406/2020	2020/5/29	USAMRD-A, Basic Science Laboratory	Gathii Kimita et al
B.1.395	EPI_ISL_415435	Wales/PHW06/2020	2020/3/6	Public Health Wales Microbiology Cardiff	Catherine Moore et al
B.1.396	EPI_ISL_2596864	France/PAC-IHU-10794_Illu1/2020	2020/4/6	MEPHI, Aix Marseille University	Anthony LEVASSEUR et al
B.1.397	EPI_ISL_2712631	Mexico/BCN-SEARCH-102564/2020	2020/4/7	Andersen lab at Scripps Research	SEARCH Alliance with Gisela Barrera Badillo et al
B.1.398	EPI_ISL_512998	SaudiArabia/KAUST-JEDDAH755/2020	2020/4/2	Pathogen Genomics Lab King Abdullah University of Science and Technology(KAUST)	Amit Kumar Subudhi et al
B.1.399	EPI_ISL_483449	USA/CA-ALSR-0993-SAN/2020	2020/4/26	Andersen lab at Scripps Research	SEARCH Alliance San Diego with David Pride et al
B.1.40	EPI_ISL_425906	Scotland/EDB124/2020	2020/3/18	COVID-19 Genomics UK (COG-UK) Consortium	McHugh M et al
B.1.400	EPI_ISL_483241	USA/CA-ALSR-0585-SAN/2020	2020/4/15	Andersen lab at Scripps Research	SEARCH Alliance San Diego with David Pride et al
B.1.400.1	EPI_ISL_2423567	USA/AZ-TG812013/2020	2020/11/9	TGen North	"Jolene Bowers et al
B.1.401	EPI_ISL_636164	USA/CA-ALSR-3720/2020	2020/6/4	Andersen lab at Scripps Research	SEARCH Alliance San Diego with Tracy Basler et al
B.1.403	EPI_ISL_1137163	USA/CA-CZB-16547/2020	2020/7/13	Chan-Zuckerberg Biohub	CZB Clinhub Consortium et al
B.1.404	EPI_ISL_1523526	USA/OR-OSPHL00196/2020	2020/3/16	Oregon State Public Health Laboratory	LaDonna Grenz et al
B.1.405	EPI_ISL_483281	USA/CA-ALSR-0653-IPL/2020	2020/4/30	Andersen lab at Scripps Research	SEARCH Alliance San Diego with David Pride et al
B.1.406	EPI_ISL_618273	Denmark/DCGC-1957/2020	2020/7/6	Albertsen lab, Department of Chemistry and Bioscience, Aalborg University, Denmark	Danish Covid-19 Genome Consortia et al

B.1.407	EPI_ISL_554831	England/MILK-9586FF/2020	2020/6/9	Wellcome Sanger Institute for the COVID-19 Genomics UK (COG-UK) consortium	The Lighthouse Lab in Milton Keynes et al
B.1.408	EPI_ISL_455469	Romania/283584/2020	2020/5/12	Cantacuzino Institute	M.Lazar et al
B.1.409	EPI_ISL_5384435	USA/TX-HMH-MCoV-49655/2020	2020/7/13	Houston Methodist Hospital	Randall J. Olsen et al
B.1.411	EPI_ISL_602574	SriLanka/GQC12/2020	2020/10/23	Centre for Dengue Research, Department of Immunology and Molecular Medicine	Chandima Jeewandara et al
B.1.413	EPI_ISL_604591	USA/UT-QDX-2230/2020	2020/3/12	Quest Diagnostics	Rosenthal et al
B.1.415	EPI_ISL_801588	Chile/Santiago-op7d1/2020	2020/3/16	MSHS Pathogen Surveillance Program	Leonardo I. Almonacid et al
B.1.416	EPI_ISL_480788	Senegal/1938/2020	2020/3/31	Institut Pasteur de Dakar	Ndongo Dia et al
B.1.416.1	EPI_ISL_560598	France/BRE-8930/2020	2020/7/24	National Reference Center for Viruses of Respiratory Infections, Institut Pasteur, Paris	Sylvie Behillil et al
B.1.417	EPI_ISL_509289	SouthAfrica/KRISP-K000640/2020	2020/7/13	KRISP, KZN Research Innovation and Sequencing Platform	Giandhari J et al
B.1.418	EPI_ISL_439237	Scotland/EDB2599/2020	2020/4/24	COVID-19 Genomics UK (COG-UK) Consortium	McHugh M et al
B.1.420	EPI_ISL_498154	Colombia/CUN-INS-88496/2020	2020/3/25	Instituto Nacional de Salud, Bogotá, Colombia	Katherine Laiton-Donato et al
B.1.421	EPI_ISL_2628353	USA/CA-SEARCH-101735/2020	2020/7/28	Andersen lab at Scripps Research	SEARCH Alliance San Diego with Ashleigh Murphy et al
B.1.422	EPI_ISL_464051	Canada/ON-UHTC_0004/2020	2020/3/18	Ontario Institute for Cancer Research	Ramzi Fattouh et al
B.1.423	EPI_ISL_579013	USA/LA-EVTL609/2020	2020/7/1	Microbial Genome Sequencing Center	Jeremy P. Kamil et al
B.1.424	EPI_ISL_604071	USA/MN-QDX-1722/2020	2020/3/16	Quest Diagnostics	Rosenthal et al
B.1.425	EPI_ISL_524337	USA/UT-08586/2020	2020/5/7	Utah Public Health Laboratory	Erin L. Young et al
B.1.426	EPI_ISL_449837	USA/UT-01607/2020	2020/4/6	Utah Public Health Laboratory	Erin Young et al
B.1.427	EPI_ISL_2304313	USA/CA-ACPHD-00310/2020	2020/4/11	Alameda County Public Health Department	Kristna Hsieh et al
B.1.428	EPI_ISL_415648	Denmark/SSI-104/2020	2020/3/3	ViFU	Morten Rasmussen et al
B.1.428.1	EPI_ISL_812258	Iraq/USAFSAM-S061/2020	2020/6/6	United States Air Force School of Aerospace Medicine	Anthony Fries et al

B.1.428.2	EPI_ISL_803817	Tunisia/12-8274/2020	2020/4/7	Clinical and Experimental Pharmacology Lab, LR16SP02, National Center of Pharmacovigilance, University of Tunis El Manar, Tunis, Tunisia. 2- Neurodegenerative diseases and psychiatric troubles, LR18SP03, Razi Hospital, University of Tunis El Manar, Tunis, Tunisia. 3- Ministry of Health, National Observatory of New and Emerging Diseases, 1006, Tunis, Tunisia	Ilhem Boutiba-Ben Boubaker et al
B.1.428.3	EPI_ISL_1714216	Qatar/QA-WCMQ_FD17462393/2020	2020/6/4	Weill Cornell Medical College - Qatar (WCM-Q), Genomics Core Laboratory / Qatar Genome Project (QGP)	WCMQ: Ayeda A. Ahmed et al
B.1.429	EPI_ISL_1675148	USA/NV-NSPHL-357909/2020	2020/4/15	Nevada State Public Health Laboratory	Andrew Gorzalski et al
B.1.429.1	EPI_ISL_1708775	USA/NY-MSHSPSP-PV24036/2020	2020/12/2	MSHS Pathogen Surveillance Program	Ana S. Gonzalez-Reiche et al
B.1.431	EPI_ISL_489039	Scotland/EDB2810/2020	2020/3/30	Wellcome Sanger Institute for the COVID-19 Genomics UK (COG-UK) consortium	McHugh M et al
B.1.432	EPI_ISL_5254069	USA/CO-CDPHE-2004270037/2020	2020/4/25	Colorado Department of Public Health and Environment	Laura Bankers et al
B.1.433	EPI_ISL_2223502	USA/TX-HMH-MCoV-40320/2020	2020/8/3	Houston Methodist Hospital	Randall J. Olsen et al
B.1.434	EPI_ISL_426677	Australia/VIC369/2020	2020/3/22	Microbiological Diagnostic Unit Public Health Laboratory and Victorian Infectious Diseases Reference Laboratory, Doherty Institute	Caly L. et al
B.1.435	EPI_ISL_695939	USA/AZ-TG648768/2020	2020/4/10	TGen North	Jolene Bowers et al
B.1.436	EPI_ISL_524895	USA/MD-MDH-0130/2020	2020/7/8	MD PHL	Maryland Department of Health Laboratories Administration et al
B.1.437	EPI_ISL_653200	USA/FL-BPHL-2047/2020	2020/6/23	Florida Bureau of Public Health Laboratories	Sarah Schmedes et al
B.1.438	EPI_ISL_478710	Australia/NSW598/2020	2020/6/8	NSW Health Pathology - Institute of Clinical Pathology and Medical Research; Westmead Hospital; University of Sydney	CIDM-PH et al.

B.1.438.2	EPI_ISL_855367	Mayotte/IPP00174/2021	2021/1/6	National Reference Center for Viruses of Respiratory Infections, Institut Pasteur, Paris	Marion Barbet et al
B.1.438.3	EPI_ISL_1303308	USA/SC-DHEC-0307/2021	2021/2/1	Microbiology Division, South Carolina Department of Health and Environmental Control	Flores et al
B.1.438.4	EPI_ISL_5696417	USA/CA-CDPH-MC1205035/2020	2020/10/19	California Department of Public Health	Emily Smith on behalf of CDPH-COVIDNet et al
B.1.439	EPI_ISL_604495	USA/UT-QDX-2129/2020	2020/3/13	Quest Diagnostics	Rosenthal et al
B.1.44	EPI_ISL_421005	Wales/PHWC-25560/2020	2020/3/23	Public Health Wales Microbiology Cardiff	Catherine Moore et al
B.1.441	EPI_ISL_3726044	USA/TN-VUMC-000070/2020	2020/3/13	Dr. Suman Das Lab - Vanderbilt University Medical Center (VUMC) (https://my.vanderbilt.edu/daslab/)	Grant Vestal et al
B.1.442	EPI_ISL_485841	USA/VA-DCLS-0540/2020	2020/3/12	Virginia DCLS	Virginia DCLS et al
B.1.443	EPI_ISL_1168366	USA/NH-Yale-1640/2020	2020/4/18	Grubaugh Lab - Yale School of Public Health	Mary Petrone et al
B.1.444	EPI_ISL_525343	USA/UT-UPHL-04478/2020	2020/5/21	Utah Public Health Laboratory	Erin Young et al
B.1.445	EPI_ISL_696360	USA/AZ-TG670160/2020	2020/4/5	TGen North	Jolene Bowers et al
B.1.446	EPI_ISL_424174	USA/CT-UW-1365/2020	2020/3/13	UW Virology Lab	Pavitra Roychoudhury et al
B.1.448	EPI_ISL_826613	USA/NY-AECOM_031/2020	2020/4/15	Albert Einstein College of Medicine, Dept. of Microbiology & Immunology, Chandran lab	J. Maximilian Fels et al
B.1.450	EPI_ISL_434718	USA/TX-HMH07/2020	2020/3/15	Houston Methodist Hospital	S. Wesley Long et al
B.1.451	EPI_ISL_500846	USA/VA-DCLS-0684/2020	2020/4/18	Virginia DCLS	Virginia DCLS et al
B.1.452	EPI_ISL_513791	USA/CA-CZB-2341/2020	2020/5/12	Chan-Zuckerberg Biohub	CZB Cliahub Consortium et al
B.1.452	EPI_ISL_2224736	USA/TX-HMH-MCoV-41000/2020	2020/7/21	Houston Methodist Hospital	Randall J. Olsen et al
B.1.453	EPI_ISL_571702	USA/CA-QDX-1037/2020	2020/3/17	Quest Diagnostics	Rosenthal et al
B.1.456	EPI_ISL_490007	SaudiArabia/610/2020	2020/2/20	King Fahad Medical City	Alosaimi et al
B.1.458	EPI_ISL_471590	India/TG-CCMB-L1451/2020	2020/5/25	CSIR-Centre for Cellular and Molecular Biology	Lamuk Zaveri et al
B.1.459	EPI_ISL_428715	Turkey/HSGM-5624/2020	2020/3/18	Ministry of Health Turkey	Fatma Bayrakdar et al
B.1.460	EPI_ISL_567528	England/QEUH-9C9F1C/2020	2020/9/10	Wellcome Sanger Institute for the COVID-19 Genomics UK (COG-UK) consortium	Harper VanSteenhouse et al

B.1.462	EPI_ISL_729920	Nigeria/KD-CV233/2020	2020/4/20	African Centre of Excellence for Genomics of Infectious Diseases (ACEGID), Redeemer's University, Ede, Osun State, Nigeria	Oluniyi P.E. et al
B.1.463	EPI_ISL_737061	Finland/S12A1/2020	2020/9/10	Department of Virology, Faculty of Medicine, University of Helsinki, Helsinki, Finland	Teemu Smura et al
B.1.465	EPI_ISL_1184553	Finland/10MS2E4/2020	2020/3/10	Department of Virology, Faculty of Medicine, University of Helsinki, Helsinki, Finland	Teemu Smura et al
B.1.466	EPI_ISL_482765	Egypt/MASRI-014/2020	2020/4/30	Medical Ain Shams Research Institute (MASRI), Ain Shams University	Hesham Elghazaly et al
B.1.466.1	EPI_ISL_1015121	France/IDF_PSL_384/2020	2020/6/9	Department of Virology, Pitié-Salpêtrière hospital	Valentin Leducq et al
B.1.466.2	EPI_ISL_2868921	Indonesia/BT-NIHRD-WGS03289/2020	2020/8/6	National Institute of Health Research and Development	Vivi Setiawaty et al
B.1.467	EPI_ISL_730012	Nigeria/BO-CV347/2020	2020/5/17	African Centre of Excellence for Genomics of Infectious Diseases (ACEGID), Redeemer's University, Ede, Osun State, Nigeria	Oluniyi P.E. et al
B.1.468	EPI_ISL_490001	SaudiArabia/545/2020	2020/2/13	King Fahad Medical City	Alosaimi et al
B.1.469	EPI_ISL_552561	England/MILK-97551D/2020	2020/8/21	Wellcome Sanger Institute for the COVID-19 Genomics UK (COG-UK) consortium	The Lighthouse Lab in Milton Keynes et al
B.1.470	EPI_ISL_759965	Indonesia/JI-ITDua-2858NTv/2020	2020/4/9	Institute of Tropical Disease, Universitas Airlangga	Rima R Prasetya et al
B.1.471	EPI_ISL_437496	SaudiArabia/KAUST-Jeddah59/2020	2020/3/25	Pathogen Genomics Lab King Abdullah University of Science and Technology(KAUST)	Sara Mfarrej et al
B.1.473	EPI_ISL_426428	USA/NC-CDC-03068803-001/2020	2020/3/8	Pathogen Discovery, Respiratory Viruses Branch, Division of Viral Diseases, Centers for Disease Control and Prevention	Krista Queen et al
B.1.474	EPI_ISL_1015160	France/IDF_PSL_415/2020	2020/9/21	Department of Virology, Pitié-Salpêtrière hospital	Valentin Leducq et al
B.1.475	EPI_ISL_753805	Germany/BE-ChVir-W1302-8173/2020	2020/4/29	Charité Universitätsmedizin Berlin, Institut für Virologie	Victor M Corman et al

B.1.476	EPI_ISL_2596774	France/PAC-IHU-10734_Illu1/2020	2020/4/2	MEPHI, Aix Marseille University	Anthony LEVASSEUR et al
B.1.478	EPI_ISL_2157568	Haiti/34003/2020	2020/5/15	Laboratory of Respiratory Viruses and Measles, Oswaldo Cruz Institute, FIOCRUZ	Paola Resende et al
B.1.479	EPI_ISL_460364	USA/MA-MGH-00136/2020	2020/3/25	Infectious Disease Program, Broad Institute of Harvard and MIT	Lemieux et al
B.1.480	EPI_ISL_512722	Australia/WA69/2020	2020/7/3	PathWest Laboratory Medicine WA Microbial Surveillance Unit	PathWest Laboratory Medicine WA Microbial Surveillance Unit et al
B.1.482	EPI_ISL_420045	France/GES-2843/2020	2020/3/17	National Reference Center for Viruses of Respiratory Infections, Institut Pasteur, Paris	Mélanie Albert et al
B.1.483	EPI_ISL_428254	USA/WI-UW-158/2020	2020/3/15	University of Wisconsin-Madison AIDS Vaccine Research Laboratories	Gage Moreno et al
B.1.485	EPI_ISL_2246395	USA/MI-MDHHS-SC26958/2020	2020/4/27	Michigan Department of Health and Human Services, Bureau of Laboratories	Blankenship HM et al
B.1.486	EPI_ISL_593478	USA/MA-JLL-D18/2020	2020/4/28	Jonathan Li Laboratory	Manish C. Choudhary et al
B.1.487	EPI_ISL_545210	USA/TX-HMH-2260/2020	2020/5/24	Houston Methodist Hospital	S. Wesley Long et al
B.1.488	EPI_ISL_534228	Sweden/20-08938/2020	2020/7/13	The Public Health Agency of Sweden	Anna-Malin Linde et al
B.1.489	EPI_ISL_422164	Wales/PHWC-26620/2020	2020/3/26	Public Health Wales Microbiology Cardiff	Catherine Moore et al
B.1.490	EPI_ISL_481595	Finland/20A16S7/2020	2020/4/20	Department of Virology, Faculty of Medicine, University of Helsinki, Helsinki, Finland	Teemu Smura et al
B.1.491	EPI_ISL_438718	England/CAMB-83FBD/2020	2020/4/22	COVID-19 Genomics UK (COG-UK) Consortium	Luke W Meredith et al
B.1.492	EPI_ISL_677289	USA/CO-CDPHE-2004230036/2020	2020/4/21	Colorado Department of Puplic Health and Environment	Laura Bankers et al
B.1.493	EPI_ISL_508860	USA/FL-BPHL-0656/2020	2020/4/7	Florida Bureau of Public Health Laboratories	Sarah Schmedes et al
B.1.494	EPI_ISL_2691720	USA/MD-HP13053-PIDCLFMKUI/2020	2020/3/31	Johns Hopkins Hospital Department of Pathology	C. Paul Morris et al
B.1.495	EPI_ISL_482357	USA/OR-PROV-086/2020	2020/4/19	Providence St. Joseph Health Molecular Genomics Laboratory	Alexa K Dowdell et al
B.1.496	EPI_ISL_444550	USA/IL-NM054/2020	2020/3/15	Ozer Lab	Ramon Lorenzo-Redondo et al
B.1.498	EPI_ISL_494523	USA/NC-QDX-171/2020	2020/4/27	Quest Diagnostics	Anderson et al

B.1.499	EPI_ISL_4405694	Argentina/PAIS-A1026/2020	2020/1/1	Área de Secuenciación del Laboratorio de Virología del Hospital de Niños Dr. Ricardo Gutierrez on behalf of 'Proyecto Argentino Interinstitucional de genomica de SARS-CoV-2' (PAIS Consortium)	Nabaes Jodar et al
B.1.499	EPI_ISL_1396437	Argentina/PAIS-H0010/2020	2020/8/25	Laboratorio Mixto de Biotecnología Acuática (LMBA) on behalf of 'Proyecto Argentino Interinstitucional de genomica de SARS-CoV-2' (PAIS Consortium)	Joaquín Ezpeleta et al
B.1.501	EPI_ISL_494526	USA/NC-QDX-174/2020	2020/4/27	Quest Diagnostics	Anderson et al
B.1.502	EPI_ISL_544032	USA/TX-HMH-3156/2020	2020/6/11	Houston Methodist Hospital	S. Wesley Long et al
B.1.503	EPI_ISL_526828	USA/VA-DCLS-0612/2020	2020/4/1	Virginia DCLS	Virginia DCLS et al
B.1.504	EPI_ISL_474971	Israel/CVL-n-9560/2020	2020/4/19	Israel Central Virology laboratory	Neta Zuckerman et al
B.1.505	EPI_ISL_447381	Israel/2086004/2020	2020/4/1	Stern Lab	Stern Lab et al
B.1.506	EPI_ISL_525686	USA/NY-Wadsworth-57118-01/2020	2020/4/20	Wadsworth Center, New York State Department of Health	Kirsten St. George et al
B.1.507	EPI_ISL_450050	USA/NY-PV09362/2020	2020/3/15	MSHS Pathogen Surveillance Program	Ana S. Gonzalez-Reiche et al
B.1.508	EPI_ISL_754930	USA/CA-CDPH041/2020	2020/4/28	California Department of Public Health	CDPH IDLB COVIDNet et al
B.1.509	EPI_ISL_653139	USA/FL-BPHL-1986/2020	2020/4/18	Florida Bureau of Public Health Laboratories	Sarah Schmedes et al
B.1.510	EPI_ISL_2426012	Norway/1766-2/2020	2020/3/6	Norwegian Institute of Public Health, Department of Virology	Kathrine Stene-Johansen et al
B.1.511	EPI_ISL_542678	USA/TX-HMH-1371/2020	2020/5/2	Houston Methodist Hospital	S. Wesley Long et al
B.1.513	EPI_ISL_451999	Denmark/ALAB-HH-115/2020	2020/3/9	Albertsen lab, Department of Chemistry and Bioscience, Aalborg University, Denmark	Rasmus Kirkegaard et al
B.1.515	EPI_ISL_462706	USA/MI-MDHHS-SC20624/2020	2020/4/17	Michigan Department of Health and Human Services, Bureau of Laboratories	Blankenship HM et al
B.1.516	EPI_ISL_631862	USA/NY-NYCPHL-000356/2020	2020/3/11	New York City Public Health Laboratory	Jade Wang et al
B.1.517	EPI_ISL_2976222	USA/ID-IBL-614702/2020	2020/4/6	Idaho Bureau of Laboratories	"R. Beukelman et al
B.1.517.1	EPI_ISL_710125	Australia/NSW1227/2020	2020/12/2	CIDM-PH et al.	CIDM-PH et al.
B.1.518	EPI_ISL_426054	USA/CT-UW-4347/2020	2020/3/30	UW Virology Lab	Pavitra Roychoudhury et al
B.1.520	EPI_ISL_582289	Canada/MB-NML-617/2020	2020/3/25	National Microbiology Laboratory (NML)	Anna Majer et al
B.1.521	EPI_ISL_508793	USA/FL-BPHL-0589/2020	2020/3/21	Florida Bureau of Public Health Laboratories	Sarah Schmedes et al

B.1.523	EPI_ISL_437733	SaudiArabia/KAUST-Madinah238/2020	2020/3/29	Pathogen Genomics Lab King Abdullah University of Science and Technology(KAUST)	Sharif Hala et al
B.1.524	EPI_ISL_644671	Malaysia/IMR_CV140342/2020	2020/9/1	Institute for Medical Research, Infectious Disease Research Centre, National Institutes of Health, Ministry of Health Malaysia	Suppiah J et al
B.1.525	EPI_ISL_4739567	Sudan/N6667/2020	2020/10/4	National Institute for Communicable Diseases of the National Health Laboratory Service	Elham Rizgalla et al
B.1.526	EPI_ISL_3364539	USA/NY-GEO-0231/2020	2020/1/28	Biotia	Dorottya Nagy-Szakal et al
B.1.527	EPI_ISL_636467	Italy/VEN-IZSVe-14-21/2020	2020/3/24	Istituto Zooprofilattico Sperimentale delle Venezie	Adelaide Milani et al
B.1.528	EPI_ISL_451400	Morocco/OUA677-19/2020	2020/4/23	Laboratoire de Recherche et d'Analyse Médicale de la Gendarmerie Royale	Sanaâ LEMRISS et al
B.1.529	EPI_ISL_645201	France/OCC-SC545/2020	2020/4/2	CNR Virus des Infections Respiratoires - France SUD	Antonin Bal et al
B.1.529	EPI_ISL_1133148	Italy/LOM-Pavia-48855/2020	2020/4/28	Molecular Virology Unit, Microbiology and Virology Depertment, Fondazione IRCCS Policlinico San Matteo, Pavia	Monica Tallarita et al
B.1.530	EPI_ISL_3049453	Kenya/C57901/2020	2020/9/15	KEMRI-Wellcome Trust Research Programme,Kilifi	Githinji G. et al
B.1.531	EPI_ISL_783074	England/MILK-BAB136/2020	2020/11/18	Wellcome Sanger Institute for the COVID-19 Genomics UK (COG-UK) Consortium	The Lighthouse Lab in Milton Keynes et al
B.1.532	EPI_ISL_556109	England/MILK-6422BB/2020	2020/7/4	Wellcome Sanger Institute for the COVID-19 Genomics UK (COG-UK) consortium	The Lighthouse Lab in Milton Keynes et al
B.1.533	EPI_ISL_512937	SaudiArabia/KAUST-JEDDAH494/2020	2020/4/9	Pathogen Genomics Lab King Abdullah University of Science and Technology(KAUST)	Fadwa Alofi et al
B.1.534	EPI_ISL_1876157	Denmark/DCGC-67586/2020	2020/11/2	Aalborg University	Danish Covid-19 Genome Consortium et al
B.1.535	EPI_ISL_776198	Germany/HH-hpi-p1847/2020	2020/3/22	Heinrich Pette Institute, Leibniz Institute for Experimental Virology	Alexis Robitaille et al
B.1.536	EPI_ISL_616955	Denmark/DCGC-8136/2020	2020/10/26	Albertsen lab, Department of Chemistry and Bioscience, Aalborg University, Denmark	Danish Covid-19 Genome Consortia et al

B.1.537	EPI_ISL_528810	India/TG-CCMB-GC1/2020	2020/5/24	CSIR-Centre for Cellular and Molecular Biology	Thrilok Chander Bingi et al
B.1.538	EPI_ISL_476890	India/MP-DRDE-4022/2020	2020/5/16	Defence Research & Development Establishment (DRDE)	Shashi Sharma et al
B.1.539	EPI_ISL_1624329	USA/VA_NIDDL_27852/2020	2020/10/25	Naval Medical Research Center Biological Defense Research Directorate	Logan Voegtly et al
B.1.540	EPI_ISL_455640	India/WB-S4/2020	2020/3/27	National Institute of Biomedical Genomics	Arindam Maitra et al
B.1.541	EPI_ISL_579016	USA/LA-EVTL612/2020	2020/7/1	Microbial Genome Sequencing Center	Jeremy P. Kamil et al
B.1.543	EPI_ISL_801878	USA/NY-MSHSPSP-PV17906/2020	2020/8/30	MSHS Pathogen Surveillance Program	Ana S. Gonzalez-Reiche et al
B.1.544	EPI_ISL_581493	Congo/UKT-015/2020	2020/6/25	NGS Competence Center Tübingen, Institut für Medizinische Mikrobiologie und Hygiene, Universitätsklinikum Tübingen	Angel Angelov et al
B.1.545	EPI_ISL_579469	NewZealand/20VR3181/2020	2020/3/25	Institute of Environmental Science and Research (ESR)	Xiaoyun Ren et al
B.1.546	EPI_ISL_545546	USA/TX-HMH-2776/2020	2020/6/3	Houston Methodist Hospital	S. Wesley Long et al
B.1.547	EPI_ISL_535765	Canada/QC-LSPQ-L00217574/2020	2020/3/13	Laboratoire de santé publique du Québec	Sandrine Moreira et al
B.1.548	EPI_ISL_471623	India/TG-CCMB-L1693/2020	2020/5/29	CSIR-Centre for Cellular and Molecular Biology	Payel Mukherjee et al
B.1.549	EPI_ISL_2779501	Kenya/MAL-5042/2020	2020/6/9	USAMRD-A, Basic Science Laboratory	Gathii Kimita et al
B.1.550	EPI_ISL_872402	USA/AL-Morgan-JDTB/2020	2020/10/3	Hudsonalpha Genome Sequencing Center	Jane Grimwood et al
B.1.551	EPI_ISL_837802	Mexico/CMX-INER-0208/2020	2020/8/20	Instituto Nacional de Enfermedades Respiratorias (INER)	Celia Boukadida et al
B.1.552	EPI_ISL_467971	USA/CA-SR0447/2020	2020/3/30	Andersen lab at Scripps Research	SEARCH Alliance San Diego with Tracy Basler et al
B.1.554	EPI_ISL_2678230	USA/UT-UPHL-2105275840/2020	2020/4/21	Utah Public Health Laboratory	Erin L. Young et al
B.1.555	EPI_ISL_445221	Sweden/20-05444/2020	2020/3/11	The Public Health Agency of Sweden	Frida Ahlfors et al
B.1.556	EPI_ISL_648834	USA/CA-ALSR-4125/2020	2020/6/10	Andersen lab at Scripps Research	SEARCH Alliance San Diego with Tracy Basler et al
B.1.557	EPI_ISL_459336	NorthernIreland/NIRE-FA7AA/2020	2020/4/6	Wellcome Sanger Institute for the COVID-19 Genomics UK (COG-UK) consortium	Conall McCaughey et al

B.1.558	EPI_ISL_437575	USA/CA-SR0225/2020	2020/4/6	Andersen lab at Scripps Research	SEARCH Alliance San Diego with Michael Quigley et al
B.1.559	EPI_ISL_439142	Scotland/CVR989/2020	2020/3/29	COVID-19 Genomics UK (COG-UK) Consortium	Ana da Silva Filipe et al
B.1.560	EPI_ISL_1383217	Spain/NC-CHN-01000049/2020	2020/3/20	Centro de Secuenciación NASERTIC	Carmen Ezpeleta Baquedano et al
B.1.561	EPI_ISL_1531819	Mexico/BCN-ALSR-8354/2020	2020/6/18	Andersen lab at Scripps Research	SEARCH Alliance San Diego with Idanya Rubi Serafin Higuera et al
B.1.562	EPI_ISL_761042	England/ALDP-CB2E16/2020	2020/12/1	Wellcome Sanger Institute for the COVID-19 Genomics UK (COG-UK) Consortium	Jacquelyn Wynn et al
B.1.563	EPI_ISL_709546	England/ALDP-C06DCB/2020	2020/12/6	Wellcome Sanger Institute for the COVID-19 Genomics UK (COG-UK) Consortium	Jacquelyn Wynn et al
B.1.564	EPI_ISL_1165135	USA/OH-ODH-SC149737/2020	2020/3/11	Ohio Department of Health Laboratory	Holmes et al
B.1.564.1	EPI_ISL_591187	Canada/ON-S1416/2020	2020/8/23	McMaster University	Allison McGeer et al
B.1.565	EPI_ISL_746393	USA/UT-UPHL-2012514374/2020	2020/6/18	Utah Public Health Laboratory	Erin Young et al
B.1.566	EPI_ISL_571800	USA/TX-QDX-984/2020	2020/3/16	Quest Diagnostics	Rosenthal et al
B.1.567	EPI_ISL_460055	USA/MN-MDH-152/2020	2020/3/23	Minnesota Department of Health, Public Health Laboratory	Matt Plumb et al
B.1.568	EPI_ISL_872412	USA/AL-Shelby-JDTM/2020	2020/10/5	Hudsonalpha Genome Sequencing Center	Jane Grimwood et al
B.1.570	EPI_ISL_5253966	USA/CO-CDPHE-2007100817/2020	2020/7/6	Colorado Department of Public Health and Environment	Laura Bankers et al
B.1.571	EPI_ISL_543079	USA/TX-HMH-3627/2020	2020/6/15	Houston Methodist Hospital	S. Wesley Long et al
B.1.572	EPI_ISL_434942	USA/TX-HMH0348/2020	2020/4/2	Houston Methodist Hospital	S. Wesley Long et al
B.1.573	EPI_ISL_968263	Canada/BC-BCCDC-3565/2020	2020/6/24	BCCDC Public Health Laboratory	Prystajecky Natalie et al
B.1.574	EPI_ISL_1138489	USA/IL-IDPH-COO-C-000663/2020	2020/5/1	Gagnon Lab, Southern Illinois University	Keith Gagnon et al
B.1.575	EPI_ISL_786939	USA/TX-HMH-MCoV-15455/2020	2020/10/23	Houston Methodist Hospital	S. Wesley Long et al
B.1.575.1	EPI_ISL_4425102	Spain/MD-ISCIII-215564/2020	2020/11/30	Instituto de Salud Carlos III	Vázquez-Morón et al
B.1.575.2	EPI_ISL_2776189	DominicanRepublic/Yale-5507/2021	2021/1/14	Grubaugh Lab - Yale School of Public Health	Joseph Fauver et al

B.1.576	EPI_ISL_2877403	France/HDF-IPP3652/2020	2020/3/25	National Reference Center for Viruses of Respiratory Infections, Institut Pasteur, Paris	Marion Barbet et al
B.1.577	EPI_ISL_542702	USA/TX-HMH-1404/2020	2020/4/17	Houston Methodist Hospital	S. Wesley Long et al
B.1.578	EPI_ISL_604296	USA/CA-QDX-1984/2020	2020/3/17	Quest Diagnostics	Rosenthal et al
B.1.579	EPI_ISL_1113373	USA/AZ-TG724120/2020	2020/4/24	TGen North	"Jolene Bowers et al
B.1.581	EPI_ISL_543334	USA/TX-HMH-4060/2020	2020/6/18	Houston Methodist Hospital	S. Wesley Long et al
B.1.582	EPI_ISL_3275380	Maldives/MAV00499/2020	2020/4/11	Indira Gandhi Memorial Hospital	Dr. Milza Abdul Muhsin et al
B.1.585	EPI_ISL_786330	USA/TX-HMH-MCoV-14846/2020	2020/10/13	Houston Methodist Hospital	S. Wesley Long et al
B.1.586	EPI_ISL_468582	USA/NY-QDX-87/2020	2020/3/12	Quest Diagnostics	Anderson et al
B.1.587	EPI_ISL_667079	USA/OR-OHSU-0070/2020	2020/4/23	Oregon SARS-CoV-2 Genome Sequencing Center	Brendan L. O'Connell et al
B.1.588	EPI_ISL_1168693	PuertoRico/PR-CDC-S172/2020	2020/8/2	Centers for Disease Control and Prevention, Dengue Branch	Gilberto A. Santiago et al
B.1.588.1	EPI_ISL_738317	Romania/USAFSAM-S556/2020	2020/11/28	United States Air Force School of Aerospace Medicine	Anthony Fries et al
B.1.589	EPI_ISL_572109	USA/MN-QDX-1320/2020	2020/3/15	Quest Diagnostics	Rosenthal et al
B.1.590	EPI_ISL_572166	USA/MN-QDX-1377/2020	2020/3/15	Quest Diagnostics	Rosenthal et al
B.1.591	EPI_ISL_1494524	USA/IL-IDPH-WIN-S-0002001/2020	2020/8/27	Gagnon Lab, Southern Illinois University	Keith Gagnon et al
B.1.592	EPI_ISL_889857	Canada/QC-JUS-V6011098/2020	2020/3/30	Laboratoire de santé publique du Québec	Sandrine Moreira et al
B.1.593	EPI_ISL_5440637	USA/CO-CDPHE-2007070535/2020	2020/7/3	Colorado Department of Public Health and Environment	Laura Bankers et al
B.1.595	EPI_ISL_424868	USA/LA-CDC-03068669-001/2020	2020/3/9	Pathogen Discovery, Respiratory Viruses Branch, Division of Viral Diseases, Centers for Disease Control and Prevention	Yan Li et al
B.1.595.1	EPI_ISL_804868	USA/DC-DFS-PHL-0076/2020	2020/5/16	DC Public Health Lab/ Dept. of Forensic Sciences	Scott Nguyen et al
B.1.595.2	EPI_ISL_914336	USA/AZ-TG375489/2020	2020/6/14	TGen North	"Jolene Bowers et al
B.1.595.3	EPI_ISL_1482724	USA/SC-MUSC-0476/2020	2020/6/27	MUSC Molecular Pathology Laboratory	Julie W. Hirschhorn et al
B.1.595.4	EPI_ISL_477276	USA/MN-MDH-1186/2020	2020/4/20	Minnesota Department of Health, Public Health Laboratory	Matt Plumb et al
B.1.596	EPI_ISL_590772	USA/MN-MDH-1768/2020	2020/4/11	Minnesota Department of Health, Public Health Laboratory	Matt Plumb et al

B.1.596.1	EPI_ISL_3049403	Kenya/C54633/2020	2020/9/7	KEMRI-Wellcome Trust Research Programme,Kilifi	Githinji G. et al
B.1.597	EPI_ISL_699656	Tunisia/5008/2020	2020/3/24	1-Clinical and Experimental Pharmacology Lab, LR16SP02, National Center of Pharmacovigilance, University of Tunis El Manar, Tunis, Tunisia. 2- Neurodegenerative diseases and psychiatric troubles, LR18SP03, Razi Hospital, University of Tunis El Manar, Tunis, Tunisia. 3- Ministry of Health, National Observatory of New and Emerging Diseases, 1006, Tunis, Tunisia	Ilhem Boutiba-Ben Boubaker et al
B.1.598	EPI_ISL_444679	USA/NY-NYUMC562/2020	2020/3/30	Departments of Pathology and Medicine, New York University School of Medicine	Maria Aguero-Rosenfeld et al
B.1.599	EPI_ISL_582465	Canada/MB-NML-1134/2020	2020/7/20	National Microbiology Laboratory (NML)	Anna Majer et al
B.1.6	EPI_ISL_475866	Austria/CeMM0449/2020	2020/3/5	Bergthaler laboratory, CeMM Research Center for Molecular Medicine of the Austrian Academy of Sciences	Alexandra Popa et al
B.1.600	EPI_ISL_1167817	Chile/VS-39494/2020	2020/4/7	Instituto de Salud Publica de Chile	Javier Tognarelli et al
B.1.601	EPI_ISL_5736723	Italy/EMR-UA-02_00003/2020	2020/3/4	Laboratory of Medical Microbiology, University of Antwerp	Mathias Smet et al
B.1.601	EPI_ISL_2923967	USA/NY-PRL-2021_0706_51F19/2020	2020/10/28	Pandemic Response Lab, R&D	Henry Lee et al
B.1.602	EPI_ISL_525951	USA/OR-OHSU-1023/2020	2020/7/7	Oregon SARS-CoV-2 Genome Sequencing Center	Brendan L. O'Connell et al
B.1.603	EPI_ISL_683880	USA/NY-NYCPHL-001318/2020	2020/11/10	New York City Public Health Laboratory	Jade Wang et al
B.1.604	EPI_ISL_876038	USA/NY-AECOM_109/2020	2020/7/28	Albert Einstein College of Medicine, Dept. of Microbiology & Immunology, Chandran lab	J. Maximilian Fels et al
B.1.605	EPI_ISL_522781	USA/VA-DCLS-0908/2020	2020/5/12	Virginia DCLS	Virginia DCLS et al
B.1.606	EPI_ISL_545165	USA/TX-HMH-2178/2020	2020/5/21	Houston Methodist Hospital	S. Wesley Long et al
B.1.607	EPI_ISL_420221	England/SHEF-C03E5/2020	2020/3/29	Department of Infection, Immunity and Cardiovascular Disease, The Florey Institute, The Medical School, University of Sheffield	Thushan de Silva et al

B.1.609	EPI_ISL_419680	Spain/VC-FISABIO-16/2020	2020/3/10	Sequencing and Bioinformatics Service and Molecular Epidemiology Research Group. FISABIO-Public Health	Maria Alma Bracho et al
B.1.609	EPI_ISL_2835331	Mexico/BCN-SEARCH-102851/2020	2020/4/28	Andersen lab at Scripps Research	SEARCH Alliance with Gisela Barrera Badillo et al
B.1.610	EPI_ISL_529513	England/ALDP-98EAC7/2020	2020/7/11	COVID-19 Genomics UK (COG-UK) Consortium	Sam Haldenby et al
B.1.611	EPI_ISL_825829	Canada/Qc-CHUM-2016103625A/2020	2020/6/9	Laboratoire de santé publique du Québec	Sandrine Moreira et al
B.1.612	EPI_ISL_590748	USA/MI-UM-MH27455/2020	2020/3/11	Lauring Lab, University of Michigan, Department of Microbiology and Immunology	Valesano et al
B.1.613	EPI_ISL_618805	Denmark/DCGC-1386/2020	2020/4/6	Albertsen lab, Department of Chemistry and Bioscience, Aalborg University, Denmark	Danish Covid-19 Genome Consortia et al
B.1.614	EPI_ISL_510871	Sweden/20-52071/2020	2020/6/15	The Public Health Agency of Sweden	Oskar Karlsson Lindsjo et al
B.1.615	EPI_ISL_825696	Canada/Qc-JUS-V927155399/2020	2020/7/26	Laboratoire de santé publique du Québec	Sandrine Moreira et al
B.1.616	EPI_ISL_1118892	France/BRE-IPP03808/2021	2021/2/10	National Reference Center for Viruses of Respiratory Infections, Institut Pasteur, Paris	Marion Barbet et al
B.1.617.1	EPI_ISL_2758215	India/un-IRSHA-CD210871/2020	2020/3/3	Communicable Diseases, Interactive Research School for Health Affairs (IRSHA)	Shrivastava et al
B.1.617.2	EPI_ISL_1914592	India/CT-AIIMS-Raipur-L-1010/2021	2021/3/30	State Virus Research and Diagnostic Laboratory (VRDL), AIIMS Raipur	Pushpendra Singh et al
B.1.617.2	EPI_ISL_2879009	India/MH-IGIB-GSEQ_1954/2021	2021/3/23	INSACOG at CSIR Institute of Genomics and Integrative Biology	INSACOG et al
B.1.617.2	EPI_ISL_5644723	India/GJ-INSACOG-GBRC2563/2021	2021/1/10	Gujarat Biotechnology Research Centre	Sonal Sharma et al
B.1.617.2	EPI_ISL_4768101	Rwanda/CV2197/2020	2020/9/10	Africa Centre for Excellence for Genomics of Infectious Diseases (ACEGID), Redeemer’s University	Enatha Mukantwari et al
B.1.617.2	EPI_ISL_2341920	India/CT-ILSGS01119/2021	2021/3/21	Institute of Life Sciences - INSACOG	Sunil K. Raghav et al
B.1.617.2	EPI_ISL_3614352	India/MN-1425500076304/2021	2021/4/25	National Institute of Biomedical Genomics – INSACOG	Arindam Maitra et al
B.1.617.2	EPI_ISL_4415533	India/MH-IISER-Pune-IP-00986/2021	2021/2/9	IISER Pune-INSACOG	Krishanpal Karmodiya et al

B.1.617.2	EPI_ISL_6025382	India/DL-ILBS-B_79/2021	2021/1/28	ILBS	Ekta Gupta et al
B.1.617.2	EPI_ISL_4415621	India/MH-IISER-Pune-IP-01021/2021	2021/2/9	IISER Pune-INSACOG	Krishanpal Karmodiya et al
B.1.617.2	EPI_ISL_3614353	India/MN-1425500076321/2021	2021/4/25	National Institute of Biomedical Genomics – INSACOG	Arindam Maitra et al
B.1.617.2	EPI_ISL_3215467	USA/CA-SEARCH-18281/2021	2021/4/11	Andersen lab at Scripps Research	Chip Schooley et al
B.1.617.2	EPI_ISL_4415418	India/MH-IISER-Pune-IP-00995/2021	2021/2/9	IISER Pune-INSACOG	Krishanpal Karmodiya et al
B.1.617.2	EPI_ISL_2842285	India/KA-NIMH-SEQ-1757/2021	2021/4/10	INSACOG-KA, NIMHANS	Chitra Pattabiraman et al
B.1.617.3	EPI_ISL_1415164	India/MH-NCCS-86945/2021	2021/2/13	National Centre For Cell Science – INSACOG	Dhiraj Paul et al
B.1.618	EPI_ISL_1419395	India/WB-1931300237615/2020	2020/10/27	National Institute of Biomedical Genomics – INSACOG	Arindam Maitra et al
B.1.619	EPI_ISL_3458197	Canada/QC-L00333705001A/2020	2020/3/10	Laboratoire de santé publique du Québec	Sandrine Moreira et al
B.1.619.1	EPI_ISL_2361093	SouthKorea/KDCA4323/2021	2021/2/22	Division of Emerging Infectious Diseases, Bureau of Infectious Diseases Diagnosis Control, Korea Disease Control and Prevention Agency	Ae Kyung Park et al
B.1.621	EPI_ISL_6512535	Colombia/ANT-LDSP461/2020	2020/10/14	Laboratorio Departamental de Salud Publica de Antioquia	Idabely Betancur Ortiz et al
B.1.621.1	EPI_ISL_4361670	USA/OH-CDC-ASC210055228/2021	2021/4/9	Centers for Disease Control and Prevention Division of Viral Diseases, Pathogen Discovery	Dakota Howard et al
B.1.622	EPI_ISL_2245960	Guinea/Conakry_IPG11718/2020	2020/9/16	Institut Pateur de Dakar	Grayo Solene et al
B.1.625	EPI_ISL_2339873	Colombia/COR-U27/2020	2020/11/16	Centro de Investigaciones en Microbiología y Biotecnología-UR (CIMBIUR), Facultad de Ciencias Naturales, Universidad del Rosario, Bogotá, Colombia	Salim Mattar et al
B.1.626	EPI_ISL_2125133	Germany/BY-RKI-I-141456/2021	2021/4/16	Robert Koch Institute	?
B.1.627	EPI_ISL_3568367	Australia/NSW-SAVID-3904/2021	2021/1/9	Virology Research Laboratory; Area of Virology, Serology and Virology Division (SAViD), New South Wales Health Pathology Randwick	Foster et al
B.1.630	EPI_ISL_2274018	DominicanRepublic/21915/2021	2021/3/17	Laboratory of Respiratory Viruses and Measles, Oswaldo Cruz Institute, FIOCRUZ	Paola Resende et al

B.1.631	EPI_ISL_2628735	USA/SEARCH-102258/2020	2020/12/16	Andersen lab at Scripps Research	SEARCH Alliance San Diego with Ashleigh Murphy et al
B.1.632	EPI_ISL_2343008	Mexico/TAM-InDRE_FB14701_S2256/2021	2021/4/28	Instituto de Diagnostico y Referencia Epidemiologicos (INDRE)	Claudia Wong-Arambula et al
B.1.633	EPI_ISL_2461371	India/DL-NCDC-2509105/2021	2021/4/14	NCDC Delhi, Biotechnology Division INSACOG	Manoj K Singh et al
B.1.634	EPI_ISL_1184562	USA/AZ-ASPHL-3709/2021	2021/3/2	Arizona State Public Health Laboratory	Trung Huynh et al
B.1.635	EPI_ISL_1168492	Mexico/VER-InDRE_414/2021	2021/1/16	Instituto de Diagnostico y Referencia Epidemiologicos (INDRE)	Claudia Wong-Arambula et al
B.1.637	EPI_ISL_801973	USA/NY-MSHSPSP-PV21166/2020	2020/10/9	MSHS Pathogen Surveillance Program	Ana S. Gonzalez-Reiche et al
B.1.639	EPI_ISL_2443791	USA/NY-NYGC-787-VTM1-OUGT620/2021	2021/1/19	New York Genome Center	Michael Zody et al
B.1.640	EPI_ISL_5592661	Congo/FCRM-100-A32.28.09.21/2021	2021/9/28	Fondation Congolaise pour la Recherche Médicale	Mfoutou Mapanguy Claujens Chastel et al
B.1.67	EPI_ISL_420985	Wales/PHWC-252B4/2020	2020/3/23	Public Health Wales Microbiology Cardiff	Catherine Moore et al
B.1.69	EPI_ISL_425808	Scotland/CVR78/2020	2020/3/6	COVID-19 Genomics UK (COG-UK) Consortium	Ana da Silva Filipe et al
B.1.70	EPI_ISL_425759	Scotland/CVR30/2020	2020/3/13	COVID-19 Genomics UK (COG-UK) Consortium	Ana da Silva Filipe et al
B.1.76	EPI_ISL_464467	England/201080091/2020	2020/3/5	Respiratory Virus Unit, Microbiology Services Colindale, Public Health England	PHE Covid Sequencing Team et al
B.1.77	EPI_ISL_532277	England/QEUH-52DB8F/2020	2020/5/18	Wellcome Sanger Institute for the COVID-19 Genomics UK (COG-UK) consortium	Harper VanSteenhouse et al
B.1.78	EPI_ISL_523523	Netherlands/un-EMC-767/2020	2020/3/12	Erasmus Medical Center	Bas Oude Munnink et al
B.1.8	EPI_ISL_414530	Netherlands/NoordBrabant_21/2020	2020/3/4	Erasmus Medical Center	David Nieuwenhuijse et al
B.1.81	EPI_ISL_440218	England/BRIS-121C9B/2020	2020/3/18	Wellcome Sanger Institute for the COVID-19 Genomics UK (COG-UK) consortium	Stephanie Hutchings et al
B.1.83	EPI_ISL_1029971	Belgium/MBLG51240/2020	2020/3/20	UCLouvain/IREC/MBLG	Jean Ruelle et al
B.1.84	EPI_ISL_940256	France/IDF_HB_112003109999/2020	2020/3/31	IAME UMR1137 Inserm, Université de Paris, Hôpital Bichat	Antoine Bridier-Nahmias et al
B.1.88.1	EPI_ISL_1586845	Iceland/1483/2020	2020/3/26	deCODE genetics	Daniel F Gudbjartsson et al

B.1.9	EPI_ISL_1499727	Togo/BMC-4-20/2020	2020/3/4	Unité Mixte Internationale TransVIHMI (UMI 233 IRD – U1175 INSERM - Université de Montpellier)IRD (Institut de recherche pour le développement)	Mounerou SALOU et al
B.1.9.1	EPI_ISL_453188	Scotland/EDB5108/2020	2020/3/31	COVID-19 Genomics UK (COG-UK) Consortium	McHugh M et al
B.1.9.2	EPI_ISL_467793	USA/VA-DCLS-0398/2020	2020/4/20	Virginia DCLS	Virginia DCLS et al
B.1.9.3	EPI_ISL_462180	Belgium/JBE-0317326/2020	2020/3/17	KU Leuven, Rega Institute, Clinical and Epidemiological Virology	Tony Wawina-Bokalanga et al
B.1.9.4	EPI_ISL_500920	Switzerland/BL-ETHZ-190059/2020	2020/7/9	Department of Biosystems Science and Engineering, ETH Zürich	Christian Beisel et al
B.1.9.5	EPI_ISL_417944	DRC/81/2020	2020/3/18	Pathogen Sequencing Lab, National Institute for Biomedical Research (INRB)	Placide Mbala-Kingebeni et al
B.1.91	EPI_ISL_451307	Italy/LOM-INMI-5314-N/2020	2020/2/21	Laboratory of Virology, INMI Lazzaro Spallanzani IRCCS	Fausto Baldanti et al
B.1.93	EPI_ISL_644683	France/OCC-SC467/2020	2020/3/6	CNR Virus des Infections Respiratoires - France SUD	Antonin Bal et al
B.1.94	EPI_ISL_419564	Luxembourg/LNS0366116/2020	2020/3/15	Laboratoire National de Santé, Microbiology, Epidemiology and Microbial Genomics	Anke Wienecke-Baldacchino et al
B.1.96	EPI_ISL_422601	Netherlands/NA_292/2020	2020/4/1	Erasmus Medical Center	Bas Oude Munnink et al
B.1.97	EPI_ISL_439884	England/CAMB-726F8/2020	2020/3/24	Wellcome Sanger Institute for the COVID-19 Genomics UK (COG-UK) consortium	Luke W Meredith et al
B.10	EPI_ISL_492694	England/BRIS-1309ED/2020	2020/3/12	Wellcome Sanger Institute for the COVID-19 Genomics UK (COG-UK) consortium	Stephanie Hutchings et al
B.11	EPI_ISL_413586	Netherlands/Tilburg_1363354/2020	2020/2/27	Erasmus Medical Center	David Nieuwenhuijse et al
B.12	EPI_ISL_479799	Japan/PG-0015/2020	2020/1/20	Pathogen Genomics Center, National Institute of Infectious Diseases	Tsuyoshi Sekizuka et al
B.13	EPI_ISL_436572	USA/WI-UW-278/2020	2020/4/3	University of Wisconsin-Madison AIDS Vaccine Research Laboratories	Gage Moreno et al
B.15	EPI_ISL_425480	England/NOTT-10DFD8/2020	2020/3/16	COVID-19 Genomics UK (COG-UK) Consortium	Gemma Clark et al
B.18	EPI_ISL_417553	Iceland/220/2020	2020/3/16	deCODE genetics	Daniel F Gudbjartsson et al
B.19	EPI_ISL_436672	USA/CA-CZB-1031/2020	2020/3/29	Chan-Zuckerberg Biohub	CZB Cliahub Consortium et al

B.20	EPI_ISL_437044	USA/CA-CZB-1044/2020	2020/3/31	Chan-Zuckerberg Biohub	CZB Cliahub Consortium et al
B.23	EPI_ISL_464328	England/20104013504/2020	2020/2/28	Respiratory Virus Unit, Microbiology Services Colindale, Public Health England	PHE Covid Sequencing Team et al
B.26	EPI_ISL_425701	Scotland/CVR162/2020	2020/3/17	COVID-19 Genomics UK (COG-UK) Consortium	Ana da Silva Filipe et al
B.28	EPI_ISL_464563	England/20109045404/2020	2020/3/6	Respiratory Virus Unit, Microbiology Services Colindale, Public Health England	PHE Covid Sequencing Team et al
B.29	EPI_ISL_415144	England/20102000106/2020	2020/2/27	Respiratory Virus Unit, Microbiology Services Colindale, Public Health England	Monica Galiano et al
B.3	EPI_ISL_414497	Germany/NW-HHU-02-1/2020	2020/2/25	Center of Medical Microbiology, Virology, and Hospital Hygiene, University of Duesseldorf	Ortwin Adams et al
B.3.1	EPI_ISL_416389	Shanghai/SH0093/2020	2020/1/21	National Research Center for Translational Medicine (Shanghai), Ruijin Hospital affiliated to Shanghai Jiao Tong University School of Medicine & Shanghai Public Health Clinical Center	Shengyue Wang et al
B.30	EPI_ISL_482451	USA/OR-PROV-216/2020	2020/3/9	Providence St. Joseph Health Molecular Genomics Laboratory	Alexa K Dowdell et al
B.31	EPI_ISL_456190	NewZealand/20VR1109/2020	2020/3/3	Institute of Environmental Science and Research (ESR)	Matt Storey et al
B.32	EPI_ISL_425776	Scotland/CVR47/2020	2020/3/13	COVID-19 Genomics UK (COG-UK) Consortium	Ana da Silva Filipe et al
B.33	EPI_ISL_408010	USA/CA-CDC-03040142-001/2020	2020/1/29	Pathogen Discovery, Respiratory Viruses Branch, Division of Viral Diseases, Centers for Disease Control and Prevention	Ying Tao et al
B.34	EPI_ISL_425768	Scotland/CVR40/2020	2020/3/13	COVID-19 Genomics UK (COG-UK) Consortium	Ana da Silva Filipe et al
B.35	EPI_ISL_420104	Singapore/27/2020	2020/3/5	Programme in Emerging Infectious Diseases, Duke-NUS Medical School	Danielle E Anderson et al
B.36	EPI_ISL_1712699	Qatar/QA-QU_02-2-44/2020	2020/3/13	Biomedical Research Center (BRC), Qatar University / Qatar Genome Project (QGP)	BRC: Fatiha M. Benslimane et al
B.37	EPI_ISL_631528	USA/NY-NYCPHL-000032/2020	2020/3/12	New York City Public Health Laboratory	Jade Wang et al

B.38	EPI_ISL_417008	Belgium/ULG-3662/2020	2020/3/7	GIGA Medical Genomics	Durkin Keith et al
B.39	EPI_ISL_464192	England/20098017104/2020	2020/2/27	Respiratory Virus Unit, Microbiology Services Colindale, Public Health England	PHE Covid Sequencing Team et al
B.4	EPI_ISL_412981	Wuhan/HBCDC-HB-05/2020	2020/1/18	Hubei Provincial Center for Disease Control and Prevention	Bin Fang et al
B.4.1	EPI_ISL_435045	Kazakhstan/NCB-1/2020	2020/3/22	RSE "National Center for Biotechnology"	Alexandr Shevtsov et al
B.4.2	EPI_ISL_435135	UnitedArabEmirates/L4682/2020	2020/2/25	Al Jalila Genomics Center	Ahmad Abou Tayoun et al
B.4.4	EPI_ISL_419753	Australia/VIC34/2020	2020/3/10	Victorian Infectious Diseases Reference Laboratory and Microbiological Diagnostic Unit Public Health Laboratory, Doherty Institute	Caly L. et al
B.4.5	EPI_ISL_419749	Australia/VIC30/2020	2020/3/10	Victorian Infectious Diseases Reference Laboratory and Microbiological Diagnostic Unit Public Health Laboratory, Doherty Institute	Caly L. et al
B.4.6	EPI_ISL_435121	UnitedArabEmirates/L0184/2020	2020/2/25	Al Jalila Genomics Center	Ahmad Abou Tayoun et al
B.4.7	EPI_ISL_435120	UnitedArabEmirates/L068/2020	2020/3/14	Al Jalila Genomics Center	Ahmad Abou Tayoun et al
B.40	EPI_ISL_412116	England/09c/2020	2020/2/9	Respiratory Virus Unit, Microbiology Services Colindale, Public Health England	Monica Galiano et al
B.41	EPI_ISL_1007665	SouthKorea/Chosun-CoV01_NP/2020	2020/2/6	Department of Internal Medicine, College of Medicine, Chosun University	Dong-Min Kim et al
B.42	EPI_ISL_497771	HongKong/HKU-200723-004/2020	2020/1/29	Department of Microbiology, The University of Hong Kong	Kelvin K.W. To et al
B.43	EPI_ISL_417187	HongKong/HKPU29_0102/2020	2020/2/8	Department of Health Technology and Informatics, Faculty of Health and Social Science, The Hong Kong Polytechnic University	Kenneth Siu-Sing LEUNG et al
B.44	EPI_ISL_418025	Portugal/PT0040/2020	2020/3/17	Instituto Nacional de Saude (INSA)	Guiomar et al
B.45	EPI_ISL_425762	Scotland/CVR33/2020	2020/3/13	COVID-19 Genomics UK (COG-UK) Consortium	Ana da Silva Filipe et al
B.46	EPI_ISL_604297	USA/CA-QDX-1986/2020	2020/3/18	Quest Diagnostics	Rosenthal et al
B.47	EPI_ISL_417296	England/20109098906/2020	2020/3/6	Respiratory Virus Unit, Microbiology Services Colindale, Public Health England	Monica Galiano et al

B.49	EPI_ISL_614437	Denmark/DCGC-978/2020	2020/3/9	Albertsen lab, Department of Chemistry and Bioscience, Aalborg University, Denmark	Danish Covid-19 Genome Consortia et al
B.5	EPI_ISL_2544696	Japan/KNG0073/2020	2020/2/5	Kanagawa Prefectural Institute of Public Health	Takayuki Hishiki et al
B.50	EPI_ISL_984277	Indonesia/KI-NIHRDC004160/2020	2020/3/14	National Institute of Health Research and Development	Kindi Adam et al
B.51	EPI_ISL_571259	USA/WI-QDX-439/2020	2020/3/18	Quest Diagnostics	Rosenthal et al
B.52	EPI_ISL_440349	England/CAMB-74061/2020	2020/3/18	Wellcome Sanger Institute for the COVID-19 Genomics UK (COG-UK) consortium	Luke W Meredith et al
B.53	EPI_ISL_582609	UnitedArabEmirates/skmc-0381517/2020	2020/2/7	Molecular/Surveillance lab Sheikh Khalifa Medical City	Amirtharaj Francis et al
B.55	EPI_ISL_451336	Sichuan/SC-NC-077/2020	2020/1/29	State Key Laboratory of Biotherapy of Sichuan University	Baowen Du et al
B.56	EPI_ISL_435142	UnitedArabEmirates/L9766/2020	2020/3/14	Al Jalila Genomics Center	Ahmad Abou Tayoun et al
B.57	EPI_ISL_416371	Shanghai/SH0068/2020	2020/2/5	National Research Center for Translational Medicine (Shanghai), Ruijin Hospital affiliated to Shanghai Jiao Tong University School of Medicine & Shanghai Public Health Clinical Center	Shengyue Wang et al
B.58	EPI_ISL_464404	England/201061034/2020	2020/3/4	Respiratory Virus Unit, Microbiology Services Colindale, Public Health England	PHE Covid Sequencing Team et al
B.6	EPI_ISL_422407	Taiwan/NTU04/2020	2020/3/4	Microbial Genomics Core Lab, National Taiwan University Centers of Genomic and Precision Medicine	Shiou-Hwei Yeh et al
B.6.1	EPI_ISL_2001656	Malaysia/NPHL_19105/2020	2020/3/11	National Public Health Laboratory Malaysia	Noorliza MN et al
B.6.2	EPI_ISL_490090	Malaysia/IMR_WC80031/2020	2020/5/23	Institute for Medical Research, Infectious Disease Research Centre, National Institutes of Health, Ministry of Health Malaysia	Suppiah J et al
B.6.3	EPI_ISL_429807	Canada/MB_2/2020	2020/3/10	National Microbiology Laboratory	Anna Majer et al
B.6.4	EPI_ISL_435689	Singapore/84/2020	2020/4/18	National Public Health Laboratory, National Centre for Infectious Diseases	Mak Tze Minn et al

B.6.5	EPI_ISL_500639	Australia/NSW2168/2020	2020/3/19	Area of Virology, Serology and Virology Division (SAViD), New South Wales Health Pathology Randwick	Rawlinson et al
B.6.6	EPI_ISL_5051470	Philippines/PH-RITM-0108/2020	2020/3/10	Research Institute for Tropical Medicine	Inez Andrea Medado et al
B.6.8	EPI_ISL_6463883	PapuaNewGuinea/PNG3146/2020	2020/7/13	Microbiological Diagnostic Unit - Public Health Laboratory (MDU-PHL)	Kumbu et al
B.60	EPI_ISL_418677	England/20122087702/2020	2020/3/15	Respiratory Virus Unit, Microbiology Services Colindale, Public Health England	Monica Galiano et al
B.61	EPI_ISL_464172	England/20079000404/2020	2020/2/14	Respiratory Virus Unit, Microbiology Services Colindale, Public Health England	PHE Covid Sequencing Team et al
C.1	EPI_ISL_3537060	USA/CA-CDPH1181/2020	2020/1/3	California Department of Public Health	CDPH IDLB COVIDNet et al
C.1.1	EPI_ISL_887438	Mozambique/INS-K007883/2020	2020/11/25	KRISP, KZN Research Innovation and Sequencing Platform	Nalia Ismael et al
C.1.2	EPI_ISL_2770450	SouthAfrica/KRISP-K018739/2021	2021/6/5	KRISP, KZN Research Innovation and Sequencing Platform	Florette Treurnicht et al
C.10	EPI_ISL_451646	Poland/Pom5/2020	2020/4/22	Laboratory of Recombinant Vaccines	Lukasz Rabalski et al
C.11	EPI_ISL_445350	Chile/RM-ISPCH-54/2020	2020/4/1	Instituto de Salud Publica de Chile	Andrés E Castillo et al
C.12	EPI_ISL_547977	NewZealand/20CV0068/2020	2020/8/12	Institute of Environmental Science and Research (ESR)	Xiaoyun Ren et al
C.13	EPI_ISL_1111163	Peru/ICA-INS-703/2020	2020/3/29	Laboratorio de Referencia Nacional de Enteropatógenos. Instituto Nacional de Salud del Perú	Ronnie Gavilan Chavez et al
C.14	EPI_ISL_1111162	Peru/ICA-INS-702/2020	2020/3/20	Laboratorio de Referencia Nacional de Enteropatógenos. Instituto Nacional de Salud del Perú	Ronnie Gavilan Chavez et al
C.17	EPI_ISL_479723	Egypt/CUNCI-HGC6I033/2020	2020/6/2	Egyptian National Cancer Institute (ENCI)	Zekri et al

C.18	EPI_ISL_1692799	Italy/CAM-TIGEM-IZSM-COLLI-9470/2020	2020/7/1	Telethon Institute of Genetics and Medicine (TIGEM)	Antonio Grimaldi Patrizia Annunziata Francesco Panariello Teresa Giuliano Michele Cennamo Valentina Bouche Chiara Colantuono Lucio Di Filippo Mariano Fiorenza Anna Manfredi Marcello Salvi Giuseppe Portella Andrea Ballabio Davide Cacchiarelli et al
C.19	EPI_ISL_747927	Denmark/DCGC-20010/2020	2020/12/7	Albertsen Lab, Department of Chemistry and Bioscience, Aalborg University, Denmark	Danish Covid-19 Genome Consortium et al
C.2	EPI_ISL_490282	SouthAfrica/R10517/2020	2020/6/9	National Institute for Communicable Diseases of the National Health Laboratory Service	Allam M et al
C.2.1	EPI_ISL_1014550	Curacao/CW-RIVM-21733/2020	2020/12/18	National Institute for Public Health and the Environment (RIVM)	Adam Meijer et al
C.20	EPI_ISL_617252	Denmark/DCGC-8213/2020	2020/10/26	Albertsen lab, Department of Chemistry and Bioscience, Aalborg University, Denmark	Danish Covid-19 Genome Consortia et al
C.21	EPI_ISL_804964	Spain/ML-ISCIII-202059850/2020	2020/9/15	Instituto de Salud Carlos III	Iglesias-Caballero et al
C.22	EPI_ISL_430808	Argentina/PAIS-A0017/2020	2020/4/3	Área de Secuenciación del Laboratorio de Virología del Hospital de Niños Dr. Ricardo Gutierrez on behalf of 'Proyecto Argentino Interinstitucional de genomica de SARS-CoV-2' (PAIS Consortium)	Nabaes Jodar et al
C.23	EPI_ISL_548944	Pakistan/UN-UVAS-Lahore-III/2020	2020/5/11	Institute of Microbiology, University of Veterinary and Animal sciences	Yaqub et al
C.25	EPI_ISL_529068	Peru/LIM-UPCH-0011/2020	2020/3/20	Laboratorio de Genómica Microbiana, Universidad Peruana Cayetano Heredia	Pablo Tsukayama et al
C.26	EPI_ISL_1167833	Chile/RM-44567/2020	2020/4/28	Instituto de Salud Publica de Chile	Javier Tognarelli et al
C.27	EPI_ISL_444426	England/CAMB-7FE7B/2020	2020/4/19	COVID-19 Genomics UK (COG-UK) Consortium	Luke W Meredith et al
C.28	EPI_ISL_537505	USA/CA-UCLA-17/2020	2020/4/30	Kruglyak Lab	Guo et al.
C.29	EPI_ISL_746564	Chile/RM-110525/2020	2020/6/25	Instituto de Salud Publica de Chile	Javier Tognarelli et al

C.3	EPI_ISL_559590	England/ALDP-49E20D/2020	2020/5/20	Wellcome Sanger Institute for the COVID-19 Genomics UK (COG-UK) consortium	The Lighthouse Lab in Alderley Park et al
C.30	EPI_ISL_532000	England/QEUH-960946/2020	2020/8/4	Wellcome Sanger Institute for the COVID-19 Genomics UK (COG-UK) consortium	Harper VanSteenhouse et al
C.30.1	EPI_ISL_619440	Denmark/DCGC-6540/2020	2020/10/5	Albertsen lab, Department of Chemistry and Bioscience, Aalborg University, Denmark	Danish Covid-19 Genome Consortia et al
C.31	EPI_ISL_1494152	USA/IL-IDPH-COO-S-0001755/2020	2020/8/11	Gagnon Lab, Southern Illinois University	Keith Gagnon et al
C.32	EPI_ISL_1111276	Peru/LIM-INS-378/2020	2020/5/7	Laboratorio de Referencia Nacional de Enteropatógenos. Instituto Nacional de Salud del Perú	Ronnie Gavilan Chavez et al
C.33	EPI_ISL_1532156	Peru/ICA-INS-757/2020	2020/5/18	Laboratorio de Referencia Nacional de Biotecnología y Biología Molecular. Instituto Nacional de Salud Perú	Carlos Padilla Rojas et al
C.34	EPI_ISL_968510	Canada/BC-BCCDC-6020/2020	2020/8/6	BCCDC Public Health Laboratory	Prystajecky Natalie et al
C.35	EPI_ISL_1111080	Peru/ANC-INS-620/2020	2020/3/31	Laboratorio de Referencia Nacional de Enteropatógenos. Instituto Nacional de Salud del Perú	Ronnie Gavilan Chavez et al
C.36	EPI_ISL_509726	USA/FL-BPHL-0668/2020	2020/3/13	Florida Bureau of Public Health Laboratories	Sarah Schmedes et al
C.36.1	EPI_ISL_1366018	Canada/Qc-L00292853/2020	2020/9/24	Laboratoire de santé publique du Québec	Sandrine Moreira et al
C.36.2	EPI_ISL_930860	Switzerland/SO-UHB-42489126/2020	2020/10/16	Clinical Bacteriology	Tim Roloff et al
C.36.3	EPI_ISL_3274160	Egypt/CPHL-A4/2021	2021/1/6	CPHL/MOH/EGYPT	Wael H. Roshdy/ Mohamed Kamal / Shymaa s. Ahmed/ Ramy Galal/Nancy el guindy/ Amel nagiub/yasser el hady/salma sayed/ Abd Monaem Adel/Galal Mahmoud/Dalia Ramadan/Rabeh ..R. El/Shesheny/ Mohamed A Ali/Mohamed Hassany et al
C.36.3.1	EPI_ISL_1464522	Germany/HH-HPI-p4792/2021	2021/3/4	Heinrich Pette Institute, Leibniz Institute for Experimental Virology	Alexis Robitaille et al

C.37.1	EPI_ISL_3023616	Peru/MDD-INS-1923/2021	2021/3/15	Laboratorio de Referencial Nacional de Virus Respiratorios	Carlos Padilla Rojas et al
C.38	EPI_ISL_1357071	Germany/BY-RKI-I-049645/2021	2021/2/16	Robert Koch Institute	?
C.39	EPI_ISL_2245832	Guinea/Conakry_IPG12108/2020	2020/9/23	Institut Pateur de Dakar	Grayo Solene et al
C.39	EPI_ISL_3401511	Peru/LAL-INS-2325/2021	2021/2/10	Laboratorio de Referencia Nacional de Virus Respiratorios. Centro Nacional de Salud Publica. Instituto Nacional de Salud Peru.	Carlos Padilla Rojas et al
C.4	EPI_ISL_1111257	Peru/LIM-INS-359/2020	2020/5/19	Laboratorio de Referencia Nacional de Enteropatógenos. Instituto Nacional de Salud del Perú	Ronnie Gavilan Chavez et al
C.5	EPI_ISL_541472	Switzerland/VD-ETHZ-270056/2020	2020/9/2	Department of Biosystems Science and Engineering, ETH Zürich	Christian Beisel et al
C.6	EPI_ISL_3068080	SouthAfrica/Tygerberg_79/2020	2020/4/18	Division of Medical Virology, Stellenbosch University and NHLS Tygerberg Hospital	Susan Engelbrecht et al
C.7	EPI_ISL_614513	Denmark/DCGC-1088/2020	2020/5/11	Albertsen lab, Department of Chemistry and Bioscience, Aalborg University, Denmark	Danish Covid-19 Genome Consortia et al
C.8	EPI_ISL_961669	Canada/NB-NML-240/2020	2020/3/31	National Microbiology Laboratory (NML)	Anna Majer et al
C.9	EPI_ISL_2444017	SouthAfrica/NICD-N00184/2020	2020/5/20	National Institute for Communicable Diseases of the National Health Laboratory Service	Amoako DG et al
D.2	EPI_ISL_480772	Australia/VIC2165/2020	2020/3/19	MDU-PHL	Seemann T. et al
D.3	EPI_ISL_521953	Australia/VIC2213/2020	2020/6/14	VIDRL and MDU-PHL	Caly L. et al
D.4	EPI_ISL_578302	Ireland/KK-NVRL-72IRL24802/2020	2020/8/13	National Virus Reference Laboratory	Michael Carr et al
D.5	EPI_ISL_1008424	Sweden/21-20401/2020	2020/10/12	The Public Health Agency of Sweden	Anna-Malin Linde et al
G.1	EPI_ISL_566478	England/MILK-9C2766/2020	2020/9/10	Wellcome Sanger Institute for the COVID-19 Genomics UK (COG-UK) consortium	The Lighthouse Lab in Milton Keynes et al
K.1	EPI_ISL_526708	SouthKorea/KCDC2774/2020	2020/7/23	Division of Viral Diseases, Center for Laboratory Control of Infectious Diseases, Korea Centers for Diseases Control and Prevention	Jeong-Min Kim et al
K.2	EPI_ISL_596354	Russia/PRI-RII-MH744S/2020	2020/8/7	WHO National Influenza Centre Russian Federation	Andrey Komissarov et al

K.3	EPI_ISL_590909	Norway/3271/2020	2020/8/29	Norwegian Institute of Public Health, Department of Virology	Kathrine Stene-Johansen et al
L.1	EPI_ISL_579578	Canada/NS-NML-948/2020	2020/3/26	National Microbiology Laboratory (NML)	Anna Majer et al
L.2	EPI_ISL_454766	Netherlands/Utrecht_10017/2020	2020/3/23	National Institute for Public Health and the Environment (RIVM)	Adam Meijer et al
L.3	EPI_ISL_729932	Nigeria/FC-CV247/2020	2020/7/8	African Centre of Excellence for Genomics of Infectious Diseases (ACEGID), Redeemer's University, Ede, Osun State, Nigeria	Oluniyi P.E. et al
L.4	EPI_ISL_1017303	USA/OR-PROV-301/2020	2020/6/29	Providence St. Joseph Health Molecular Genomics Laboratory	Alexa K Dowdell et al
M.1	EPI_ISL_516935	Moldova/ICGEB_MD4/2020	2020/6/18	International Centre for Genetic Engineering and Biotechnology (ICGEB) and ARGO Open Lab Platform for Genome Sequencing	Ulinici M et al
M.2	EPI_ISL_829812	Iceland/6134/2020	2020/10/26	deCODE genetics	Daniel F Gudbjartsson et al
M.3	EPI_ISL_551169	England/MILK-9A9C2A/2020	2020/9/1	Wellcome Sanger Institute for the COVID-19 Genomics UK (COG-UK) consortium	The Lighthouse Lab in Milton Keynes et al
N.1	EPI_ISL_460202	USA/MA-MGH-00076/2020	2020/3/25	Infectious Disease Program, Broad Institute of Harvard and MIT	Lemieux et al
N.2	EPI_ISL_6507361	FrenchGuiana/IPP8886/2020	2020/6/23	National Reference Center for Viruses of Respiratory Infections, Institut Pasteur, Paris	Mélanie Albert et al
N.3	EPI_ISL_430814	Argentina/PAIS-A0023/2020	2020/4/17	Área de Secuenciación del Laboratorio de Virología del Hospital de Niños Dr. Ricardo Gutierrez on behalf of 'Proyecto Argentino Interinstitucional de genomica de SARS-CoV-2' (PAIS Consortium)	Nabaes Jodar et al
N.4	EPI_ISL_586379	Canada/ON-S201/2020	2020/3/25	McMaster University	Allison McGeer et al
N.5	EPI_ISL_792335	Argentina/PAIS-A0255/2020	2020/4/25	Área de Secuenciación del Laboratorio de Virología del Hospital de Niños Dr. Ricardo Gutierrez on behalf of 'Proyecto Argentino Interinstitucional de genomica de SARS-CoV-2' (PAIS Consortium)	Nabaes Jodar et al
N.6	EPI_ISL_1167830	Chile/AR-265171/2020	2020/2/16	Instituto de Salud Publica de Chile	Javier Tognarelli et al
N.7	EPI_ISL_749151	Uruguay/TYT-M57/2020	2020/6/18	Institut Pasteur de Montevideo	Daiana Mir et al

N.8	EPI_ISL_806623	Kenya/C24022/2020	2020/6/23	KEMRI-Wellcome Trust Research Programme/KEMRI-CGMR-C Kilifi	Githinji et al
N.9	EPI_ISL_2241601	Brazil/PI-BA1200-584437/2020	2020/10/12	Coordenação Geral de Laboratórios de Saúde Pública (CGLAB/DAEVS/SVS/MS)	Vagner Fonseca et al
None	EPI_ISL_1423385	USA/NY-NYULH1008/2021	2021/2/23	Departments of Pathology and Medicine, New York University School of Medicine	Adriana Heguy et al
P.1	EPI_ISL_2249957	USA/IL-RED-FUL-39828238/2020	2020/4/7	Reditus Laboratories	Joshua J. Geltz et al
P.1	EPI_ISL_1404618	USA/MA-MASPHL-02518/2021	2021/3/18	Massachusetts State Public Health Laboratory	Andrew Lang et al
P.1.1	EPI_ISL_2775487	Brazil/PR-FIOCRUZ-33170-TG-165/2020	2020/12/30	Instituto Carlos Chagas - Fiocruz	Mauro de Medeiros Oliveira et al
P.1.1	EPI_ISL_5155963	USA/NY-P16G7/2021	2021/4/23	505 Irving Avenue, Syracuse, NY 13210 - Frank Middleton Lab	Frank Middleton et al
P.1.10	EPI_ISL_3912382	Brazil/CE-FIOCRUZ-00730/2021	2021/2/13	Analytical Competence Molecular Epidemiology Lab/ACME, Oswaldo Cruz Foundation, Ceara (FIOCRUZ CE)	Fabio Miyajima et al
P.1.10.2	EPI_ISL_2674658	Mexico/HID-InDRE_FB17208_S3171/2021	2021/5/23	Instituto de Diagnostico y Referencia Epidemiologicos (INDRE)	Claudia Wong-Arambula et al
P.1.11	EPI_ISL_2445049	Brazil/SP-IB_103317/2021	2021/5/10	Instituto Butantan	Dimas Tadeu Covas et al
P.1.12	EPI_ISL_3376385	Peru/LOR-INS-2202/2021	2021/1/2	Laboratorio de Referencial Nacional de Virus Respiratorios	Carlos Padilla Rojas et al
P.1.13	EPI_ISL_1420865	USA/FL-CDC-STM-000023348/2021	2021/2/24	Centers for Disease Control and Prevention Division of Viral Diseases, Pathogen Discovery	Peter W. Cook et al
P.1.14	EPI_ISL_1821208	Brazil/SP-2389/2020	2020/11/3	Instituto Adolfo Lutz, Interdiciplinary Procedures Center, Strategic Laboratory	Claudio Tavares Sacchi et al
P.1.15	EPI_ISL_2233906	Brazil/AM-CD1739/2020	2020/12/21	Scientific Platform Pasteur USP, University of Sao Paulo	Cunha et al
P.1.16	EPI_ISL_984622	Belgium/ULG-11925/2021	2021/1/31	GIGA Medical Genomics	Keith Durkin et al
P.1.17.1	EPI_ISL_3334239	Luxembourg/LNS8192483/2021	2021/4/2	Laboratoire national de sante, Microbiology, Microbial Genomics Platform	Anke Wienecke-Baldacchino et al
P.1.2	EPI_ISL_3664121	Brazil/PE-NXBS509GENOV827539448543/2021	2021/1/3	DASA	Rodrigo Guarischi et al

P.1.3	EPI_ISL_2777689	Brazil/AM-FIOCRUZ-21891957JM/2021	2021/3/12	Laboratorio de Ecologia de Doencas Transmissiveis na Amazonia, Instituto Leonidas e Maria Deane - Fiocruz Amazonia	Valdinete Nascimento et al
P.1.4	EPI_ISL_2777824	Brazil/AM-FIOCRUZ-21896338DCF/2021	2021/3/19	Laboratorio de Ecologia de Doencas Transmissiveis na Amazonia, Instituto Leonidas e Maria Deane - Fiocruz Amazonia	Valdinete Nascimento et al
P.1.5	EPI_ISL_2777660	Brazil/AM-FIOCRUZ-21891575VSC/2021	2021/4/3	Laboratorio de Ecologia de Doencas Transmissiveis na Amazonia, Instituto Leonidas e Maria Deane - Fiocruz Amazonia	Valdinete Nascimento et al
P.1.6	EPI_ISL_2777596	Brazil/AM-FIOCRUZ-21890984TSM/2021	2021/3/26	Laboratorio de Ecologia de Doencas Transmissiveis na Amazonia, Instituto Leonidas e Maria Deane - Fiocruz Amazonia	Valdinete Nascimento et al
P.1.8	EPI_ISL_3664270	Brazil/RJ-NXBS768GENOV827517602806/2021	2021/5/3	DASA	Rodrigo Guarischi et al
P.2	EPI_ISL_2157561	Brazil/RJ-FIOCRUZ-2188-1P/2020	2020/4/13	Laboratory of Respiratory Viruses and Measles, Oswaldo Cruz Institute, FIOCRUZ	Paola Resende et al
P.3	EPI_ISL_1597203	Netherlands/FL-RIVM-24324/2021	2021/2/27	National Institute for Public Health and the Environment (RIVM)	Adam Meijer et al
P.4	EPI_ISL_1625982	Brazil/SP-2281/2021	2021/2/17	Instituto Adolfo Lutz, Interdiciplinary Procedures Center, Strategic Laboratory	Claudio Tavares Sacchi et al
P.5	EPI_ISL_1445155	Brazil/SP-765479-NBD14/2021	2021/3/9	Instituto Butantan / Mendelics	Dimas Tadeu Covas et al
P.6	EPI_ISL_2965563	Uruguay/RT16-UYMO/2020	2020/12/2	Centro de Innovación en Vigilancia Epidemiológica (CiVE), Institut Pasteur Montevideo, Uruguay	Natalia Rego et al
P.7	EPI_ISL_5416087	Brazil/MG-LBIb1128/2020	2020/6/24	Laboratório de Biologia Integrativa / UFMG	Ana Valesca Fernandes Gilson Silva et al
Q.1	EPI_ISL_2903380	England/OXON-F8A254/2020	2020/12/19	COVID-19 Genomics UK (COG-UK) Consortium	Tanya Golubchik et al

Q.2	EPI_ISL_2020611	Italy/CAM-TIGEM-11334/2020	2020/12/15	TIGEM	Antonio Grimaldi Patrizia Annunziata Francesco Panariello Teresa Giuliano Michele Cennamo Valentina Bouche Chiara Colantuono Lucio Di Filippo Mariano Fiorenza Anna Manfredi Marcello Salvi Giuseppe Portella Andrea Ballabio Davide Cacchiarelli et al
Q.3	EPI_ISL_2628482	USA/CA-ALSR-101991/2020	2020/7/8	Andersen lab at Scripps Research	SEARCH Alliance San Diego with Ashleigh Murphy et al
Q.4	EPI_ISL_1730391	England/LOND-E652F/2020	2020/12/13	COVID-19 Genomics UK (COG-UK) Consortium	Sergi Castellano et al
Q.6	EPI_ISL_1262942	France/PDL-IPP04848/2021	2021/3/2	National Reference Center for Viruses of Respiratory Infections, Institut Pasteur, Paris	Marion Barbet et al
Q.7	EPI_ISL_1135025	France/ARA-HCL021022962101/2021	2021/1/29	CNR Virus des Infections Respiratoires - France SUD	Antonin Bal et al
Q.8	EPI_ISL_917989	Scotland/QEUH-10E71AF/2021	2021/1/21	Wellcome Sanger Institute for the COVID-19 Genomics UK (COG-UK) Consortium	Harper VanSteenhouse et al
R.1	EPI_ISL_2716627	SierraLeone/SL10/2020	2020/1/14	State Key Laboratory of Pathogen and Biosecurity, Beijing Institute of Microbiology and Epidemiology	Wurie et al
R.2	EPI_ISL_2284073	USA/MA-Yale-5158/2020	2020/12/22	Yale Center for Genomic Analysis	Shrikant Mane et al
S.1	EPI_ISL_552514	England/MILK-975243/2020	2020/8/21	Wellcome Sanger Institute for the COVID-19 Genomics UK (COG-UK) consortium	The Lighthouse Lab in Milton Keynes et al
U.1	EPI_ISL_838504	England/LIVE-DCEE25/2020	2020/12/15	COVID-19 Genomics UK (COG-UK) Consortium	Sam Haldenby et al
U.2	EPI_ISL_1469208	Sweden/RVFOI21-00682/2020	2020/11/11	CBRN Defence and Security, Swedish Defence Research Agency	Andreas Sjödin et al
U.3	EPI_ISL_1317736	Norway/2001/2021	2021/1/11	Norwegian Institute of Public Health, Department of Virology	Kathrine Stene-Johansen et al
V.1	EPI_ISL_822311	NorthernIreland/CAMC-A41A92/2020	2020/10/5	Wellcome Sanger Institute for the COVID-19 Genomics UK (COG-UK) Consortium	Rob Howes et al

V.2	EPI_ISL_650923	NorthernIreland/NIRE-10926A/2020	2020/10/20	COVID-19 Genomics UK (COG-UK) Consortium	Conall McCaughey et al
W.1	EPI_ISL_678350	Australia/NSW2802/2020	2020/10/2	Virology Research Laboratory; Area of Virology, Serology and Virology Division (SAViD), New South Wales Health Pathology Randwick	Foster et al
W.2	EPI_ISL_3426611	Netherlands/NB-MVD-CWGS2101771/2020	2020/9/23	Microvida	Suzan D. Pas et al
W.3	EPI_ISL_530531	England/QEUH-96DE86/2020	2020/8/18	Wellcome Sanger Institute for the COVID-19 Genomics UK (COG-UK) consortium	Harper VanSteenhouse et al
W.4	EPI_ISL_551840	England/CAMC-990C35/2020	2020/8/27	Wellcome Sanger Institute for the COVID-19 Genomics UK (COG-UK) consortium	Rob Howes et al
XA	EPI_ISL_989697	Wales/ALDP-11CF93B/2021	2021/1/30	Wellcome Sanger Institute for the COVID-19 Genomics UK (COG-UK) Consortium	Jacquelyn Wynn et al
XB	EPI_ISL_2628473	USA/SEARCH-101966/2020	2020/7/8	Andersen lab at Scripps Research	SEARCH Alliance San Diego with Ashleigh Murphy et al
Y.1	EPI_ISL_651724	England/CAMC-B23224/2020	2020/11/3	COVID-19 Genomics UK (COG-UK) Consortium	Thushan de Silva et al
Z.1	EPI_ISL_950606	England/NOTT-246E3F/2020	2020/6/2	COVID-19 Genomics UK (COG-UK) Consortium	Gemma Clark et al
