# Supplementary material for: Computation of Antigenicity Predicts SARS-CoV-2 Vaccine Breakthrough Variants
Source: Front Immunol. 2022 Mar 24;13:861050. doi: 10.3389/fimmu.2022.861050 (PMC8987580; doi:10.3389/fimmu.2022.861050)
Supplement: Supplementary file 5 [file Table_5.pdf]

Table S5 Data sources for Efficacy Data

Manufacturer	Vaccine	Test ID	Study Type	VE	Ref(DOI/Link)
AstraZeneca	ChAdOx1 nCoV-19	AZ	Phase3	0.81	10.1016/S0140-6736(21)00432-3
		US-AZ	Phase3	0.74	10.1056/NEJMoa2105290
		CL-AZ	RealWorld	0.71	https://cdn.who.int/media/docs/default-source/blue-print/chile_rafael-araos_who-vr-call_25oct2021.pdf?sfvrsn=7a7ca72a_7
		Scotland-Lancet-AZ	RealWorld	0.59	10.1016/S0140-6736(21)00677-2
		England-AZ	RealWorld	0.89	https://assets.publishing.service.gov.uk/government/uploads/system/uploads/attachment_data/file/990089/Vaccine_surveillance_report_-_week_20.pdf
BioNTech	BNT162b2	BNT	Phase3	0.95	10.1056/NEJMoa2034577
		CL-BNT	RealWorld	0.84	https://cdn.who.int/media/docs/default-source/blue-print/chile_rafael-araos_who-vr-call_25oct2021.pdf?sfvrsn=7a7ca72a_7
		Scotland-Lancet-BNT	RealWorld	0.77	10.1016/S0140-6736(21)00677-2
		IL-NEJM-BNT	RealWorld	0.94	10.1056/NEJMoa2101765
		IL-Lancet-BNT	RealWorld	0.97	10.1016/S0140-6736(21)00947-8
		URY-BNT	RealWorld	0.78	https://www.gub.uy/ministerio-salud-publica/comunicacion/noticias/segundo-estudio-efectividad-vacunacion-anti-sars-cov-2-uruguay-8-junio-2021
		England-BNT	RealWorld	0.85	https://assets.publishing.service.gov.uk/government/uploads/system/uploads/attachment_data/file/990089/Vaccine_surveillance_report_-_week_20.pdf
		US-BNT	RealWorld	0.90	10.15585/mmwr.mm7013e3
Cadila	ZyCoV-D	Cadila	Phase3	0.67	https://zyduscadila.com/public/pdf/pressrelease/ZyCoV_D_Press_Release_1_7_2021.pdf
Covaxin	BBV152	Covaxin	Phase3	0.78	10.1016/S0140-6736(21)02000-6
Johnson	Ad26.COV2.S	Johnson	Phase3	0.66	10.1056/NEJMoa2101544
		US-Johnson	Phase3	0.72	10.1056/NEJMoa2101544
		BR-Johnson	Phase3	0.68	10.1056/NEJMoa2101544
		SA-Johnson	Phase3	0.64	10.1056/NEJMoa2101544

Longcom	ZF2001	Longcom	Phase3	0.82	https://www.scmp.com/coronavirus/greater-china/article/3146729/chinese-3-shot-covid-19-vaccine-maker-says-trials-show-it
Moderna	mRNA-1273	Moderna	Phase3	0.94	10.1056/NEJMoa2035389
		US-Moderna	RealWorld	0.90	10.15585/mmwr.mm7013e3
Novavax	NVX-CoV2373	Novavax	Phase3	0.89	10.1126/science.abg8101
Sinopharm	BBIBP-CorV	Sinopharm	Phase3	0.78	10.1001/jama.2021.8565
SinoVac	CoronaVac	BR-SinoVac	Phase3	0.51	10.2139/ssrn.3822780
		TR-SinoVac	Phase3	0.84	10.1016/S0140-6736(21)01429-X
		BR2-SinoVac	RealWorld	0.80	https://apnews.com/article/caribbean-brazil-coronavirus-pandemic-business-health-20bd94d28ac7b373d7a8f3f9c557e5b6
		CL-SinoVac	RealWorld	0.54	https://cdn.who.int/media/docs/default-source/blue-print/chile_rafael-araos_who-vr-call_25oct2021.pdf?sfvrsn=7a7ca72a_7
		CL-NEJM-SinoVac	RealWorld	0.66	10.1056/NEJMoa2107715
		URY-SinoVac	RealWorld	0.60	https://www.gub.uy/ministerio-salud-publica/comunicacion/noticias/segundo-estudio-efectividad-vacunacion-anti-sars-cov-2-uruguay-8-junio-2021
		ID-SinoVac	RealWorld	0.94	https://sehatnegeriku.kemkes.go.id/baca/berita-utama/20210512/1937767/kajian-cepat-kemenkes-vaksin-sinovac-efektif-cegah-kematian/
Sputnik	rAd26-S+rAd5-S	Sputnik	Phase3	0.92	10.1016/S0140-6736(21)00234-8
		RU-Sputnik	RealWorld	0.98	https://rdif.ru/Eng_fullNews/6722/
		UAE-Sputnik	RealWorld	0.98	https://rdif.ru/Eng_fullNews/6919/
